# Internally Directed Stereochemical Alteration of Alkene Dihalogenation

**DOI:** 10.1021/acs.joc.6c00780

**Published:** 2026-07-28

**Authors:** Jeong Kyun Im, Chanwoo Nam, Jun-Ho Choi, Won-jin Chung

**Affiliations:** † Department of Chemistry, 65419 Gwangju Institute of Science and Technology, Gwangju 61005, Republic of Korea; ‡ KAIST InnoCORE AI-CRED Institute, Korea Advanced Institute of Science and Technology, Daejeon 34141, Republic of Korea

## Abstract

Electrophilic dihalogenation of alkenes is a powerful strategy for the construction of configurationally defined vicinal dihalides. However, because of its inherent *anti*-stereospecificity, the practical synthetic access has been largely restricted to only half of the diastereochemical space. Although recent advances enabled complementary *syn*-dihalogenations through sequences involving *anti*-addition followed by stereoinversion, these approaches often suffer from limitations when applied to aryl-substituted alkenes that are prone to loss of stereochemical integrity during electrophilic activation via stable benzylic cation formation. Our group previously reported an efficient *syn*-dichlorination of *N*-protected allylic amines by exploiting an internal assistance mechanism of a systematically identified stereodirecting group, which notably accommodated aryl alkenes. We present here a full account describing the detailed course of our study, including further efforts to expand the substrate and halogen scopes.

## Graphical abstract

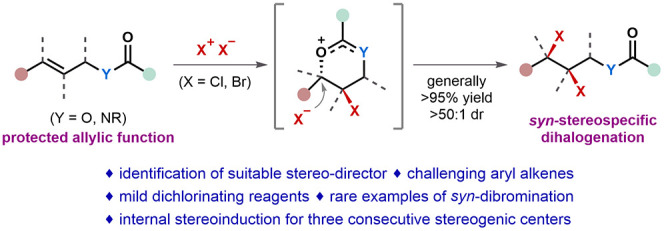

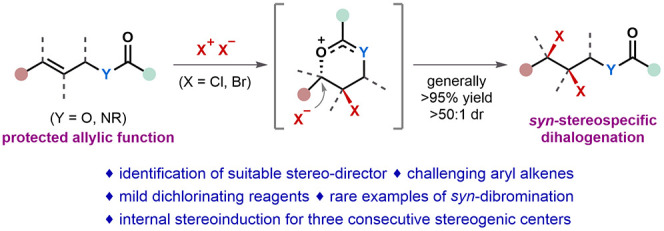

## Introduction

Anchimeric assistance or neighboring group participation is a widely known phenomenon that is often seen in electrophilically activated reactions, in which the involvement of a mildly Lewis basic group in close proximity to the reacting site leads to an unexpected alteration of the stereochemical outcome. For example, during the total synthesis of chlorosulfolipids by the groups of Vanderwal and Carreira, a stereoretentive opening of an epoxide intermediate **1** was observed as a result of double inversion caused by a neighboring chlorine substituent (**2**) (Figure 1A).^1^ If this phenomenon can be strategically exploited, a hidden stereoinversion in a classic reaction mechanism would provide a complementary stereospecificity. One such synthetic transformation with firmly established and thus, at the same time, inevitably restricted stereochemistry is *anti*-dihalogenation of alkenes.^2^ Despite the high predictability and the consequent synthetic utility,^3^ it is unfortunate that only half of the diastereochemical space is practically available. The access to *syn*-stereospecificity has long been an intriguing and formidable challenge, and a few ingenious solutions have been reported to date (Figure 1B).^4–7^ In most cases, the first halogen is introduced via a traditional *anti*-addition mechanism with a leaving group (**3**), which is then replaced by the second halogen via an S_N_2 mechanism to afford an overall *syn*-addition (**4**). This mechanism was first realized in *syn*-dichlorination by the Denmark group using an oxidized organoselenium that adds to an alkene then becomes a leaving group.^4^ Hypervalent iodine served a similar role for *syn*-difluorination in the works by the groups of Jacobsen and Gilmour,^5^ and a related *syn*-chlorofluorination was accomplished by the Lennox group in the presence of external chloride.^6^ Our group recently came up with a distinctive reaction mode, in which two sulfonium leaving groups are installed on the same side of the alkene (**5**) via concerted cycloaddition and then replaced sequentially by two halide nucleophiles (**6**).^8^ In this protocol, two different halides can be employed regiodivergently, achieving *syn*-bromochlorination with controlled site-selectivity.

**Figure 1:**
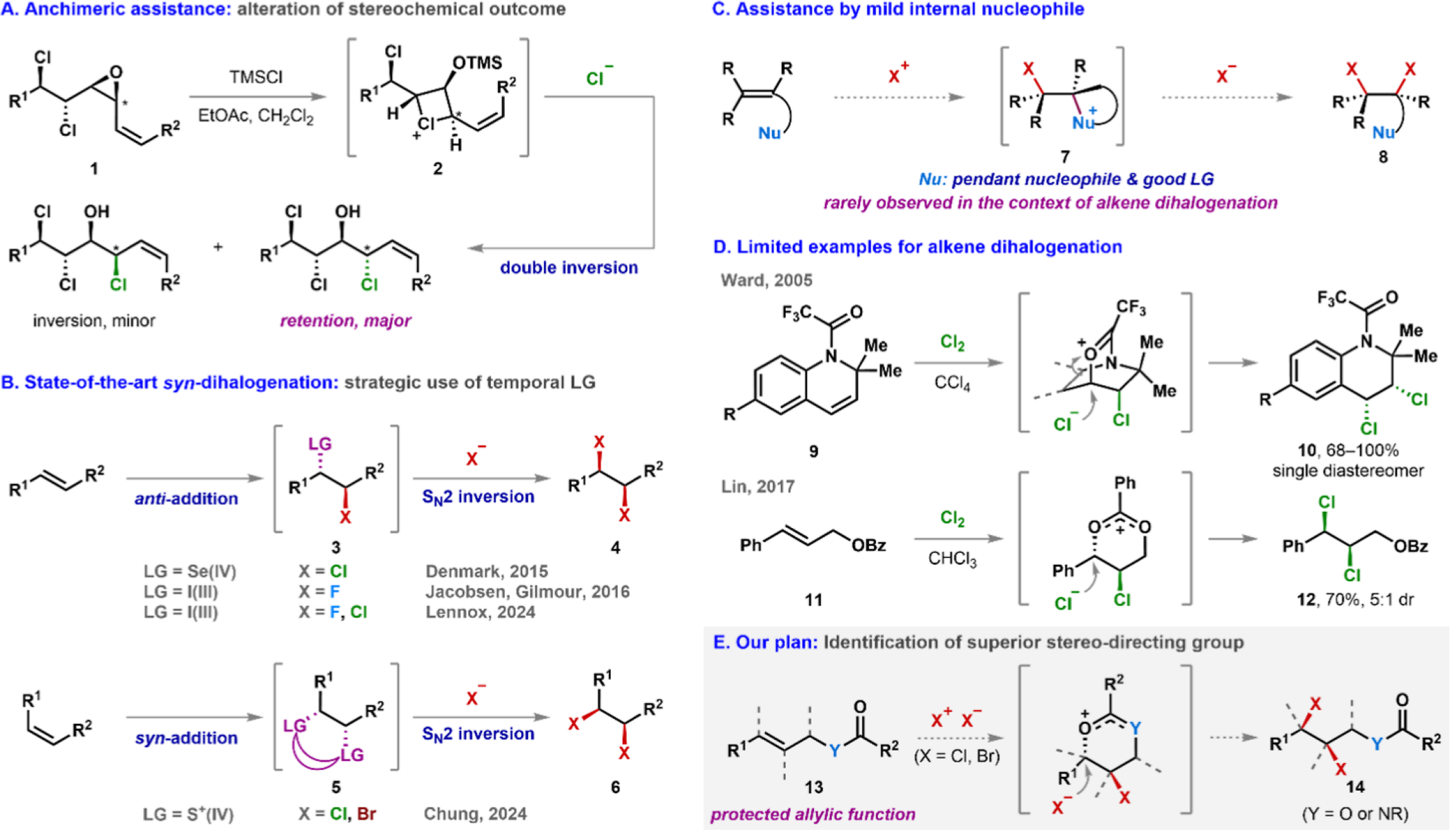
Research outline.

Concurrently, we also envisioned that the simplest mechanistic modification would be an addition of an inversion step to the traditional *anti*-dihalogenation process, as alluded to earlier, and anchimeric assistance appears logically suited for this purpose (Figure 1C). Such temporary participation of an internal nucleophile that can serve as a leaving group will enable a ring opening of the haliranium species (**7**), and then, the following displacement by halide at the inverted center would result in the introduction of two halogens from the same side (**8**). However, it is surprisingly difficult to find relevant examples in the context of alkene dihalogenation, and only a few notable precedents with ester-assisting groups are present in the chemical literature (Figure 1D).^9^ The Ward group observed the stereoaltering involvement of an *N*-acyl protecting group in the dichlorination of unsaturated cyclic amine **9** to form the *syn*-addition products **10**.^9a^ Despite the high stereospecificity, only limited structures were examined, and no further application of similar strategies was reported. Only as a supplementary piece of data, the Lin group described *syn*-dichlorination of a benzoyl-protected allylic alcohol **11** upon treatment with molecular chlorine.^9b^ A decent stereospecificity was observed (**12**), and the neighboring group participation was proposed for this unconventional outcome. Nonetheless, this intriguing result was used only as mechanistic information for their electrochemistry. As a part of our group’s interest in selective organic halogenations,^10^ we commenced an investigation on internally assisted *syn*-dihalogenation of alkenes with a special emphasis on finding a suitable pendant stereocontrolling group to access a practically useful selectivity level (Figure 1E). Allylic alcohols (Y = O) and amines (Y = NR) were chosen as naturally abundant and useful synthetic motifs, in which the internal nucleophile can be installed at a reasonable distance from the reacting site (**13**). The products would be stereodefined α,β-dihalo-alcohol or amine derivatives (**14**). A communication regarding our successful identification of a superb stereodirecting group for *syn*-dichlorination of disubstituted allylic amine derivatives was disclosed recently,^11^ and we present here a full account describing the detailed course of our study from the initial discovery to further scope expansion efforts with more densely substituted substrates and other halogen congeners, notably including a potential exploitation of internal stereoinduction and a rare example of *syn*-dibromination.

## Results and Discussion

### Optimization of Stereodirectors

For a safe and sustainable course of research, our first goal was to avoid the use of Cl_2_ gas. To that end, various other dichlorinating reagents were surveyed,^12^ and eventually, two convenient conditions with (COCl)_2_/Ph_2_SO^13^ or SO_2_Cl_2_ ^14^ were identified. Then, several carbonyl-based *O*-protecting groups were evaluated for dichlorination of (*E*)-cinnamyl alcohol (**13**) on the basis of the Lin group’s result as the starting point in search of efficient stereodirecting groups (Scheme 1).^9b^ Both acetate and benzoate afforded the *syn*-dichlorination products **14a** and **14b** with synthetically useful diastereoselectivity. The electronic effect appeared insignificant even with a highly perturbing *p*-CF_3_ (**14c**) or *p*-MeO (**14d**) substituent as well as a more nucleophilic carbamate function (**14e**), except for the reaction of **13c** in conditions B. However, the practicality was hampered by the formation of the 1,3-rearranged side product **15** that was difficult to remove.^9c^ The nonbenzylic oxygen originated from cinnamyl alcohol is also electrophilically activated in the halocyclized cationic intermediate, and thus it can serve as a competent nucleofuge, being responsible for the unwanted relocation of the *O*-protecting group.

**Scheme 1:**
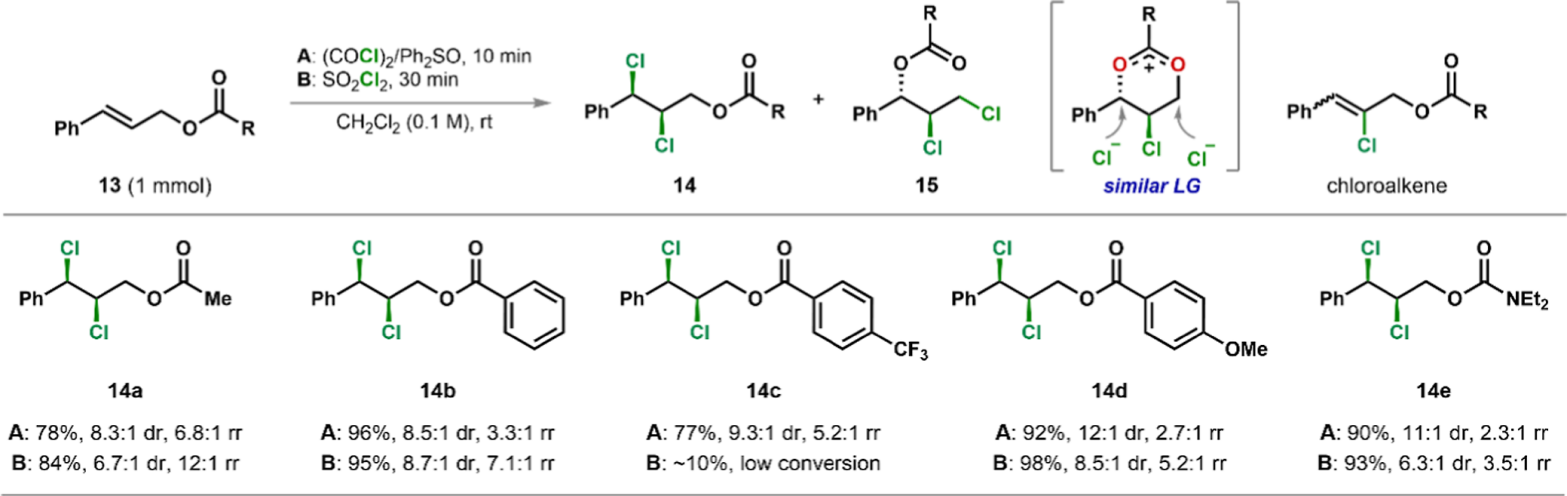
Survey of Carbonyl-Based *O*-Protecting Groups for *syn*-Dichlorination of (*E*)-Cinnamyl Alcohol^a^

a Performed with 1.2 equiv of each reagent. Corrected yields from chromatographed materials containing chloroalkene. Diastereomeric (*syn*/*anti*) and regioisomeric (**14**/**15**) ratios by ^1^H NMR analysis of the purified materials.

The electrophilic reactivity at the terminal carbon would be attenuated by introducing a nitrogen in place of the oxygen. Such a modification would also increase the Lewis basicity of the carbonyl group, potentially improving the trapping efficiency and the consequent stereospecificity. On the basis of this reasoning, imides were examined as common protected forms of cinnamyl amine (Scheme 2). Gratifyingly, phthalimide **16** behaved well to provide the desired product **21** as an essentially single isomer without the 1,3-rearrangement although a small amount of chloroalkene was generated via elimination in the halocyclized intermediate. Then, the imide structure was fine-tuned (**17** and **18**). Unfortunately, alteration of the electronic property in either direction did not lead to superior results (**22** and **23**). Instead, exceptional outcomes were gained by adjusting the imide ring size. When 1,8-naphthalimide **19a** was employed, **24a** was formed quantitatively under both conditions probably because of the strain relief in the halocyclized intermediate or the cationic charge delocalization over the extended aromatic π system. On the other hand, isomeric 2,3-naphthalimide **20** behaved similarly to phthalimide (**25**), implying that the ring size is more crucial than the charge delocalization, which can be rationalized by the degree of deviation from the ideal trigonal geometry in the central carbocation (**I** vs **II**). It is worth mentioning that the amenability of aryl alkene substrates is also a remarkable feature as the benzylic stabilization typically interferes with the cyclic haliranium formation, leading to the loss of the stereochemical information through the intermediacy of a discrete carbocation.^15^

**Scheme 2:**
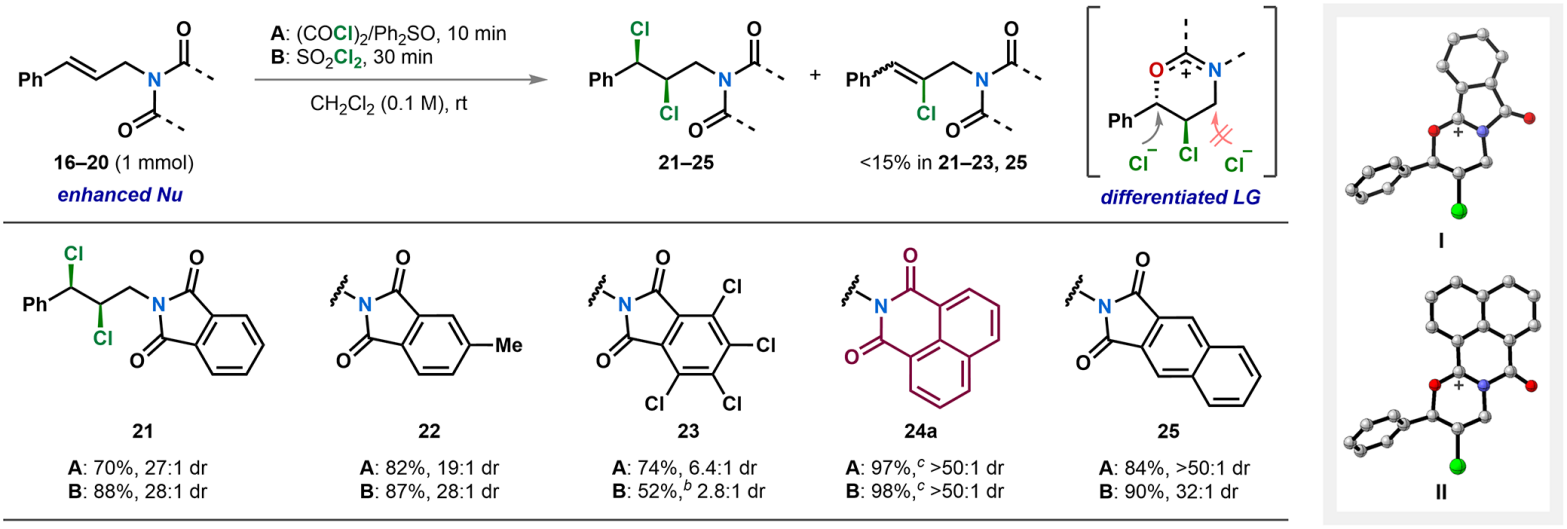
Evaluation of Imide-based *N*-Protecting Groups for *syn*-Dichlorination of (*E*)-Cinnamyl Amine^a^

a Performed with 1.2 equiv of each reagent. Corrected yields from chromatographed materials containing chloroalkene. Diastereomeric ratios by ^1^H NMR analysis of the purified materials.
b Low conversion.
c Isolated yields of the pure products.

### Substrate Survey

Under the two newly identified chlorinating conditions with the optimal 1,8-naphthalimide (NNpth) directing group, the substrate scope and limitations were explored (Scheme 3) with a wide range of *N*-allylic derivatives (Scheme 4). The relative stereochemical assignment was made by analogy with known 1,2-dichlorides (^3^ *J*_HH_ = 4.9–6.4 Hz for *syn*, 7.8–9.8 Hz for *anti*).^4a^ In general, the (COCl)_2_/Ph_2_SO combination showed better efficiency. Aromatic (*E*)-alkenes were insensitive to steric and electronic variations, consistently affording excellent yields and stereospecificity (**24a**–**24m**) except for the cases with a highly electron-donating alkoxy (**24g**) or phenoxy (**24h**) substituent, which favors the open carbocation formation through resonance-extended oxocarbenium-type stabilization. In addition, primary aliphatic (*E*)-alkenes exhibited respectable performances (**24n** and **24o**). However, the reaction was retarded by a bulky secondary alkyl group (**24p**), and a significant amount of chlorohydrin was observed after workup. In the cases of the (*Z*)-alkene isomers, whereas aromatic alkene substrates behaved poorly (**24q**), aliphatic alkenes maintained reasonable efficiencies (**24r** and **24s**). Furthermore, trisubstituted alkenes with an additional alkyl substituent at the α- or β-styryl positions (**24t** and **24u**) also showcased exceptional stereocontrol although these reactions were accompanied by chloroalkene or 1,3-dichlorinated alkene side products, which likely arose from the competing elimination in the chlorocyclized intermediate as the second chlorine attack at the benzylic position was hampered by increased steric hindrance. The *syn*-specific stereochemical course of our reaction was confirmed by X-ray crystallographic analysis of **24e**, **24t**, and **24u** (See the Supporting Information). The stereodirecting effect diminished substantially upon one-carbon extension of the tether length, causing almost complete loss of the stereospecificity (**24v**).

**Scheme 3:**
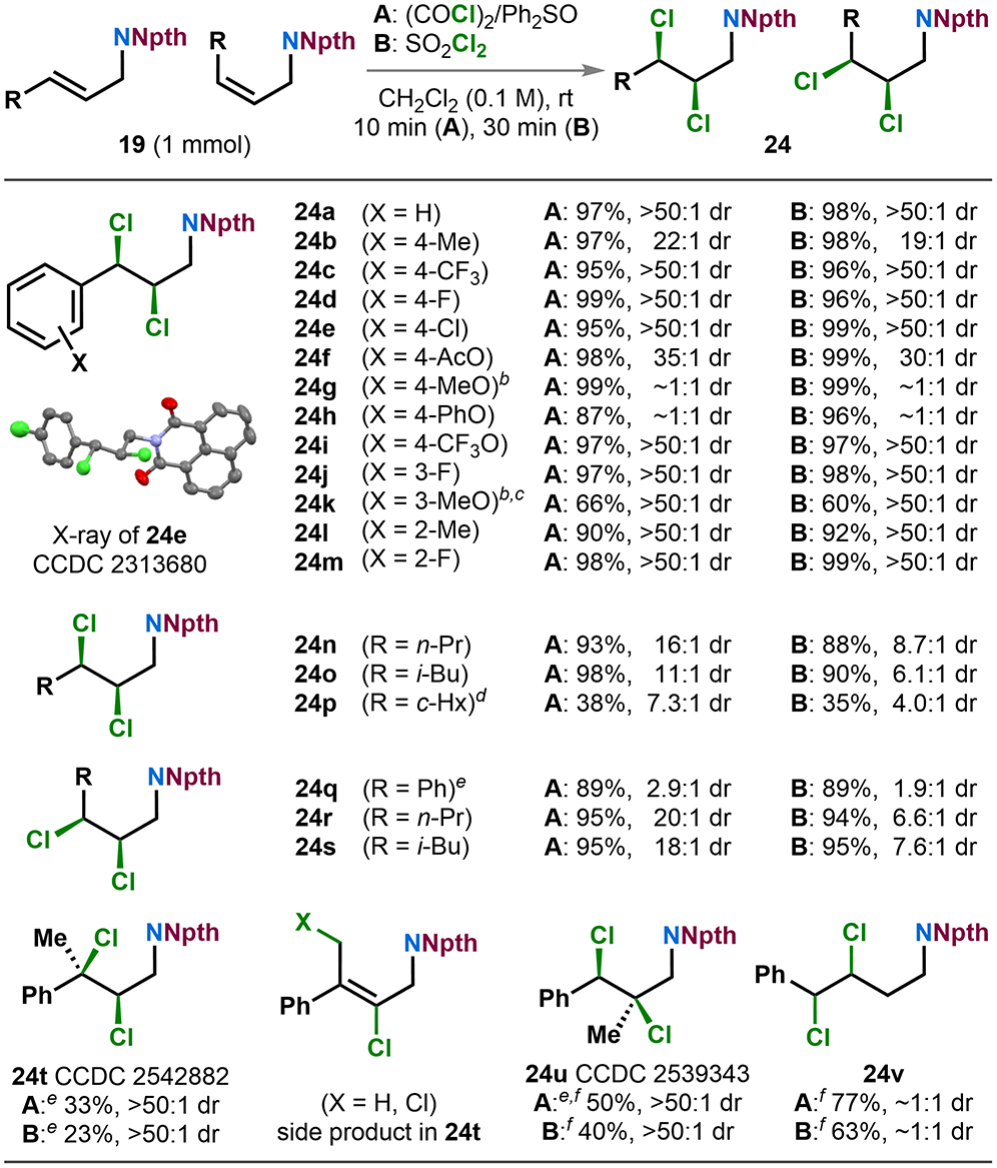
Substrate Scope and Limitations for *syn*-Dichlorination of Allylic 1,8-Naphthalimides (NNpth)[Fn s3fn7]

a Performed with 1.2 equiv of each reagent. Yields after column chromatography. Diastereomeric ratios by ^1^H NMR analysis of the purified materials, unless noted otherwise
b Analyzed by ^1^H NMR spectroscopy of the crude mixture with an internal standard.
c Mass loss by unidentifiable side products.
d Low reproducibility due to heterogeneity of the reaction mixture.
e Corrected yields from purified materials containing the side products.
f Yields after additional recrystallization.

**Scheme 4:**
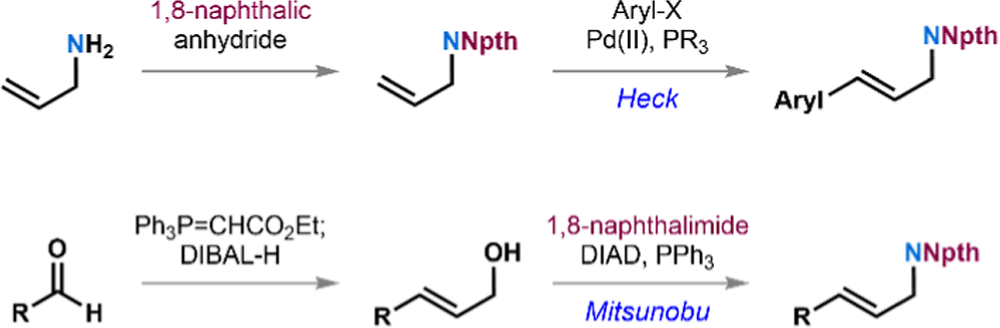
Preparative Methods for Allylic 1,8-Naphthalimides

### Efforts on the Reaction Scope Expansion

Asymmetric modification of the current method is challenging. However, it can still be envisioned to access enantioenriched dichlorides if an adjacent stereocenter can be exploited (Scheme 5). Then, three consecutive, heteroatom-containing stereogenic centers would be constructed from widely available chiral secondary allylic alcohol-derived starting materials. Accordingly, a chiral model substrate *rac*-**19w** was prepared and subjected to the standard conditions. Gratifyingly, **24w** was obtained with high diastereoselectivity, indicating an excellent internal stereoinduction. The major isomers could be crystallized out, and the *syn*-dichloride configuration was confirmed by X-ray crystallography.

**Scheme 5:**
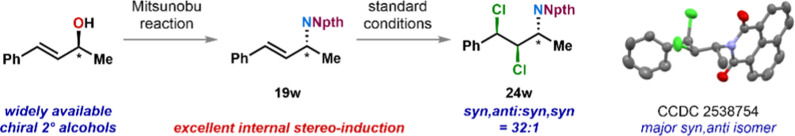
Evaluation of Internal Stereoinduction in a Chiral Secondary Allylic Substrate for Potential Access to Enantioenriched Dichlorides

**Scheme 6:**
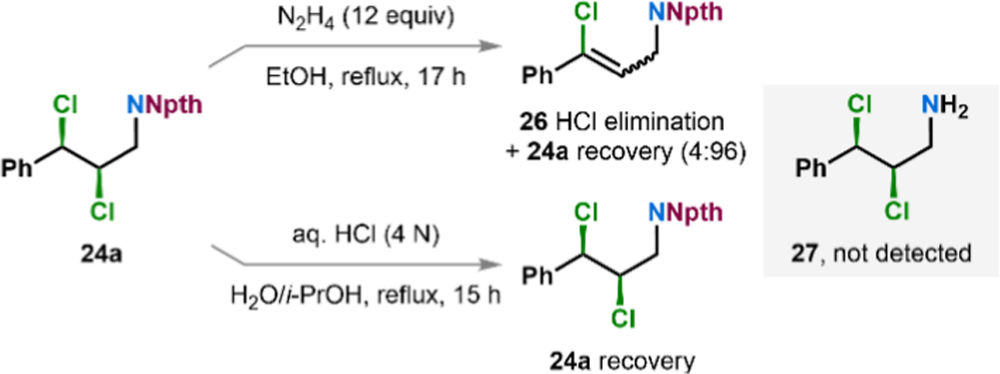
Representative Attempts at Naphthalimide Deprotection

Although NNpth displayed exceptional performance as an internal stereodirector, its removal was found problematic at a later stage of this study (Scheme 6). We naively assumed that naphthalimide would be deprotected similarly to phthalimide, but it was too robust and thus stable under the common imide deprotection conditions employing hydrazine. Other aminolysis (MeNH_2_, H_2_NCH_2_CH_2_NH_2_, HONH_2_, not shown) and acidic hydrolysis were also ineffective. Instead, the activated vicinal dichloride was destroyed more easily into styrenyl chloroalkene **26** without forming the desired free amine **27** or its ammonium salt. Nevertheless, our products may still find applications as fluorescent probes considering the wide utility of NNPth in both chemistry and biology.^16^

Subsequently, on the basis of the excellent performance of Cl_2_, structurally similar Br_2_ was examined (Scheme 7A). In this case, the internal assistance was generally less efficient (**28**–**30**), and the impact of the imide ring size was more pronounced. Whereas phthalimide behaved poorly (**29**), 1,8-naphthalimide still afforded high diastereoselectivity (**30**). A similar trend was observed with succinimide (**31**) and glutarimide (**32**). Although the higher reactivity of Br_2_ leads to lower activation barriers, as indicated by the computation (see below), resulting in less effective kinetic stereochemical differentiation, 1,8-naphthalimide maintains a decent stereodirecting efficiency (17:1 dr), showcasing its superiority. On the other hand, other diatomic halogenating reagents exhibited abnormal features (Scheme 7B). The internal assistant did not operate for ICl, leading to exclusive *anti*-addition (**33**), which was confirmed by X-ray crystallographic analysis. From IBr, only Br was incorporated into the product (**30**) with moderate *syn*-stereoselectivity. The origin of these unusual behaviors is currently unclear. In the case of XeF_2_, the starting material was partly recovered without difluorination.

**Scheme 7:**
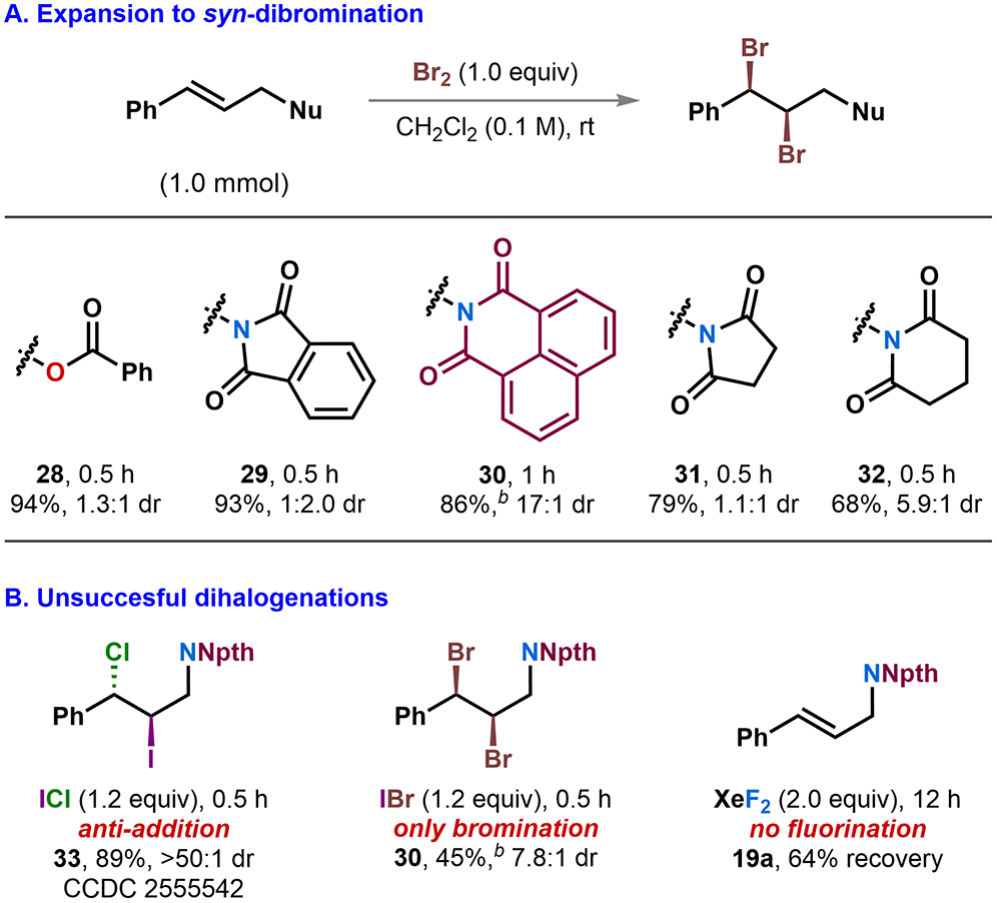
Evaluation of Other Halogens[Fn s7fn3]

a Yields after column chromatography. Diastereomeric ratios by ^1^H NMR analysis of the purified materials.
b Corrected yields from chromatographed materials containing the reactant **19a**.

### Computational Study

To formulate a reasonable reaction mechanism and interpret the experimental observations, DFT calculation was performed (Figure 2).^7e,17^ Initially, the representative (*E*)-cinnamyl substrate **19a** with the optimal NNpth was analyzed for the chlorination by SO_2_Cl_2_ (Figure 2A). As was known before, an open carbocation-like transition structure **19a**-**TS** was located instead of a three-membered cyclic chloriranium due to the pronounced stabilization caused by the presence of an aryl group. Therefore, both faces of the electrophilic carbon seem available for nucleophilic attack, which may lead to the loss of stereochemical information. Fortunately, the pendant NNpth approaches spontaneously from the other side to form a closed intermediate **19a**-**INT** upon the IRC calculation, ensuring the stereospecificity. It appears that the nucleophilic directing group and electrophilic halogen interact simultaneously with the styrenyl double bond, which is more favorable than the formation of a weak benzylic C–X bond in haliranium. The absence of excess external chloride nucleophile in our conditions must have been beneficial for the secure execution of this concerted asynchronous process.^18^ Then, the directing ability was compared with phthalimide (NPhth) (Figure 2B). Probably due to the geometrical constraint by the smaller five-membered cyclic imide structure, the NPhth group in **16** appears less capable of reaching the benzylic position, leading to the formation of a discrete carbocation **16**-**INT**-**1**, which may have caused the partial elimination to the chloroalkene side product. This ring strain argument is also consistent with the mediocre performance of 2,3-naphthalimide **25**. Nonetheless, the facile trapping by NPhth results in the minimal loss of stereospecificity in **16**-**INT**-**2** despite the stepwise nature. Subsequently, the observed substrate-specific behaviors were examined (Figure 2C). In (*Z*)-aryl alkene **19q**, the resonance charge delocalization is hampered by A_1,3_ steric encumbrance, and the out-of-plane aryl group hinders the incoming NNpth in **19q**-**TS**. These disadvantages are thought to be responsible for the poor stereospecificity. In the case of aliphatic substrate **19n**, the absence of benzylic stabilization leads to the usual formation of a three-membered haliranium **19n**-**INT**-**1**, which consequently prevents the simultaneous participation of the tethered nucleophile. Thus, it becomes possible for the external chloride to intervene during this delayed trapping, and the traditional *anti*-addition mechanism will be operative competitively to give the relatively moderate stereospecificity. Finally, the halogenating reagent-dependence was rationalized (Figure 2D). For alkyl alkene substrates, NNpth is present on the side of the approaching halogenating reagent, enabling an additional substrate–reagent interaction. Thus, the reaction of Ph_2_SCl is further promoted by favorable noncovalent π–π attraction, accounting for its superiority. The bromination with Br_2_ proceeds via a concerted TS similar to that of the chlorination, which again explains the high stereochemical conservation.

**Figure 2:**
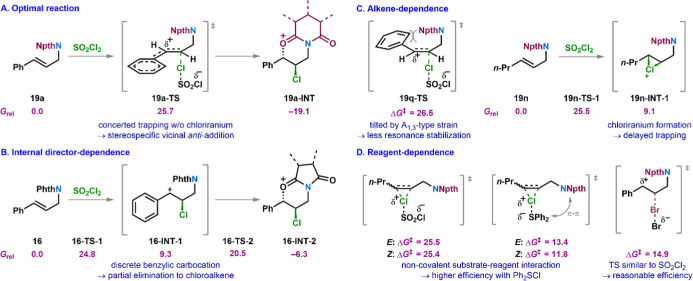
DFT calculation on the substrate and reagent-dependency (M06-2X/def2-TZVP/PCM­(CH_2_Cl_2_), Gibbs free energies in kcal/mol).

## Conclusions

Even though anchimeric assistance or neighboring group participation has long been known, its application to alkene dihalogenation is surprisingly scarce. In this work, upon scrutinizing various cinnamyl alcohol/amine derivatives, we logically identified the superior stereoaltering ability of 1,8-naphthalimide, which eventually enabled a practical internal assistance strategy for *syn*-dihalogenation of alkenes. Remarkably, nearly perfect yield (>95%) and selectivity (>50:1 dr) were obtained for aryl alkenes that had been notorious for their tendency to lose the stereochemical integrity during electrophilic activation due to benzylic cation formation. In addition, the use of mild and benign dichlorinating reagents (Ph_2_SO/(COCl)_2_ or SO_2_Cl_2_) in place of toxic Cl_2_ gas allowed for the operational convenience as well as the broad functional group compatibility. The scope and limitations of the current method were also examined with aliphatic and trisubstituted alkenes. Moreover, the potential expansions to asymmetric variants using chiral substrates and other halogens such as Br_2_ were also evaluated. Furthermore, the DFT calculation located a stereospecific, concerted halocyclization pathway promoted by the pendant 1,8-naphthalimide nucleophile without the generation of a carbocation intermediate, and the impact of the three key components, stereodirector, substrate structure, and halogenating reagent, was probed. Consequently, a formerly unavailable stereoguiding feature was successfully demonstrated for simple, highly efficient alkene *syn*-dihalogenation.

## Experimental Section

### General Experimental

All reactions were performed in flame-dried glassware under an atmosphere of dry argon unless otherwise noted. Purification of solvents and reagents is described in the Supporting Information. Merck Millipore silica gel (SiO_2_) 60 (0.040–0.063 mm) was used for column chromatography. Merck Millipore TLC silica gel 60 F_254_ was used for thin-layer chromatography (TLC). Visualization of TLC was accomplished with UV (254 nm) as well as potassium permanganate (KMnO_4_) and ceric ammonium molybdate (CAM) staining solutions. Kugelrohr distillation was performed by Büchi B585 glass oven with Büchi bulb-to-bulb distillation apparatus, and air-bath temperatures (ABT) are reported. ^1^H, ^13^C­{^1^H}, and ^19^F NMR spectra were recorded on a JEOL ECS400 spectrometer (400 MHz, ^1^H; 100 MHz, ^13^C; 376 MHz, ^19^F). Chemical shifts are referenced to residual chloroform (CHCl_3_, 7.26 ppm, ^1^H; 77.16 ppm, ^13^C), acetone ((CH_3_)_2_CO, 2.05 ppm, ^1^H; 29.84 ppm, ^13^C), dimethyl sulfoxide ((CH_3_)_2_SO, 39.52 ppm, ^13^C), and hexafluorobenzene (C_6_F_6_, −164.9 ppm, ^19^F). Chemical shifts are reported in ppm, and multiplicities are indicated by s (singlet), d (doublet), t (triplet), q (quartet), bs (broad singlet), and m (multiplet). Coupling constants, *J*, are reported in Hertz. ESI-HRMS was performed on a Bruker Impact II quadrupole-time-of-flight (Q-TOF) spectrometer at GIST Advanced Institute of Instrumental Analysis (GAIA). X-ray crystallographic data for **24t**, **24u**, **24w**, and **33** were collected using a Rigaku Oxford Diffraction XtaLAB Synergy-S diffractometer using CuKα radiation (λ = 1.54184 Å) at GAIA. The syntheses of **13b**, **14b**, **16**, **18**, **19a**–**o**, **19q**–**s**, **21**, **23**, **24a**–**o**, and **24q**–**s** were described previously.^11^ (*E*)-3-cyclohexylprop-2-en-1-ol,^19^ (*E*)-3-phenylbut-2-en-1-ol,^20^ (*E*)-2-methyl-3-phenylprop-2-en-1-ol,^21^ (*E*)-4-phenylbut-3-en-1-ol,^22^ (*E*)-4-phenylbut-3-en-2-ol,^23^ **13a**,^24^ and **13d** ^25^ were prepared following reported procedures.

### Preparation of Substrates

#### (*E*)-Cinnamyl 4-(trifluoromethyl)­benzoate (**13c**)

To a stirred solution of DMAP (49 mg, 0.40 mmol, 0.1 equiv), Et_3_N (670 μL, 4.8 mmol, 1.2 equiv), and 4-(trifluoromethyl)­benzoyl chloride (710 μL, 4.8 mmol, 1.2 equiv) in CH_2_Cl_2_ (20 mL), was added (*E*)-cinnamyl alcohol (541 mg, 4.00 mmol, 1.0 equiv) at rt. After 2 h, sat. aq. NaHCO_3_ (40 mL) was added. The organic layer was separated, and the aqueous layer was extracted with CH_2_Cl_2_ (40 mL × 3). The combined organic layers were dried over anhydrous Na_2_SO_4_, filtered through a glass frit, and concentrated under reduced pressure. Column chromatography (SiO_2_, ϕ = 3.0 cm, *l* = 11 cm, CH_2_Cl_2_/hexanes = 1/4, *R*_*f*_ = 0.2, UV 254 nm) and bulb-to-bulb distillation (500 mTorr, ABT 290–300 °C) afforded **13c** as white solid (1.21 g, 99%). Data for **13c**:^26^ ^1^H NMR (400 MHz, CDCl_3_): δ 8.20 (d, *J* = 8.2, 2H), 7.72 (d, *J* = 8.2, 2H), 7.43 (d, *J* = 6.9, 2H), 7.34 (appr. t, *J* = 7.3, 2H), 7.28 (appr. t, *J* = 7.8, 1H), 6.76 (d, *J* = 16.0, 1H), 6.41 (dt, *J* = 16.0, 6.4, 1H), 5.02 (dd, *J* = 6.6, 1.1, 2H); ^19^F NMR (376 MHz, CDCl_3_): δ −66.3.

#### (*E*)-Cinnamyl Diethylcarbamate (**13e**)

To a stirred solution of DMAP (90 mg, 0.74 mmol, 0.1 equiv), Et_3_N (1.1 mL, 8.2 mmol, 1.2 equiv), and diethylcarbamoyl chloride (1.0 mL, 8.2 mmol, 1.2 equiv) in CH_2_Cl_2_ (14 mL), was added (*E*)-cinnamyl alcohol (845 mg, 6.80 mmol, 1.0 equiv) at rt. After being refluxed in an oil bath for 45 h (unoptimized), the reaction mixture was cooled to rt, and sat. aq. NaHCO_3_ (60 mL) was added. The organic layer was separated, and the aqueous layer was extracted with CH_2_Cl_2_ (60 mL × 3). The combined organic layers were dried over anhydrous Na_2_SO_4_, filtered through a glass frit, and concentrated under reduced pressure. Column chromatography (SiO_2_, ϕ = 3.5 cm, *l* = 11 cm, EtOAc/hexanes = 1/10, *R*_*f*_ = 0.2, UV 254 nm) and bulb-to-bulb distillation (150 mTorr, ABT 170–220 °C) afforded **13e** as a colorless oil (818 mg, 52%). Data for **13e**:^27^ ^1^H NMR (400 MHz, CDCl_3_): δ 7.40 (d, *J* = 7.3, 2H), 7.32 (t, *J* = 7.6, 2H), 7.27–7.23 (m, 1H), 6.63 (d, *J* = 16.0, 1H), 6.32 (dt, *J* = 16.0, 6.2, 1H), 4.75 (dd, *J* = 6.4, 1.4, 2H), 3.31 (br s, 4H), 1.14 (t, *J* = 7.1, 6H).

#### *N*-Allyl-4-methylphthalimide (**S1**)

To a stirred solution of 4-methylphthalic anhydride (653 mg, 4.03 mmol, 1.4 equiv) in PhMe (17 mL), was added allylamine (220 μL, 2.9 mmol, 1.0 equiv) at rt. After being heated at 160 °C in an oil bath with a Dean–Stark apparatus for 16 h (unoptimized), the reaction mixture was cooled to rt and concentrated under reduced pressure. Column chromatography (SiO_2_, ϕ = 4.0 cm, *l* = 9.0 cm, CH_2_Cl_2_, *R*_*f*_ = 0.7, UV 254 nm) and recrystallization (hexanes) afforded **S1** as white crystals (336 mg, 56%). Data for **S1**:^28^ mp 68.5–69.7 °C; ^1^H NMR (400 MHz, CDCl_3_): δ 7.73 (d, *J* = 7.8, 1H), 7.65 (s, 1H), 7.50 (d, *J* = 6.9, 1H), 5.93–5.83 (m, 1H), 5.23 (dd, *J* = 17.2, 1.1, 1H), 5.18 (dd, *J* = 10.1, 1.4, 1H), 4.27 (dt, *J* = 5.8, 1.5, 2H), 2.51 (s, 3H).

#### *N*-(*E*)-Cinnamyl-4-methylphthalimide (**17**)

To a stirred solution of **S1** (876 mg, 4.35 mmol, 1.0 equiv), Pd­(OAc)_2_ (9 mg, 0.04 mmol, 0.01 equiv), and P­(*o*-Tol)_3_ (24 mg, 0.078 mmol, 0.02 equiv) in Et_3_N (1.8 mL, 13 mmol, 3.1 equiv), was added PhI (0.47 mL, 4.2 mmol, 1.0 equiv) at rt. After being heated at 110 °C in an oil bath for 19 h (unoptimized), the reaction mixture was cooled to rt, and brine (50 mL) was added. The organic layer was separated, and the aqueous layer was extracted with CH_2_Cl_2_ (50 mL × 3). The combined organic layers were dried over anhydrous MgSO_4_, filtered through a glass frit, and concentrated under reduced pressure. Column chromatography (SiO_2_, ϕ = 5.0 cm, *l* = 11 cm, CH_2_Cl_2_/hexanes = 1/1, *R*_*f*_ = 0.2, UV 254 nm) and recrystallization (CH_2_Cl_2_/hexanes) afforded **17** as white crystals (670 mg, 57%). Data for **17**:^29^ mp 127.0–129.6 °C; ^1^H NMR (400 MHz, CDCl_3_): δ 7.74 (d, *J* = 7.8, 1H), 7.66 (s, 1H), 7.51 (d, *J* = 7.8, 1H), 7.35 (d, *J* = 6.9, 2H), 7.30–7.26 (m, 2H), 7.22 (appr. t, *J* = 7.1, 1H), 6.64 (d, *J* = 16.0, 1H), 6.25 (dt, *J* = 15.7, 6.5, 1H), 4.43 (dd, *J* = 6.4, 1.4, 2H), 2.51 (s, 3H).

#### *N*-Allyl-2,3-naphthalimide (**S2**)

To a stirred solution of 2,3-naphthalenedicarboxylic acid (2.60 g, 12.0 mmol, 1.0 equiv) in AcOH (240 mL), was added allylamine (18 mL, 240 mmol, 20 equiv). After being refluxed in an oil bath for 16 h (unoptimized), the reaction mixture was cooled to rt, and sat. aq. NaHCO_3_ (240 mL) was added. The organic layer was separated, and the aqueous layer was extracted with CH_2_Cl_2_ (240 mL × 3). The combined organic layers were dried over anhydrous MgSO_4_, filtered through a glass frit, and concentrated under reduced pressure. Column chromatography (SiO_2_, ϕ = 3.5 cm, *l* = 9.0 cm, CH_2_Cl_2_/hexanes = 1/1, *R*_*f*_ = 0.6, UV 254 nm) and recrystallization (hexanes) afforded **S2** as white crystals (2.21 g, 78%). Data for **S2**: mp 148.1–149.2 °C; ^1^H NMR (400 MHz, CDCl_3_): δ 8.33 (s, 2H), 8.04 (dd, *J* = 6.2, 3.4, 2H), 7.69 (dd, *J* = 6.0, 3.2, 2H), 5.98–5.88 (m, 1H), 5.28 (dd, *J* = 16.9, 1.4, 1H), 5.22 (dd, *J* = 10.1, 0.9, 1H), 4.36 (d, *J* = 5.5, 2H); ^13^C­{^1^H} NMR (100 MHz, CDCl_3_): δ 167.8, 135.6, 131.6, 130.4, 129.3, 127.9, 124.9, 118.0, 40.4; HRMS (ESI) *m*/*z*: [M + Na]^+^ calcd for C_15_H_11_NO_2_Na 260.0687; found, 260.0683.

#### *N*-(*E*)-Cinnamyl-2,3-naphthalimide (**20**)

To a stirred solution of **S2** (732 mg, 3.08 mmol, 1.0 equiv), Pd­(OAc)_2_ (10 mg, 0.044 mmol, 0.01 equiv), and P­(*o*-Tol)_3_ (18 mg, 0.059 mmol, 0.02 equiv) in Et_3_N (4.2 mL, 30 mmol, 10 equiv), was added PhI (340 μL, 3.0 mmol, 1.0 equiv) at rt. After being heated at 110 °C in an oil bath for 15 h (unoptimized), the reaction mixture was cooled to rt, and brine (30 mL) was added. The organic layer was separated, and the aqueous layer was extracted with CH_2_Cl_2_ (30 mL × 3). The combined organic layers were dried over anhydrous MgSO_4_, filtered through a glass frit, and concentrated under reduced pressure. Column chromatography (SiO_2_, ϕ = 3.0 cm, *l* = 6.0 cm, CH_2_Cl_2_/hexanes = 1/1, *R*_*f*_ = 0.3, UV 254 nm) and recrystallization (CH_2_Cl_2_/hexanes) afforded **20** as white crystals (506 mg, 54%). Data for **20**: mp 230.6–231.8 °C; ^1^H NMR (400 MHz, CDCl_3_): δ 8.36 (s, 2H), 8.06 (dd, *J* = 5.8, 3.4, 2H), 7.70 (dd, *J* = 6.1, 3.1, 2H), 7.37 (d, *J* = 7.9, 2H), 7.30–7.20 (m, 3H), 6.70 (d, *J* = 15.6, 1H), 6.31 (dt, *J* = 15.7, 6.6, 1H), 4.52 (d, *J* = 6.7, 2H); ^13^C­{^1^H} NMR (100 MHz, CDCl_3_): δ 167.8, 136.6, 135.8, 134.2, 130.5, 129.3, 128.7, 128.2, 128.0, 126.8, 124.9, 123.0, 40.1; HRMS (ESI) *m*/*z*: [M + Na]^+^ calcd for C_21_H_15_NO_2_Na 336.1000; found, 336.0994.

#### *N*-((*E*)-3-Cyclohexylprop-2-en-1-yl)-1,8-naphthalimide (**19p**)

To a stirred mixture of 1,8-naphthalimide (783 mg, 3.97 mmol, 1.2 equiv), PPh_3_ (1.03 g, 3.93 mmol, 1.2 equiv), and (*E*)-3-cyclohexylprop-2-en-1-ol (459 mg, 3.27 mmol, 1.0 equiv) in THF (15 mL), was added DIAD (960 μL, 4.9 mmol, 1.5 equiv) at 0 °C dropwise. The reaction mixture was stirred at rt for 46 h (unoptimized), and brine (30 mL) was added. The organic layer was separated, and the aqueous layer was extracted with EtOAc (30 mL × 3). The combined organic layers were dried over anhydrous MgSO_4_, filtered through a glass frit, and concentrated under reduced pressure. Multiple column chromatography (SiO_2_, ϕ = 3.5 cm, *l* = 10 cm, CH_2_Cl_2_/hexanes = 1/1, *R*_*f*_ = 0.3, UV = 254 nm) and recrystallization (CH_2_Cl_2_/hexanes) afforded **19p** as white crystals (463 mg, 44%). Data for **19p**: mp 131.5–132.1 °C; ^1^H NMR (400 MHz, CDCl_3_): δ 8.45 (d, *J* = 7.3, 2H), 8.07 (d, *J* = 8.2, 2H), 7.63 (appr. t, *J* = 7.8, 2H), 5.79 (dd, *J* = 15.6, 6.1, 1H), 5.59–5.52 (m, 1H), 4.67 (d, *J* = 6.4, 2H), 1.90–1.89 (m, 1H), 1.67–1.54 (m, 5H), 1.23–0.97 (m, 5H); ^13^C­{^1^H} NMR (100 MHz, CDCl_3_): δ 163.7, 141.1, 133.7, 131.4, 131.0, 128.0, 126.8, 122.6, 121.2, 42.1, 40.2, 32.6, 26.2, 26.0; HRMS (ESI) *m*/*z*: [M + Na]^+^ calcd for C_21_H_21_NO_2_Na 342.1470; found, 342.1464.

#### *N*-(*E*)-β-methylcinnamyl-1,8-naphthalimide (**19t**)

To a stirred mixture of 1,8-naphthalimide (237 mg, 1.20 mmol, 1.2 equiv), PPh_3_ (315 mg, 1.20 mmol, 1.2 equiv), and (*E*)-3-phenylbut-2-en-1-ol (148 mg, 1.00 mmol, 1.0 equiv) in THF (4 mL), was added DIAD (300 μL, 1.5 mmol, 1.5 equiv) at 0 °C dropwise. The reaction mixture was stirred at rt for 12 h (unoptimized), and brine (10 mL) was added. The organic layer was separated, and the aqueous layer was extracted with EtOAc (10 mL × 3). The combined organic layers were dried over anhydrous MgSO_4_, filtered through a glass frit, and concentrated under reduced pressure. Column chromatography (SiO_2_, ϕ = 3.5 cm, *l* = 12 cm, CH_2_Cl_2_/hexanes = 1/1, *R*_*f*_ = 0.4, UV 254 nm) and recrystallization (CH_2_Cl_2_/hexanes) afforded **19t** as white crystals (115 mg, 35%). Data for **19t**: mp 152.2–153.3 °C; ^1^H NMR (400 MHz, CDCl_3_): δ 8.62 (dd, *J* = 7.1, 1.1, 2H), 8.22 (dd, *J* = 8.2, 0.9, 2H), 7.76 (appr. t, *J* = 7.6, 2H), 7.41–7.38 (m, 2H), 7.29–7.25 (m, 2H), 7.22–7.18 (m, 1H), 5.94–5.90 (m, 1H), 5.01 (d, *J* = 7.8, 2H), 2.34 (d, *J* = 1.4, 3H); ^13^C­{^1^H} NMR (100 MHz, CDCl_3_): δ 164.1, 143.3, 139.0, 133.9, 131.8, 131.3, 128.4, 128.2, 127.2, 127.0, 126.0, 123.0, 122.5, 39.2, 16.4; HRMS (ESI) *m*/*z*: [M + Na]^+^ calcd for C_22_H_17_NO_2_Na 350.1157; found, 350.1157.

#### *N*-(*E*)-α-methylcinnamyl-1,8-naphthalimide (**19u**)

To a stirred mixture of 1,8-naphthalimide (1.07 g, 5.42 mmol, 1.2 equiv), PPh_3_ (1.42 g, 5.41 mmol, 1.2 equiv), and (*E*)-2-methyl-3-phenylprop-2-en-1-ol (673 mg, 4.54 mmol, 1.0 equiv) in THF (18 mL), was added DIAD (1.4 mL, 6.9 mmol, 1.5 equiv) at 0 °C dropwise. The reaction mixture was stirred at rt for 18 h (unoptimized), and brine (50 mL) was added. The organic layer was separated, and the aqueous layer was extracted with EtOAc (50 mL × 3). The combined organic layers were dried over anhydrous MgSO_4_, filtered through a glass frit, and concentrated under reduced pressure. Column chromatography (SiO_2_, ϕ = 3.5 cm, *l* = 10 cm, CH_2_Cl_2_, *R*_*f*_ = 0.5, UV 254 nm) and recrystallization (CH_2_Cl_2_/hexanes) afforded **19u** as white crystals (719 mg, 49%). Data for **19u**: mp 128.4–129.1 °C; ^1^H NMR (400 MHz, CDCl_3_): δ 8.64 (d, *J* = 8.2, 2H), 8.24 (d, *J* = 9.2, 2H), 7.78 (appr. t, *J* = 7.8, 2H), 7.29–7.22 (m, 4H), 7.16 (t, *J* = 7.1, 1H), 6.42 (s, 1H), 4.91 (s, 2H), 1.97 (s, 3H); ^13^C­{^1^H} NMR (100 MHz, CDCl_3_): δ 164.2, 137.6, 134.1, 133.0, 131.7, 131.5, 129.1, 128.3, 128.0, 127.0, 126.3, 125.8, 122.6, 47.1, 16.6; HRMS (ESI) *m*/*z*: [M + Na]^+^ calcd for C_22_H_17_NO_2_Na 350.1157; found, 350.1159.

#### *N*-((*E*)-4-phenylbut-3-en-1-yl)-1,8-naphthalimide (**19v**)

To a stirred mixture of 1,8-naphthalimide (1.25 g, 6.37 mmol, 1.2 equiv), PPh_3_ (1.67 g, 6.37 mmol, 1.2 equiv), and (*E*)-4-phenylbut-3-en-1-ol (787 mg, 5.31 mmol, 1.0 equiv) in THF (16 mL), was added DIAD (1.6 mL, 8.1 mmol, 1.5 equiv) at 0 °C dropwise. The reaction mixture was stirred at rt for 18 h (unoptimized), and brine (50 mL) was added. The organic layer was separated, and the aqueous layer was extracted with EtOAc (50 mL × 3). The combined organic layers were dried over anhydrous MgSO_4_, filtered through a glass frit, and concentrated under reduced pressure. Multiple column chromatography (SiO_2_, ϕ = 3.5 cm, *l* = 12 cm, CH_2_Cl_2_:hexanes = 1:1, *R*_*f*_ = 0.2, UV 254 nm) and recrystallization (CH_2_Cl_2_/hexanes) afforded **19v** as yellow crystals (1.26 g, 72%). Data for **19v**: mp 180.7–181.5 °C; ^1^H NMR (400 MHz, CDCl_3_): δ 8.61 (dd, *J* = 7.3, 0.9, 2H), 8.21 (d, *J* = 8.2, 2H), 7.75 (dd, *J* = 8.2, 7.3, 2H), 7.32 (d, *J* = 6.9, 2H), 7.29–7.25 (m, 2H), 7.18 (appr. t, *J* = 7.1, 1H), 6.47 (d, *J* = 16.0, 1H), 6.30 (dt, *J* = 15.7, 7.1, 1H), 4.34 (t, *J* = 7.6, 2H), 2.70–2.64 (m, 2H); ^13^C­{^1^H} NMR (100 MHz, CDCl_3_): δ 164.2, 137.7, 133.9, 132.3, 131.7, 131.2, 128.5, 128.3, 127.1, 127.1, 127.0, 126.3, 122.9, 40.0, 31.9; HRMS (ESI) *m*/*z*: [M + Na]^+^ calcd for C_22_H_17_NO_2_Na 350.1157; found, 350.1156.

#### *N*-((*E*)-4-phenylbut-3-en-2-yl)-1,8-naphthalimide (**19w**)

To a stirred mixture of 1,8-naphthalimide (1.87 g, 9.48 mmol, 1.2 equiv), PPh_3_ (2.48 g, 9.46 mmol, 1.2 equiv), and (*E*)-4-phenylbut-3-en-2-ol (1.19 g, 8.03 mmol, 1.0 equiv) in DMF (32 mL), was added DIAD (2.3 mL, 12 mmol, 1.5 equiv) at 0 °C dropwise. The reaction mixture was stirred at rt for 40 h (unoptimized), and brine (80 mL) was added. The organic layer was separated, and the aqueous layer was extracted with Et_2_O (80 mL × 3). The combined organic layers were dried over anhydrous MgSO_4_, filtered through a glass frit, and concentrated under reduced pressure. Multiple column chromatography (SiO_2_, ϕ = 4.0 cm, *l* = 13 cm, CH_2_Cl_2_:hexanes = 1:1, *R*_*f*_ = 0.4, UV 254 nm) and recrystallization (CH_2_Cl_2_/hexanes) afforded **19w** as pale yellow crystals (0.71 g, 27%). Data for **19w**: mp 127.3–128.9 °C; ^1^H NMR (400 MHz, CDCl_3_): δ 8.60 (dd, *J* = 7.3, 0.9, 2H), 8.20 (d, *J* = 8.7, 2H), 7.75 (appr. t, *J* = 7.8, 2H), 7.40 (d, *J* = 7.3, 2H), 7.28 (appr. t, *J* = 7.3, 2H), 7.20 (appr. t, *J* = 7.3, 1H), 6.88 (dd, *J* = 16.0, 7.3, 1H), 6.66 (d, *J* = 16.0, 1H), 6.07–6.00 (m, 1H), 1.76 (d, *J* = 6.9, 3H); ^13^C­{^1^H} NMR (100 MHz, CDCl_3_): δ 164.1, 136.9, 133.7, 131.9, 131.4, 131.2, 129.9, 128.5, 128.1, 127.6, 126.9, 126.6, 123.0, 50.8, 18.6; HRMS (ESI) *m*/*z*: [M + Na]^+^ calcd for C_22_H_17_NO_2_Na 350.1157; found, 350.1159.

#### *N*-(*E*)-Cinnamylsuccinimide (**S3**)

To a stirred mixture of succinimide (642 mg, 6.48 mmol, 1.2 equiv), PPh_3_ (1.71 g, 6.50 mmol, 1.2 equiv), and (*E*)-cinnamyl alcohol (724 mg, 5.40 mmol, 1.0 equiv) in THF (21 mL), was added DIAD (1.6 mL, 8.1 mmol, 1.5 equiv) at 0 °C dropwise. The reaction mixture was warmed to rt for 19 h (unoptimized), and brine (50 mL) was added. The organic layer was separated, and the aqueous layer was extracted with EtOAc (50 mL × 3). The combined organic layers were dried over anhydrous MgSO_4_, filtered through a glass frit, and concentrated under reduced pressure. Multiple column chromatography (SiO_2_, ϕ = 4.0 cm, *l* = 11 cm, CH_2_Cl_2_, *R*_*f*_ = 0.3, UV 254 nm) and recrystallization (CH_2_Cl_2_/hexanes) afforded **S3** as white crystals (602 mg, 52%). Data for **S3**:^30^ ^1^H NMR (400 MHz, CDCl_3_): δ 7.35 (d, *J* = 6.9, 2H), 7.30 (appr. t, *J* = 7.3, 2H), 7.26–7.22 (m, 1H), 6.64 (d, *J* = 16.0, 1H), 6.17 (dt, *J* = 15.7, 6.8, 1H), 4.27 (d, *J* = 6.9, 2H), 2.74 (s, 4H).

#### *N*-(*E*)-Cinnamylglutarimide (**S4**)

To a stirred mixture of glutarimide (293 mg, 2.59 mmol, 1.2 equiv), PPh_3_ (687 mg, 2.62 mmol, 1.2 equiv), and (*E*)-cinnamyl alcohol (290 mg, 2.16 mmol, 1.0 equiv) in THF (10 mL), was added DIAD (0.63 mL, 3.20 mmol, 1.5 equiv) at 0 °C dropwise. The reaction mixture was stirred at rt for 14 h (unoptimized), and brine (20 mL) was added. The organic layer was separated, and the aqueous layer was extracted with EtOAc (20 mL × 3). The combined organic layers were dried over anhydrous MgSO_4_, filtered through a glass frit, and concentrated under reduced pressure. Multiple column chromatography (SiO_2_, ϕ = 4.0 cm, *l* = 11 cm, CH_2_Cl_2_, *R*_*f*_ = 0.5, UV 254 nm) afforded **S4** as a white solid (273 mg, 55%). Data for **S4**:^30 1^H NMR (400 MHz, CDCl_3_): δ 7.35 (d, *J* = 8.2, 2H), 7.30–7.26 (m, 2H), 7.21 (appr. t, *J* = 7.8, 1H), 6.62 (d, *J* = 16.2, 1H), 6.23–6.15 (m, 1H), 4.52 (d, *J* = 6.7, 2H), 2.67 (t, *J* = 6.0, 4H), 1.99–1.92 (m, 2H).

### *syn*-Dichlorination of Protected Allylic Alcohols/Amines

#### General Procedure A (GP-A): (COCl)_2_/Ph_2_SO

To a stirred solution of alkene (1.00 mmol) and Ph_2_SO (243 mg, 1.20 mmol, 1.2 equiv) in CH_2_Cl_2_ (10 mL), was added (COCl)_2_ (103 μL, 1.20 mmol, 1.2 equiv) dropwise over 10 min. Then, the reaction mixture was concentrated under reduced pressure. Column chromatography on silica gel afforded the corresponding *syn*-dichlorinated product.

#### General Procedure B (GP-B): SO_2_Cl_2_

To a stirred solution of alkene (1.00 mmol) in CH_2_Cl_2_ (10 mL), was added SO_2_Cl_2_ (97 μL, 1.2 mmol, 1.2 equiv) in one portion. After 30 min, the reaction mixture was concentrated under reduced pressure. Column chromatography on silica gel afforded the corresponding *syn*-dichlorinated product.

#### 2,3-Dichloro-3-phenylpropyl Acetate (**14a**)

From **13a** (176 mg), column chromatography (SiO_2_, ϕ = 3.5 cm, *l* = 11 cm, EtOAc/hexanes = 1/10, *R*_*f*_ = 0.3, UV 254 nm) afforded a mixture of **14a**, **15a**, and chloroalkene **S5** as a pale yellow oil (GP-A: 204 mg, 83%, *syn*-**14a**:*anti*-**14a**:**15a**/**S5** = 73:9:12:6; GP-B: 215 mg, 87%, *syn*-**14a**:*anti*-**14a**:**15a**/**S5** = 77:11:8:4). Data for *syn*-**14a**: ^1^H NMR (400 MHz, CDCl_3,_ diagnostic resonance): δ 5.20 (d, *J* = 5.5, 1H), 4.14 (dd, *J* = 11.7, 6.2, 1H); ^13^C­{^1^H} NMR (100 MHz, CDCl_3_): δ 170.2, 137.1, 129.1, 128.7, 127.7, 65.1, 63.4, 62.7, 20.7; HRMS (ESI) *m*/*z*: [M + Na]^+^ calcd for C_11_H_12_^35^Cl^35^ClO_2_Na, C_11_H_12_^37^Cl^35^ClO_2_Na, C_11_H_12_^37^Cl^37^ClO_2_Na 269.0112 (100.0%), 271.0083 (64.8%), 273.0053 (10.5%); found, 269.0106 (100.0%), 271.0076, (65.9%), 273.0049 (10.9%); Data for *anti*-**14a**: ^1^H NMR (400 MHz, CDCl_3,_ diagnostic resonance): δ 5.06 (d, *J* = 8.7, 1H); Data for **15a**: ^1^H NMR (400 MHz, CDCl_3,_ diagnostic resonance): δ 6.14 (d, *J* = 5.5, 1H), 3.76 (dd, *J* = 11.9, 6.0, 1H), 3.61 (dd, *J* = 11.9, 6.4, 1H); Data for **S5**: ^1^H NMR (400 MHz, CDCl_3,_ diagnostic resonance): δ 7.02 (s, 1H), 4.88 (s, 2H).

#### 2,3-Dichloro-3-phenylpropyl 4-(trifluoromethyl)­benzoate (**14c**)

From **13c** (306 mg), column chromatography (SiO_2_, ϕ = 4.0 cm, *l* = 11 cm, CH_2_Cl_2_/hexanes = 1/4, *R*_*f*_ = 0.2, UV 254 nm) afforded a mixture of **14c**, **15c**, and chloroalkene **S6** as a pale yellow oil (GP-A: 320 mg, 85%, *syn*-**14c**:*anti*-**14c**:**15c**/**S6** = 69:7:15:9; GP-B: 293 mg, 78%, **13c**: *syn*-**14c**:*anti*-**14c**/**S6** = 87:10:2:1). Data for *syn*-**14c**: ^1^H NMR (400 MHz, CDCl_3,_ diagnostic resonance): δ 5.26 (d, *J* = 5.5, 1H), 4.42 (dd, *J* = 11.0, 6.0, 1H); ^13^C­{^1^H} NMR (100 MHz, CDCl_3_): The accurate peak assignment was challenging. A copy of the spectrum is given in the Supporting Information.; ^19^F NMR (376 MHz, CDCl_3_): δ −66.4; HRMS (ESI) *m*/*z*: [M + Na]^+^ calcd for C_17_H_13_^35^Cl^35^ClF_3_O_2_Na, C_17_H_13_^37^Cl^35^ClF_3_O_2_Na, C_17_H_13_^37^Cl^37^ClF_3_O_2_Na 399.0143 (100.0%), 401.0113 (64.8%), 403.0084 (10.5%); found, 399.0141 (100.0%), 401.0111 (64.9%), 403.0087 (10.0%); Data for *anti*-**14c**: ^1^H NMR (400 MHz, CDCl_3,_ diagnostic resonance): δ 5.13 (d, *J* = 8.7, 1H), 4.88 (dd, *J* = 11.9, 3.7, 1H), 4.82 (dd, *J* = 11.9, 5.5, 1H); Data for **15c**: ^1^H NMR (400 MHz, CDCl_3,_ diagnostic resonance): δ 6.45 (d, *J* = 5.0, 1H), 3.82 (dd, *J* = 11.7, 5.7, 1H), 3.63 (dd, *J* = 11.7, 7.1, 1H); Data for **S6**: ^1^H NMR (400 MHz, CDCl_3,_ diagnostic resonance): δ 7.10 (s, 1H), 5.15 (s, 2H).

#### 2,3-Dichloro-3-phenylpropyl 4-Methoxybenzoate (**14d**)

From **13d** (268 mg), column chromatography (SiO_2_, ϕ = 3.0 cm, *l* = 11 cm, CH_2_Cl_2_/hexanes = 1/1, *R*_*f*_ = 0.7, UV 254 nm) afforded a mixture of **14d**, **15d**, and chloroalkene **S7** as a pale yellow oil (GP-A: 325 mg, 96%, *syn*-**14d**:*anti*-**14d**:**15d**/**S7** = 65:5:26:4; GP-B: 342 mg, 101%, *syn*-**14d**:*anti*-**14d**:**15d**/**S7** = 73:8:16:3). Data for *syn*-**14d**: ^1^H NMR (400 MHz, CDCl_3,_ diagnostic resonance): δ 5.28 (d, *J* = 5.0, 1H), 4.36 (dd, *J* = 13.3, 7.8, 1H); ^13^C­{^1^H} NMR (100 MHz, CDCl_3_): The accurate peak assignment was challenging. A copy of the spectrum is given in the Supporting Information.; HRMS (ESI) *m*/*z*: [M + Na]^+^ calcd for C_17_H_16_^35^Cl^35^ClO_3_Na, C_17_H_16_^37^Cl^35^ClO_3_Na, C_17_H_16_^37^Cl^37^ClO_3_Na 361.0374 (100.0%), 363.0345 (64.8%), 365.0315 (10.5%); found, 361.0370 (100.0%), 363.0342 (65.8%), 365.0318 (10.3%); Data for *anti*-**14d**: ^1^H NMR (400 MHz, CDCl_3,_ diagnostic resonance): δ 5.15 (d, *J* = 8.2, 1H); Data for **15d**: ^1^H NMR (400 MHz, CDCl_3,_ diagnostic resonance): δ 6.38 (d, *J* = 5.0, 1H), 3.81 (dd, *J* = 11.9, 6.0, 1H), 3.67 (dd, *J* = 11.7, 6.6, 1H). Data for **S7**: ^1^H NMR (400 MHz, CDCl_3,_ diagnostic resonance): δ 7.06 (s, 1H), 5.08 (s, 2H).

#### 2,3-Dichloro-3-phenylpropyl Diethylcarbamate (**14e**)

From **13e** (233 mg), column chromatography (SiO_2_, ϕ = 3.5 cm, *l* = 11 cm, EtOAc/hexanes = 1/10, *R*_*f*_ = 0.2, CAM) afforded a mixture of **14e**, **15e**, and chloroalkene **S8** as a pale yellow oil (GP-A: 280 mg, 92%, *syn*-**14e**:*anti*-**14e**:**15e**/**S8** = 63:5:30:2; GP-B: 290 mg, 95%, *syn*-**14e**:*anti*-**14e**:**15e**/**S8** = 66:10:22:2). Data for *syn*-**14e**: ^1^H NMR (400 MHz, CDCl_3,_ diagnostic resonance): δ 5.18 (d, *J* = 5.0, 1H), 4.40 (dd, *J* = 11.4, 5.0, 1H), 4.13 (dd, *J* = 11.4, 6.4, 1H); ^13^C­{^1^H} NMR (100 MHz, CDCl_3_): The accurate peak assignment was challenging. A copy of the spectrum is given in the Supporting Information.; HRMS (ESI) *m*/*z*: [M + Na]^+^ calcd for C_14_H_19_^35^Cl^35^ClNO_2_Na, C_14_H_19_^37^Cl^35^ClNO_2_Na, C_14_H_19_^37^Cl^37^ClNO_2_Na 326.0691 (100.0%), 328.0661 (64.8%), 330.0632 (10.5%); found, 326.0688 (100.0%), 328.0659 (64.6%), 330.0632 (9.8%); Data for *anti*-**14e**: ^1^H NMR (400 MHz, CDCl_3,_ diagnostic resonance): 5.03 (d, *J* = 8.2, 1H), 4.61 (dd, *J* = 11.2, 3.0, 1H); Data for **15e**: ^1^H NMR (400 MHz, CDCl_3,_ diagnostic resonance): δ 6.15 (d, *J* = 4.6, 1H), 3.68 (dd, *J* = 11.9, 6.4, 1H), 3.56 (dd, *J* = 11.7, 7.1, 1H); Data for **S8**: ^1^H NMR (400 MHz, CDCl_3,_ diagnostic resonance): δ 6.97 (s, 1H), 4.89 (s, 2H).

#### *N*-(2,3-Dichloro-3-phenylpropan-1-yl)-4-methylphthalimide (**22**)

From **17** (277 mg), column chromatography (SiO_2_, ϕ = 2.5 cm, *l* = 11 cm, CH_2_Cl_2_/hexanes = 1/1, *R*_*f*_ = 0.2, UV 254 nm) afforded a mixture of **22** and chloroalkene **S9** as white solid (GP-A: 326 mg, 94%, *syn*-**22**:*anti*-**22**:**S9** = 82:4:14; GP-B: 339 mg, 97%, *syn*-**22**:*anti*-**22**:**S9** = 85:3:12). Data for *syn*-**22**: ^1^H NMR (400 MHz, CDCl_3_): δ 7.72 (d, *J* = 7.3, 1H), 7.64 (s, 1H), 7.53–7.45 (m, 3H), 7.41–7.28 (m, 3H), 5.14 (d, *J* = 5.5, 1H), 4.84–4.79 (m, 1H), 4.07 (dd, *J* = 14.2, 9.2, 1H), 3.88 (dd, *J* = 14.2, 4.6, 1H), 2.51 (s, 3H); ^13^C­{^1^H} NMR (100 MHz, CDCl_3_): δ 167.8, 167.7, 145.5, 137.1, 134.72, 134.66, 132.0, 129.0, 128.6, 127.7, 124.0, 123.3, 64.3, 62.7, 42.2, 22.0; HRMS (ESI) *m*/*z*: [M + Na]^+^ calcd for C_18_H_15_^35^Cl^35^ClNO_2_Na, C_18_H_15_^37^Cl^35^ClNO_2_Na, C_18_H_15_^37^Cl^37^ClNO_2_Na 370.0378 (100.0%), 372.0348 (64.8%), 374.0319 (10.5%); found, 370.0374 (100.0%), 372.0344 (62.6%), 374.0321 (9.7%); Data for *anti*-**22**: ^1^H NMR (400 MHz, CDCl_3,_ diagnostic resonance): δ 5.05 (d, *J* = 8.7, 1H), 4.33 (dd, *J* = 14.2, 3.7, 1H), 4.15 (dd, *J* = 14.2, 10.1, 1H); Data for **S9**: ^1^H NMR (400 MHz, CDCl_3,_ diagnostic resonance): δ 6.97 (s, 1H), 4.69 (d, *J* = 1.4, 2H).

#### *N*-(2,3-Dichloro-3-phenylpropan-1-yl)-2,3-naphthalimide (**25**)

From **20** (313 mg), column chromatography (SiO_2_, ϕ = 4.0 cm, *l* = 10 cm, CH_2_Cl_2_/hexanes = 1/1, *R*_*f*_ = 0.1, UV 254 nm) afforded a mixture of **25** and chloroalkene **S10** as white solid (GP-A: 354 mg, 92%, *syn*-**25**:**S10** = 90:10; GP-B: 362 mg, 94%, *syn*-**25**:*anti*-**25**:**S10** = 92:3:5). Data for *syn*-**25**: ^1^H NMR (400 MHz, CDCl_3_): δ 8.35 (s, 2H), 8.06 (dd, *J* = 6.2, 3.4, 2H), 7.71 (dd, *J* = 6.2, 3.4, 2H), 7.50 (d, *J* = 7.3, 2H), 7.38 (appr. t, *J* = 7.6, 2H), 7.32 (appr. t, *J* = 7.8, 1H), 5.18 (d, *J* = 5.5, 1H), 4.92–4.87 (m, 1H), 4.17 (dd, *J* = 14.2, 9.6, 1H), 3.96 (dd, *J* = 14.2, 4.6, 1H); ^13^C­{^1^H} NMR (100 MHz, CDCl_3_): δ 167.6, 137.4, 135.7, 130.5, 129.5, 129.2, 128.8, 128.0, 127.6, 125.2, 64.6, 62.6, 42.8; HRMS (ESI) *m*/*z*: [M + Na]^+^ calcd for C_21_H_15_^35^Cl^35^ClNO_2_Na, C_21_H_15_^37^Cl^35^ClNO_2_Na, C_21_H_15_^37^Cl^37^ClNO_2_Na 406.0378 (100.0%), 408.0348 (64.8%), 410.0319 (10.5%); found, 406.0374 (100.0%), 408.0347 (63.8%), 410.0325 (11.1%); Data for *anti*-**25**: ^1^H NMR (400 MHz, CDCl_3,_ diagnostic resonance): δ 5.09 (d, *J* = 8.2, 1H); Data for **S10**: ^1^H NMR (400 MHz, CDCl_3,_ diagnostic resonance): δ 7.00 (s, 1H), 4.79 (d, *J* = 1.4, 2H).

#### *N*-(2,3-Dichloro-3-Cyclohexylpropan-1-yl)-1,8-naphthalimide (**24p**)

From **19p** (319 mg), column chromatography (SiO_2_, ϕ = 4.0 cm, *l* = 11 cm, CH_2_Cl_2_/hexanes = 1/1, *R*_*f*_ = 0.4, UV 254 nm) afforded **24p** as white solid (GP-A: 150 mg, 38%, *syn*-**24p**:*anti*-**24p** = 88:12; GP-B: 138 mg, 35%, *syn*-**24p**:*anti*-**24p** = 80:20). Data for *syn*-**24p**: ^1^H NMR (400 MHz, CDCl_3_): δ 8.62 (d, *J* = 7.3, 2H), 8.23 (d, *J* = 8.2, 2H), 7.77 (appr. t, *J* = 7.8, 2H), 5.11 (dd, *J* = 13.7, 9.2, 1H), 4.86 (appr. td, *J* = 5.7, 3.5, 1H), 4.23 (dd, *J* = 13.7, 3.7, 1H), 3.96 (dd, *J* = 8.9, 2.1, 1H), 2.28 (d, *J* = 12.8, 1H), 1.90–1.65 (m, 5H), 1.35–1.00 (m, 5H); ^13^C­{^1^H} NMR (100 MHz, CDCl_3_): δ 164.3, 134.4, 131.7, 131.6, 128.3, 127.1, 122.3, 69.5, 59.7, 45.5, 42.3, 30.6, 29.8, 26.2, 25.82, 25.81; HRMS (ESI) *m*/*z*: [M + Na]^+^ calcd for C_21_H_21_^35^Cl^35^ClNO_2_Na, C_21_H_21_^37^Cl^35^ClNO_2_Na, C_21_H_21_^37^Cl^37^ClNO_2_Na 412.0847 (100.0%), 414.0818 (64.8%), 416.0788 (10.5%); found, 412.0839 (100.0%), 414.0813 (62.8%), 416.0791 (10.2%); Data for *anti*-**24p**: ^1^H NMR (400 MHz, CDCl_3,_ diagnostic resonance): δ 4.95 (dd, *J* = 13.3, 10.5, 1H), 4.55 (dd, *J* = 13.3, 3.2, 1H), 4.05 (dd, *J* = 7.8, 4.6, 1H).

#### *N*-(2,3-Dichloro-3-phenylbutan-1-yl)-1,8-naphthalimide (24t)

From **19t** (327 mg), column chromatography (SiO_2_, ϕ = 3.5 cm, *l* = 10 cm, CH_2_Cl_2_/hexanes = 1/1, *R*_*f*_ = 0.5, UV 254 nm) and recrystallization (CH_2_Cl_2_/hexanes) afforded a mixture of **24t**, chloroalkene **S11**, and 1,3-dichloroalkene **S12** as white crystals (GP-A: 202 mg, 51%, *syn*-**24t**/**S11**:**S12** = 63:35:2; GP-B: 180 mg, 45%, *syn*-**24t**/**S11**:**S12** = 49:41:10). Data for *syn*-**24t**: ^1^H NMR (400 MHz, CDCl_3,_ diagnostic resonance): δ 5.24 (dd, *J* = 10.3, 3.9, 1H), 4.72 (dd, *J* = 13.7, 10.1, 1H), 4.19 (dd, *J* = 13.7, 4.1, 1H), 2.27 (s, 3H); ^13^C­{^1^H} NMR (100 MHz, CDCl_3_): The accurate peak assignment was challenging. A copy of the spectrum is given in the Supporting Information.; HRMS (ESI) *m*/*z*: [M + Na]^+^ calcd for C_22_H_17_^35^Cl^35^ClNO_2_Na, C_22_H_17_^37^Cl^35^ClNO_2_Na, C_22_H_17_^37^Cl^37^ClNO_2_Na 420.0534 (100.0%), 422.0505 (64.8%), 424.0475 (10.5%); found, 420.0530 (100.0%), 422.0503 (59.8%), 424.0482 (9.3%); Data for **S11**: ^1^H NMR (400 MHz, CDCl_3,_ diagnostic resonance): δ 5.00 (appr. d, *J* = 1.4, 2H), 2.20 (t, *J* = 1.4, 3H); HRMS (ESI) *m*/*z*: [M + Na]^+^ calcd for C_22_H_16_^35^ClNO_2_Na 384.0767; found, 384.0764; Data for **S12**: ^1^H NMR (400 MHz, CDCl_3,_ diagnostic resonance): δ 5.00 (appr. d, *J* = 1.4, 2H), 4.52 (s, 2H); HRMS (ESI) *m*/*z*: [M + Na]^+^ calcd for C_22_H_15_^35^Cl^35^ClNO_2_Na 418.0378; found, 418.0372.

#### *N*-(2,3-Dichloro-2-methyl-3-phenylpropan-1-yl)-1,8-naphthalimide (**24u**)

From **19u** (327 mg), column chromatography (SiO_2_, ϕ = 4.0 cm, *l* = 11 cm, CH_2_Cl_2_/hexanes = 3/2, *R*_*f*_ = 0.5, UV 254 nm) and recrystallization (CH_2_Cl_2_/hexanes) afforded a mixture of **24u** and chloroalkene **S13** as white crystals (GP-A: 205 mg, 51%, *syn*-**24u**/**S13** = 96:4; GP-B: 159 mg, 40%, *syn*-**24u**). Data for *syn*-**24u**: mp 178.5–179.1 °C; ^1^H NMR (400 MHz, (CD_3_)_2_CO): δ 8.58 (d, *J* = 6.4, 2H), 8.46 (d, *J* = 7.8, 2H), 7.90 (appr. t, *J* = 7.8, 2H), 7.68–7.66 (m, 2H), 7.43–7.39 (m, 3H), 5.60 (s, 1H), 5.06 (d, *J* = 13.7, 1H), 4.75 (d, *J* = 13.7, 1H), 1.50 (s, 3H); ^13^C­{^1^H} NMR (100 MHz, (CD_3_)_2_SO): δ 164.1, 137.2, 134.4, 131.3, 131.0, 129.3, 128.8, 127.9, 127.4, 127.3, 122.2, 75.5, 68.6, 46.9, 27.0; HRMS (ESI) *m*/*z*: [M + Na]^+^ calcd for C_22_H_17_^35^Cl^35^ClNO_2_Na, C_22_H_17_^37^Cl^35^ClNO_2_Na, C_22_H_17_^37^Cl^37^ClNO_2_Na 420.0534 (100.0%), 422.0505 (64.8%), 424.0475 (10.5%); found, 420.0530 (100.0%), 422.0502 (59.5%), 424.0480 (9.6%); Data for **S13**: ^1^H NMR (400 MHz, (CD_3_)_2_CO, diagnostic resonance): δ 4.83 (s, 2H), 1.90 (s, 3H).

#### *N*-(3,4-Dichloro-4-phenylbutan-1-yl)-1,8-naphthalimide (**24v**)

From **19v** (327 mg), column chromatography (SiO_2_, ϕ = 3.5 cm, *l* = 11 cm, CH_2_Cl_2_/hexanes = 1/1, *R*_*f*_ = 0.3, UV 254 nm) and recrystallization (CH_2_Cl_2_/hexanes) afforded **24v** as yellow crystals (GP-A: 310 mg, 77%, *syn*-**24v**:*anti*-**24v** = 47:53; GP-B: 329 mg, 63%, *syn*-**24v**:*anti*-**24v** = 46:54). Data for *syn*-**24v**: ^1^H NMR (400 MHz, CDCl_3,_ diagnostic resonance): δ 5.23 (d, *J* = 5.0, 1H), 2.40–2.33 (m, 1H), 2.17–2.07 (m, 1H); ^13^C­{^1^H} NMR (100 MHz, CDCl_3_): The accurate peak assignment was challenging. A copy of the spectrum is given in the Supporting Information.; HRMS (ESI) *m*/*z*: [M + Na]^+^ calcd for C_22_H_17_^35^Cl^35^ClNO_2_Na, C_22_H_17_^37^Cl^35^ClNO_2_Na, C_22_H_17_^37^Cl^37^ClNO_2_Na 420.0534 (100.0%), 422.0505 (64.8%), 424.0475 (10.5%); found, 420.0526 (100.0%), 422.0499 (60.6%), 424.0477 (10.3%); Data for *anti*-**24v**: ^1^H NMR (400 MHz, CDCl_3,_ diagnostic resonance): δ 5.00 (d, *J* = 8.2, 1H), 2.68–2.60 (m, 1H), 2.30–2.21 (m, 1H).

#### *N*-(3,4-Dichloro-4-phenylbutan-2-yl)-1,8-naphthalimide (**24w**)

From **19w** (327 mg), column chromatography (SiO_2_, ϕ = 4.0 cm, *l* = 10 cm, CH_2_Cl_2_/hexanes = 1/1, *R*_*f*_ = 0.4, UV 254 nm) and recrystallization (CH_2_Cl_2_/hexanes) afforded an isomeric mixture of **24w** as white crystals (GP-A: 86 mg, 22%, *syn*,*anti*-**24w**: *syn*,*syn*-**24w** = 97:3). Data for *syn*,*anti*-**24w**: ^1^H NMR (400 MHz, CDCl_3_): δ 8.67 (d, *J* = 7.3, 2H), 8.26 (d, *J* = 8.2, 2H), 7.80 (appr. t, *J* = 7.8, 2H), 7.42 (d, *J* = 7.3, 2H), 7.32–7.22 (m, 3H), 5.67–5.59 (m, 1H), 5.35 (dd, *J* = 9.8, 2.1, 1H), 5.15 (d, *J* = 1.8, 1H), 1.72 (d, *J* = 6.4, 3H); ^13^C­{^1^H} NMR (100 MHz, CDCl_3_): δ 164.6, 138.3, 134.4, 131.9, 131.7, 128.4, 128.3, 128.2, 127.5, 127.2, 122.6, 68.8, 62.8, 53.0, 16.0; HRMS (ESI) *m*/*z*: [M + Na]^+^ calcd for C_22_H_17_^35^Cl^35^ClNO_2_Na, C_22_H_17_^37^Cl^35^ClNO_2_Na, C_22_H_17_^37^Cl^37^ClNO_2_Na 420.0534 (100.0%), 422.0505 (64.8%), 424.0475 (10.5%); found, 420.0526 (100.0%), 422.0498 (60.2%), 424.0478 (9.7%); Data for *syn*,*syn*-**24w**: ^1^H NMR (400 MHz, CDCl_3,_ diagnostic resonance): δ 5.94–5.86 (m, 1H), 5.52 (d, *J* = 0.9, 1H). The *syn*,*anti*-diastereomer was crystallized out, and its relative configuration was determined by X-ray diffraction analysis. Then, the remaining diastereomer was assumed to be *syn*,*syn*.

### *syn*-Dibromination of Protected Allylic Alcohol/Amines

#### General Procedure C (GP-C): Br_2_

To a stirred solution of alkene (1.00 mmol) in CH_2_Cl_2_ (9 mL), was added a solution of Br_2_ (51 μL, 1.0 mmol, 1.0 equiv) in CH_2_Cl_2_ (1 mL) dropwise over 10 min. After 0.5–1 h, the reaction mixture was concentrated under reduced pressure. Column chromatography on silica gel afforded the corresponding *syn*-dibrominated product.

#### 2,3-Dibromo-3-phenylpropyl benzoate (**28**)

From **13b** (238 mg), column chromatography (SiO_2_, ϕ = 4.0 cm, *l* = 6.0 cm, CH_2_Cl_2_/hexanes = 1/2, *R*_*f*_ = 0.3, UV 254 nm) afforded **28** as white solid (376 mg, 94%, *syn*-**28**:*anti*-**28** = 57:43). Data for *syn*-**28**: ^1^H NMR (400 MHz, CDCl_3_, diagnostic resonance): δ 5.39 (d, *J* = 5.0, 1H), 4.73–4.68 (m, 2H), 4.50–4.45 (m, 1H); ^13^C­{^1^H} NMR (100 MHz, CDCl_3_): The accurate peak assignment was challenging. A copy of the spectrum is given in the Supporting Information.; HRMS (ESI) *m*/*z*: [M + Na]^+^ calcd for C_16_H_14_^79^Br^79^BrO_2_Na, C_16_H_14_^81^Br^79^BrO_2_Na, C_16_H_14_^81^Br^81^BrO_2_Na 418.9258 (51.3%), 420.9238 (100.0%), 422.9217 (48.8%); found, 418.9255 (49.0%), 420.9236 (100.0%), 422.9216 (47.1%); Data for *anti*-**28**: ^1^H NMR (400 MHz, CDCl_3_, diagnostic resonance): δ 5.28 (d, *J* = 10.5, 1H), 5.03 (dd, *J* = 12.4, 3.2, 1H), 4.96 (dd, *J* = 12.4, 5.0, 1H), 4.84 (ddd, *J* = 10.4, 5.2, 3.3, 1H).

#### *N*-(2,3-Dibromo-3-phenylpropan-1-yl)-phthalimide (**29**)

From **16** (262 mg), column chromatography (SiO_2_, ϕ = 3.5 cm, *l* = 8.0 cm, CH_2_Cl_2_, *R*_*f*_ = 0.7, UV 254 nm) afforded **29** as white solid (393 mg, 93%, *syn*-**29**:*anti*-**29** = 34:66). Data for *syn*-**29**: ^1^H NMR (400 MHz, CDCl_3_, diagnostic resonance): δ 5.27 (d, *J* = 6.4, 1H), 5.00 (ddd, *J* = 10.1, 5.5, 4.1, 1H), 4.14 (dd, *J* = 14.2, 9.6, 1H), 4.04 (dd, *J* = 14.2, 4.6, 1H); Data for *anti*-**29**: ^1^H NMR (400 MHz, CDCl_3_, diagnostic resonance): δ 5.17 (d, *J* = 10.5, 1H), 5.09 (appr. td, *J* = 10.2, 3.5, 1H), 4.61 (dd, *J* = 14.2, 3.7, 1H), 4.26 (dd, *J* = 14.2, 10.1, 1H). ^13^C­{^1^H} NMR (100 MHz, CDCl_3_): The accurate peak assignment was challenging. A copy of the spectrum is given in the Supporting Information.; HRMS (ESI) *m*/*z*: [M + Na]^+^ calcd for C_17_H_13_^79^Br^79^BrNO_2_Na, C_17_H_13_^81^Br^79^BrNO_2_Na, C_17_H_13_^81^Br^81^BrNO_2_Na 443.9211 (51.3%), 445.9190 (100.0%), 447.9170 (48.8%); found, 443.9203 (48.7%), 445.9184 (100.0%), 447.9164 (50.0%).

#### *N*-(2,3-Dibromo-3-phenylpropan-1-yl)-1,8-naphthalimide (**30**)

From **19a** (313 mg), column chromatography (SiO_2_, ϕ = 4.0 cm, *l* = 7.0 cm, CH_2_Cl_2_, *R*_*f*_ = 0.6, UV 254 nm) afforded **30** as white solid (428 mg, 91%, **19a**:*syn*-**30**:*anti*-**30** = 8:87:5). Data for *syn*-**30**: ^1^H NMR (400 MHz, (CD_3_)_2_CO): δ 8.55 (d, *J* = 7.3, 2H), 8.44 (d, *J* = 8.2, 2H), 7.87 (appr. t, *J* = 7.8, 2H), 7.65 (d, *J* = 9.2, 2H), 7.36 (appr. t, *J* = 6.6, 2H), 7.28 (appr. t, *J* = 7.3, 1H), 5.72 (d, *J* = 6.0, 1H), 5.21–5.16 (m, 1H), 4.84 (dd, *J* = 13.7, 9.2, 1H), 4.39 (dd, *J* = 13.7, 5.0, 1H); ^13^C­{^1^H} NMR (100 MHz, (CD_3_)_2_SO): δ 163.4, 138.6, 134.6, 131.2, 131.0, 128.7, 128.4, 128.3, 127.4, 127.2, 121.7, 56.9, 56.8, 44.2; HRMS (ESI) *m*/*z*: [M + Na]^+^ calcd for C_21_H_15_^79^Br^79^BrNO_2_Na, C_21_H_15_^81^Br^79^BrNO_2_Na, C_21_H_15_^81^Br^81^BrNO_2_Na 493.9367 (51.3%), 495.9347 (100.0%), 497.9326 (48.8%); found, 493.9357 (49.1%), 495.9339 (100.0%), 497.9319 (49.4%); Data for *anti*-**30**: ^1^H NMR (400 MHz, (CD_3_)_2_CO, diagnostic resonance): δ 5.64 (d, *J* = 10.1, 1H), 5.39 (appr. td, *J* = 10.2, 4.0, 1H), 4.99 (dd, *J* = 13.7, 10.5, 1H).

#### *N*-(2,3-Dibromo-3-phenylpropan-1-yl)-succinimide (**31**)

From **S3** (215 mg), column chromatography (SiO_2_, ϕ = 3.0 cm, *l* = 11 cm, CH_2_Cl_2_, *R*_*f*_ = 0.2, UV 254 nm) afforded **31** as pale yellow solid (298 mg, 79%, *syn*-**31**:*anti*-**31** = 52:48). Data for *syn*-**31**: ^1^H NMR (400 MHz, CDCl_3_, diagnostic resonance): δ 5.20 (d, *J* = 6.9, 1H), 4.99–4.93 (m, 1H), 3.99 (dd, *J* = 14.2, 10.1, 1H), 3.79 (dd, *J* = 14.2, 4.6, 1H), 2.66 (s, 4H); ^13^C­{^1^H} NMR (100 MHz, CDCl_3_): The accurate peak assignment was challenging. A copy of the spectrum is given in the Supporting Information.; HRMS (ESI) *m*/*z*: [M + Na]^+^ calcd for C_13_H_13_^79^Br^79^BrNO_2_Na, C_13_H_13_^81^Br^79^BrNO_2_Na, C_13_H_13_^81^Br^81^BrNO_2_Na 395.9211 (51.3%), 397.9190 (100.0%), 399.9170 (48.8%); found, 395.9202 (51.0%), 397.9183 (100.0%), 399.9162 (47.5%); Data for *anti*-**31**: ^1^H NMR (400 MHz, CDCl_3_, diagnostic resonance): δ 5.10 (d, *J* = 10.5, 1H), 5.03 (appr. td, *J* = 10.3, 3.7, 1H), 4.39 (dd, *J* = 13.7, 3.7, 1H), 4.10 (dd, *J* = 14.2, 10.1, 1H), 2.77 (s, 4H).

#### *N*-(2,3-Dibromo-3-phenylpropan-1-yl)-glutarimide (**32**)

From **S4** (229 mg), multiple column chromatography (SiO_2_, ϕ = 4.0 cm, *l* = 10 cm, CH_2_Cl_2_, *R*_*f*_ = 0.5, UV 254 nm) afforded **32** as a colorless oil (266 mg, 68%, *syn*-**32**:*anti*-**32** = 85:15). Data for *syn*-**32**: ^1^H NMR (400 MHz, CDCl_3_, diagnostic resonance): δ 5.19 (d, *J* = 7.3, 1H), 4.96–4.90 (m, 1H), 4.42 (dd, *J* = 13.7, 10.1, 1H), 3.83 (dd, *J* = 14.0, 4.4, 1H), 2.60 (t, *J* = 6.6, 4H), 1.92–1.86 (m, 2H); ^13^C­{^1^H} NMR (100 MHz, CDCl_3_): δ 172.4, 138.2, 129.1, 128.8, 128.3, 56.7, 55.8, 43.5, 32.8, 16.9; HRMS (ESI) *m*/*z*: [M + Na]^+^ calcd for C_14_H_15_^79^Br^79^BrNO_2_Na, C_14_H_15_^81^Br^79^BrNO_2_Na, C_14_H_15_^81^Br^81^BrNO_2_Na 409.9367 (51.3%), 411.9347 (100.0%), 413.9326 (48.8%); found, 409.9354 (48.5%), 411.9334 (100.0%), 413.9314 (47.7%); Data for *anti*-**32**: ^1^H NMR (400 MHz, CDCl_3_, diagnostic resonance): 5.11 (d, *J* = 10.5, 1H), 5.03 (appr. td, *J* = 10.3, 4.1, 1H), 4.56 (dd, *J* = 13.7, 10.1, 1H), 2.71 (t, *J* = 6.4, 4H), 2.01–1.95 (m, 2H).

### Other Halogens - Chloroiodination

#### *N*-(3-Chloro-2-iodo-3-phenylpropan-1-yl)-1,8-naphthalimide (**33**)

To a stirred solution of **19a** (313 mg, 1.00 mmol, 1.0 equiv) in CH_2_Cl_2_ (8.8 mL), was added a solution of ICl (195 mg, 1.20 mmol, 1.2 equiv) in CH_2_Cl_2_ (1.2 mL) in one portion. After 0.5 h, the reaction mixture was concentrated under reduced pressure. Column chromatography (SiO_2_, ϕ = 3.0 cm, *l* = 6.0 cm, CH_2_Cl_2_/hexanes = 1/1, *R*_*f*_ = 0.3, UV 254 nm, CAM) afforded *anti*-**33** as white solid (424 mg, 89%, >50:1 dr). Data for *anti*-**33**: ^1^H NMR (400 MHz, CDCl_3_): δ 8.64 (d, *J* = 7.3, 2H), 8.25 (d, *J* = 8.2, 2H), 7.78 (appr. t, *J* = 7.8, 2H), 7.45–7.30 (m, 5H), 5.36 (appr. td, *J* = 10.0, 4.3, 1H), 5.28 (d, *J* = 10.1, 1H), 5.02 (dd, *J* = 13.7, 10.1, 1H), 4.65 (dd, *J* = 14.0, 4.8, 1H); ^13^C­{^1^H} NMR (100 MHz, CDCl_3_): δ 164.2, 140.8, 134.4, 131.8, 131.7, 129.1, 128.7, 128.3, 127.6, 127.1, 122.4, 65.8, 45.6, 34.8; HRMS (ESI) *m*/*z*: [M + Na]^+^ calcd for C_21_H_15_^35^ClINO_2_Na, C_21_H_15_^37^ClINO_2_Na 497.9734 (100.0%), 499.9704 (32.4%); found, 497.9726 (100.0%), 499.9700 (31.0%).

## Supplementary Material





## Data Availability

The data underlying this study are available in the published article and its Supporting Information.

## Notes

Computation output files (ZIP)
Reagent purification methods, X-ray crystallographic data, DFT calculation details, and copies of NMR spectra for all new compounds (PDF)
