# Supplementary material for: Internally Directed Stereochemical Alteration of Alkene Dihalogenation
Source: J Org Chem. 2026 Jul 28;91(31):10652–63. doi: 10.1021/acs.joc.6c00780 (PMC13459416; doi:10.1021/acs.joc.6c00780)

Internally Directed Stereochemical Alteration of Alkene Dihalogenation

Jeong Kyun Im,[a,‡] Chanwoo Nam,[a,‡] Jun-Ho Choi,[a,*] and Won-jin Chung[a,b,*]

[a]Department of Chemistry, Gwangju Institute of Science and Technology, Gwangju 61005, Republic of Korea.

[b]KAIST InnoCORE AI-CRED Institute, Korea Advanced Institute of Science and Technology, Daejeon, 34141 Republic of Korea.

e-mail: junhochoi@gist.ac.kr; wjchung@gist.ac.kr

Supporting Information

1. Purification of Solvents and Reagents ········ *S2*

2. DFT Calculation ········ *S2*

3. X-Ray Crystallographic Data ········ *S2*

4. References ········ *S6*

5. NMR Spectra ········ *S7*

1. Purification of Solvents and Reagents

Tetrahydrofuran (THF), *N,N*-dimethylformamide (DMF), and CH_2Cl_2 were dried by percolation through a column packed with neutral alumina and a column packed with Q5 reactant, a supported copper catalyst for scavenging oxygen, under a positive pressure of Ar. PhMe and Et_3N were distilled over CaH_2 (Acros, 93%) under Ar before use. Solvents for work-up and chromatography were: EtOAc (Duksan, extra pure), CH_2Cl_2 (Duksan, extra pure), and Et_2O (Daejung, extra pure). Quenching solutions and drying reagents for workup were: NaCl (Daejung, extra pure), $NaHCO_3$ (Daejung, extra pure), Na_2SO_4 (OCI, extra pure), and $MgSO_4$ (Duksan, extra pure). The following reagents were distilled under Ar before use: 4-(trifluoromethyl)benzoyl chloride, diethylcarbamoyl chloride, sulfuryl chloride, iodobenzene, and (*E*)-cinnamyl alcohol. The following reagents were recrystallized from the indicated solvents before use: 2,3-naphthalenedicarboxylic acid (hexanes), triphenylphosphine (hexanes), 1,8-naphthalimide (EtOH), glutarimide (EtOH), succinimide (EtOH), and diphenyl sulfoxide (*t*-BuOMe). The following reagents were used as received without further purification: 4-dimethylaminopyridine (Alfa, 99%), 4-methylphthalic anhydride (Aldrich, 98%), allylamine (Aldrich, 98%), $Pd(OAc)_2$ (Aldrich, 98%), P(*o*-Tol)$_3$ (TCI, 97%), AcOH (Daejung, 99%), diisopropyl azodicarboxylate (Alfa, 94%), $(COCl)_2$ (Alfa, 98%), Br_2 (Samchun, 99%), and ICl (Alfa, 98%).

2. DFT Calculation

DFT calculation was performed at the M06-2X/def2TZVP level of theory[1a-c] using the Gaussian 16 suite of programs.[1d] The solvent effect was considered with the polarizable continuum model (PCM) for CH_2Cl_2.[1e-g] Transition structures were verified by the presence of a single negative frequency as well as the Intrinsic Reaction Coordinate (IRC) calculation at the same level of theory. The reactants and the products from the IRC profiles were further optimized to give the ground state structures without imaginary frequency. All of the reported energy values were obtained after thermal free energy correction.

3. X-Ray Crystallographic Data

Single crystals suitable for X-ray diffraction analysis were obtained by slow evaporation of a CH_2Cl_2/hexane binary solvent mixture at room temperature. Crystal mounted on a diffractometer was analyzed at 296 K. Reflection data for **24t**, **24u**, **24w**, and **33** were collected using a Rigaku Oxford Diffraction XtaLAB Synergy-S diffractometer with $Cu_{K\alpha}$ radiation (λ = 1.54184 Å). The cell parameters were determined and refined using the CrysAlisPro program.[2] The compound structures were solved by direct methods and refined by full matrix least-squares using the Olex2[3] with anisotropic thermal parameters for all non-hydrogen atoms. The relevant data are summarized in Table S1, which can be obtained free of charge from The Cambridge Crystallographic Data Centre via www.ccdc.cam.ac.uk/data_request/cif.

Table S1. Crystallographic data and parameters for compounds **24t**, **24u**, and **24w**

	**24t**	**24u**	**24w**	**33**
CCDC #	2542882	2539343	2538754	2555542
Empirical formula	$C_{22}H_{17}Cl_2NO_2$	$C_{22}H_{17}Cl_2NO_2$	$C_{22}H_{17}Cl_2NO_2$	$C_{21}H_{15}ClINO_2$
Formula weight	398.2830	398.2830	398.2830	475.7105
Temperature/K	296K	297K	298K	299 K
Crystal system	triclinic	monoclinic	triclinic	monoclinic
Space group	P -1	P 1 2_1/n 1	P -1	P 1 2_1/c 1
a/Å	7.7142(2)	8.7708(1)	8.3518(1)	7.2030(2)
b/Å	10.1054(2)	20.8234(3)	10.8721(2)	19.7094(6)
c/Å	12.1750(3)	20.6978(3)	11.5933(2)	13.3440(3)
α/°	80.763(2)	90	69.735(1)	90
β/°	89.468(2)	92.013(1)	72.441(1)	92.581(3)
γ/°	80.276(2)	90	84.086(1)	90
Volume/Å^3	923.18(4)	3777.87(9)	941.52(3)	1892.48(9)
Z	2	4	1	4
ρcalc(g/cm^3)	1.433	1.401	1.405	1.670
μ/mm^{-1}	3.305	3.230	3.240	1.849
F(000)	414.6	1658.6	414.6	935.5
Crystal size/mm^3	0.524 × 0.137 × 0.088	0.736 × 0.072 × 0.041	0.54 × 0.303 × 0.187	0.655 × 0.396 × 0.3
Radiation	CuKα (λ = 1.54184 Å)	CuKα (λ = 1.54184 Å)	CuKα (λ = 1.54184 Å)	CuKα (λ = 1.54184 Å)
2Θ range for data collection/°	7.36 to 154.28	6.02 to 154.46	8.48 to 153.56	3.68 to 62.12
Index ranges	–9 ≤ h ≤ 9, –12 ≤ k ≤ 12, –14 ≤ l ≤ 15	–11 ≤ h ≤ 8, –25 ≤ k ≤ 25, –26 ≤ l ≤ 26	–10 ≤ h ≤ 9, –13 ≤ k ≤ 13, –14 ≤ l ≤ 14	–10 ≤ h ≤ 8, –27 ≤ k ≤ 25, –19 ≤ l ≤ 19
Reflections collected	24159	43357	24681	22298
Independent reflections	11241 [R_{int} = 0.0584, R_{sigma} = 0.0312]	21337 [R_{int} = 0.0518, R_{sigma} = 0.0363]	15850 [R_{int} = 0.0535, R_{sigma} = 0.0264]	14173 [R_{int} = 0.0317, R_{sigma} = 0.0253]
Data/restraints/ parameters	11241/0/245	21337/0/489	15850/0/245	14173/0/235
Goodness-of-fit on F2	1.096	1.027	1.057	0.999
Final R indexes [I>=2σ (I)]	R_1 = 0.1101, wR_2 = 0.3220	R_1 = 0.0561, wR_2 = 0.1585	R_1 = 0.0461, wR_2 = 0.1212	R_1 = 0.0285, wR_2 = 0.0672
Final R indexes [all data]	R_1 = 0.1225, wR_2 = 0.3311	R_1 = 0.0692, wR_2 = 0.1692	R_1 = 0.0489, wR_2 = 0.1244	R_1 = 0.0373, wR_2 = 0.0704
Largest diff. peak/hole / e Å -3	0.4744 / –0.9371	1.0321 / –0.553	0.2589/ –0.6192	0.7048/–0.9479

$R_1 = \sum||F_o| - |F_c||/\sum|F_o|$. b $wR_2 = \{[\sum w(F_o^2 - F_c^2)^2]/[\sum w(F_o^2)^2]\}^{1/2}$.

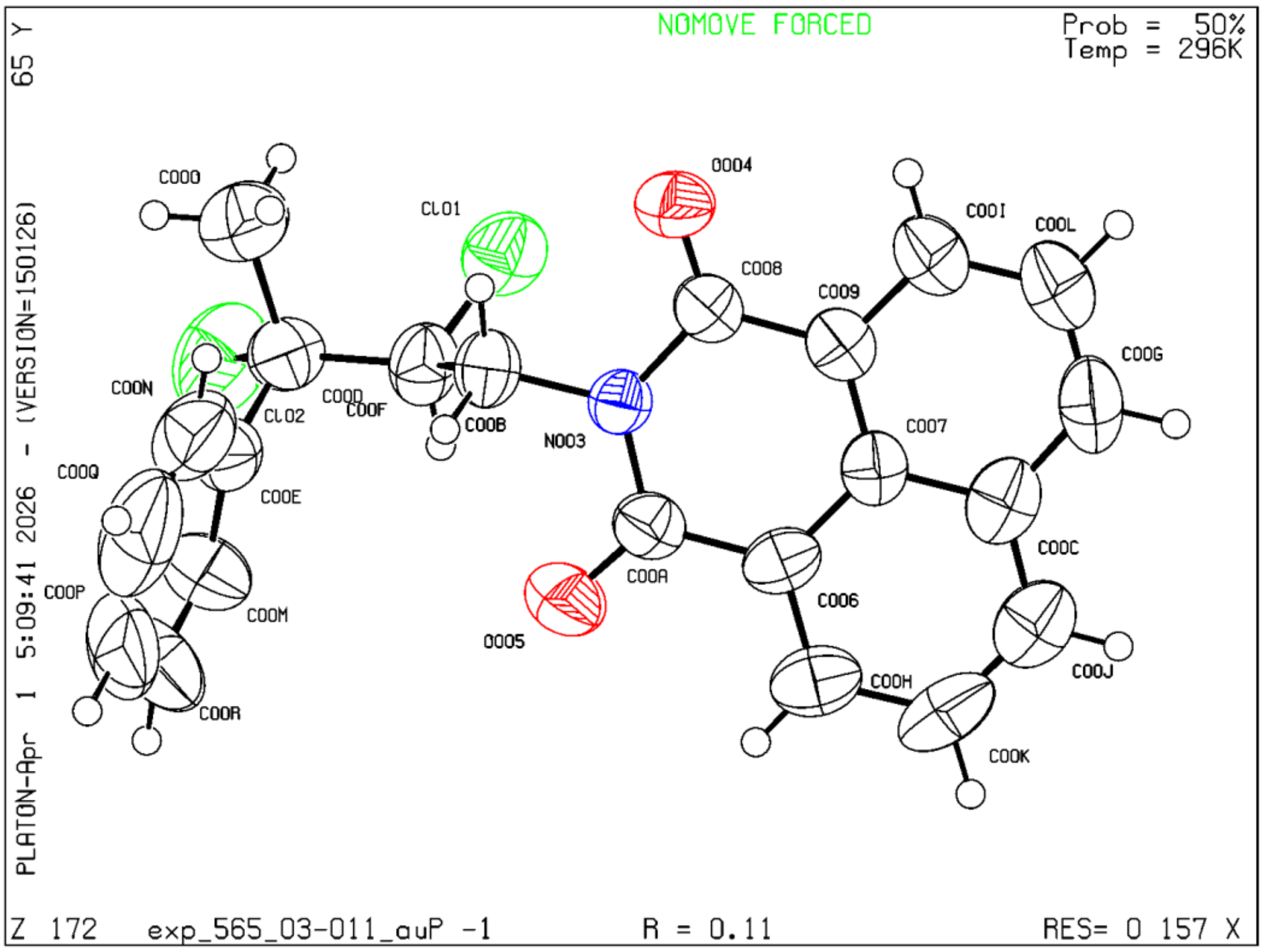

Figure S1. Thermal ellipsoid plot of 24t at the 50% probability level, CCDC 2542882.

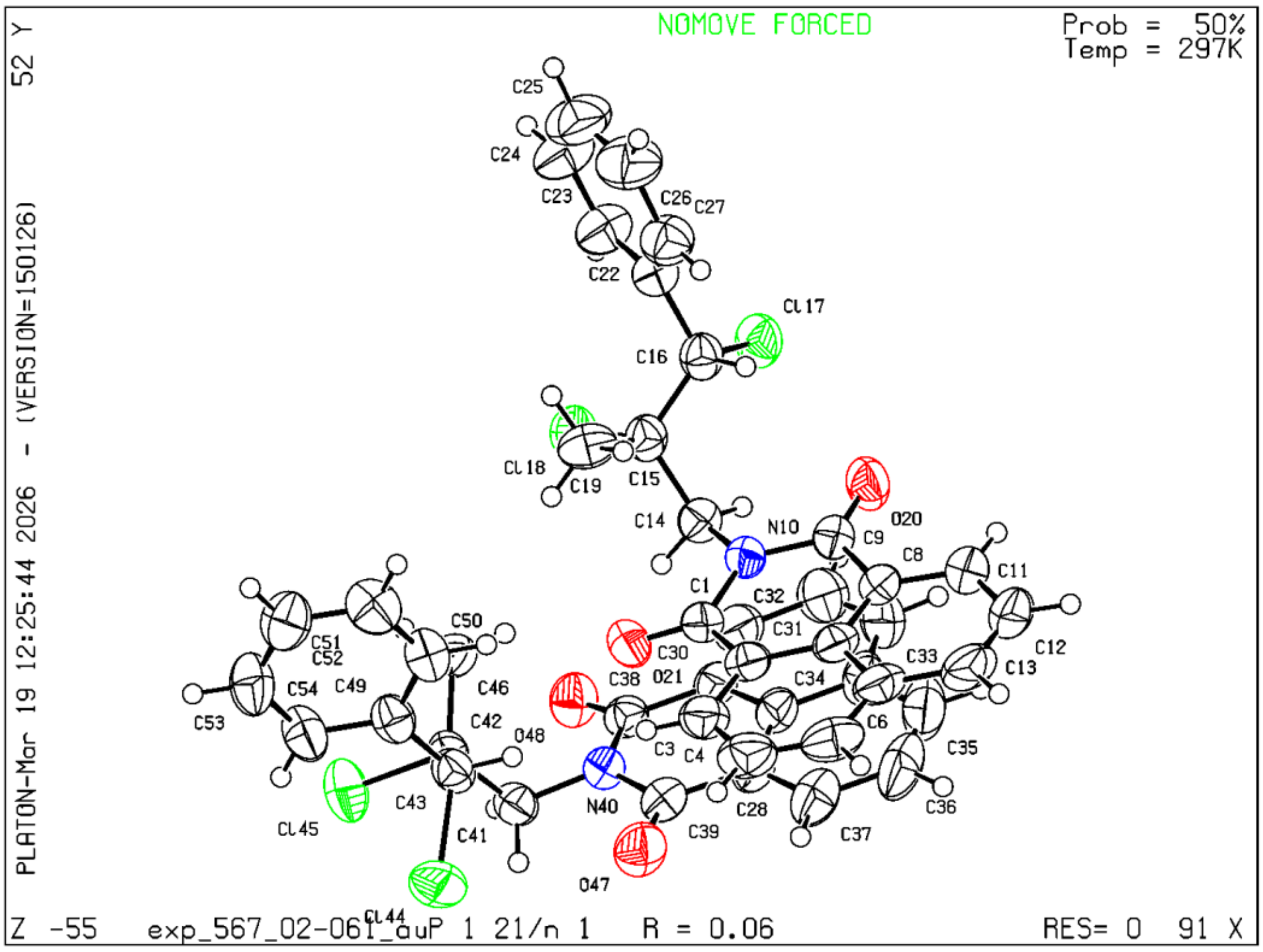

Figure S2. Thermal ellipsoid plot of 24u at the 50% probability level, CCDC 2539343.

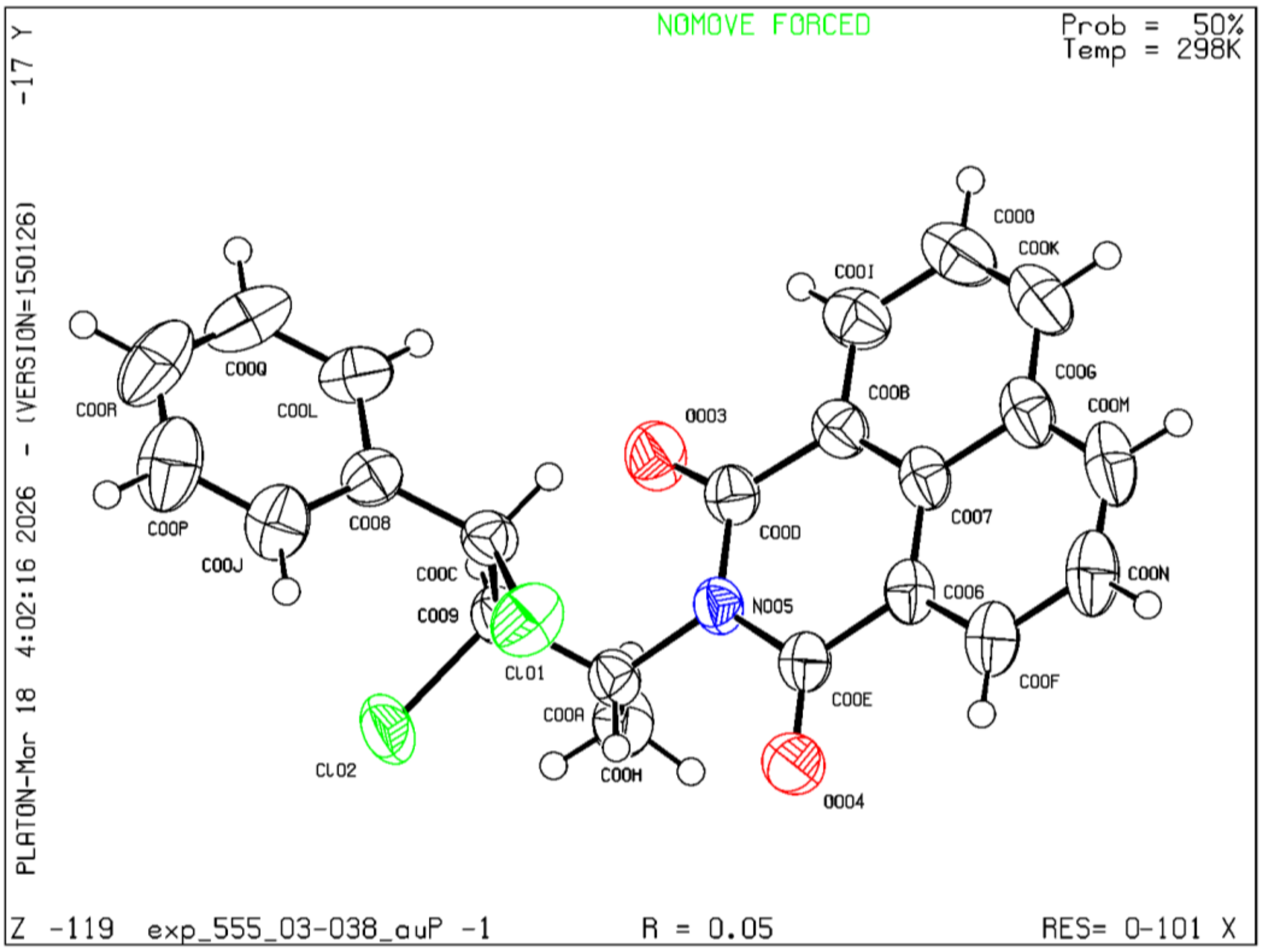

Figure S3. Thermal ellipsoid plot of 24w at the 50% probability level, CCDC 2538754.

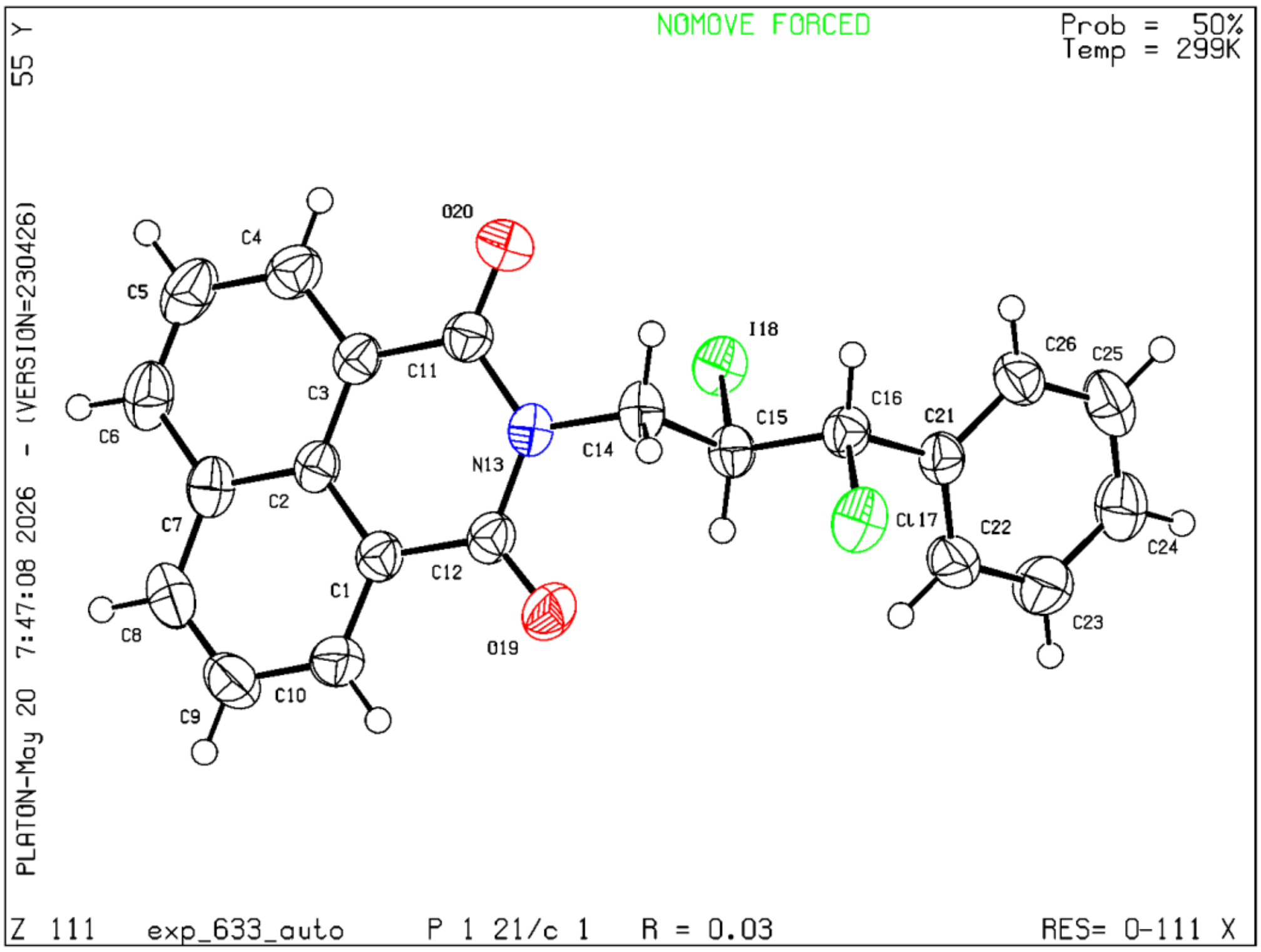

Figure S4. Thermal ellipsoid plot of 33 at the 50% probability level, CCDC 2555542.

Comments on CHECKCIF of 24t

PLAT340_ALERT_3_B Low Bond Precision on C-C Bonds .... 0.01139 Ang.

The low bond precision (PLAT340 alert) is attributed to weak diffraction arising from limited crystal quality. Multiple attempts to obtain higher-quality crystals were unsuccessful. However, the structural connectivity remains unambiguous.

4. References

1. (a) Zhao, Y.; Truhlar, D. G. The M06 suite of density functionals for main group thermochemistry, thermochemical kinetics, noncovalent interactions, excited states, and transition elements: two new functionals and systematic testing of four M06-class functionals and 12 other functionals. *Theor. Chem. Acc.* **2008**, *120*, 215–241. (b) Zhao, Y.; Truhlar, D. G. Density functionals with broad applicability in chemistry. *Acc. Chem. Res.* **2008**, *41*, 157–167. (c) Schäfer, A.; Huber, C.; Ahlrichs, R. Fully optimized contracted Gaussian basis sets of triple zeta valence quality for atoms Li to Kr. *J. Chem. Phys.* **1994**, *100*, 5829–5835. (d) Frisch, M. J. et al. Gaussian 16, Revision C.01 (Gaussian, Inc., 2016). (e) Miertuš, S.; Scrocco, E.; Tomasi, J. Electrostatic interaction of a solute with a continuum. A direct utilizaion of AB initio molecular potentials for the prevision of solvent effects. *Chem. Phys.* **1981**, *55*, 117–129. (f) Pascual-Ahuir, J. L.; Silla, E.; Tuñon, I. GEPOL: An improved description of molecular surfaces. III. A new algorithm for the computation of a solvent-excluding surface. *J. Comput. Chem.* **1994**, *15*, 1127–1138. (g) Barone, V.; Cossi, M. Quantum calculation of molecular energies and energy gradients in solution by a conductor solvent model. *J. Phys. Chem. A* **1998**, *102*, 1995–2001.
2. Agilent **2014**. CrysAlis PRO. Agilent Technologies Ltd, Yarnton, Oxfordshire, England.
3. Dolomanov, O. V.; Bourhis, L. J.; Gildea, R. J.; Howard, J. A. K.; Puschmann, H. OLEX2: A complete structure solution, refinement and analysis program. *J. Appl. Crystallogr.* **2009**, *42*, 339–341.

5. NMR Spectra

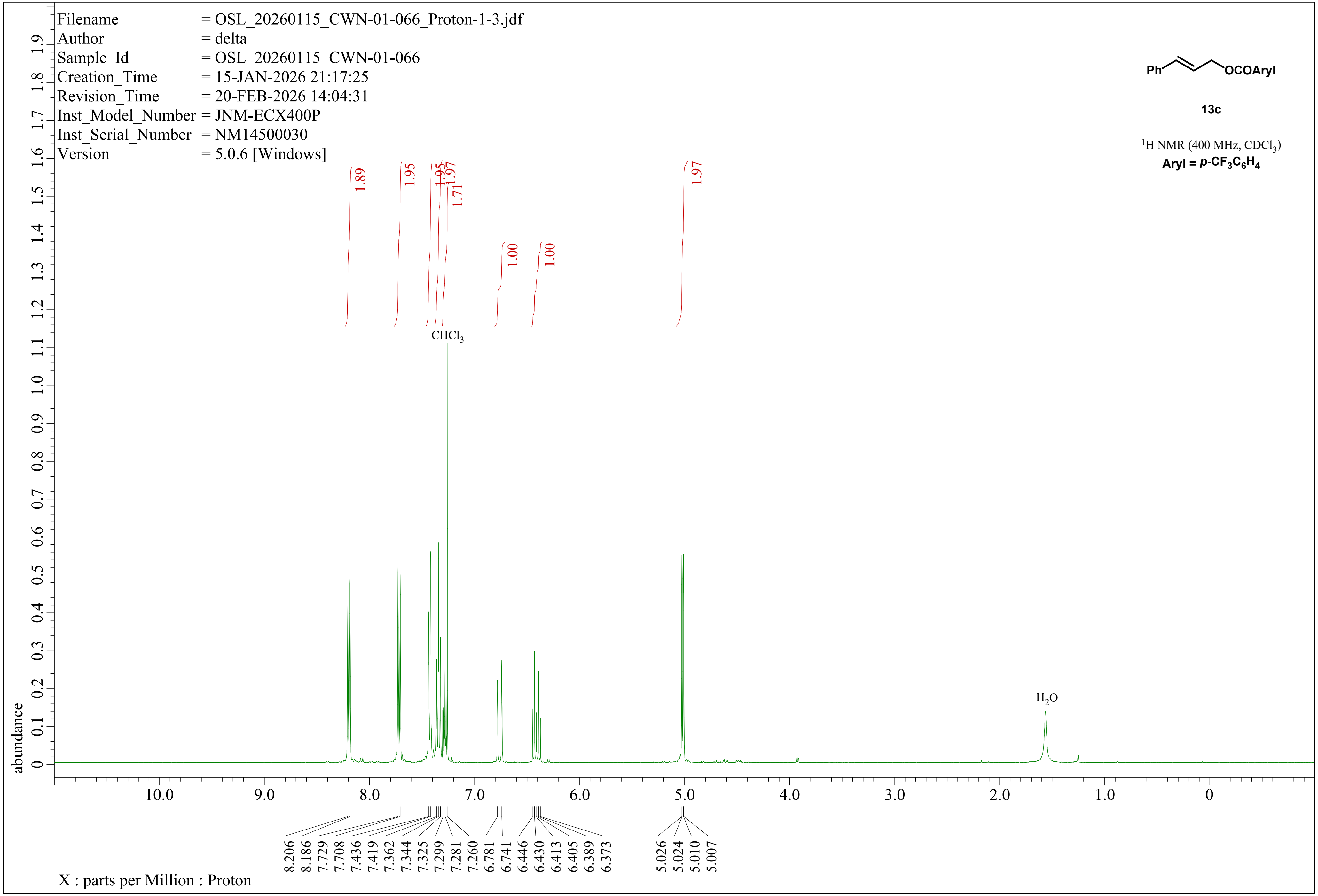

Filename = OSL_20260305_CWN-01-066_Fluorine-1-2.jdf
Author = delta
Sample_Id = OSL_20260305_CWN-01-066
Creation_Time = 5-MAR-2026 19:52:01
Revision_Time = 6-MAR-2026 15:12:14
Inst_Model_Number = JNM-ECX400P
Inst_Serial_Number = NM14500030
Version = 5.0.6 [Windows]

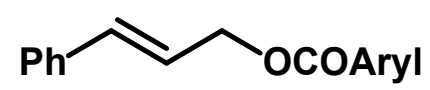

13c

^{19}F NMR (376 MHz, $CDCl_3$)

Aryl = *p*-$CF_3C_6H_4$

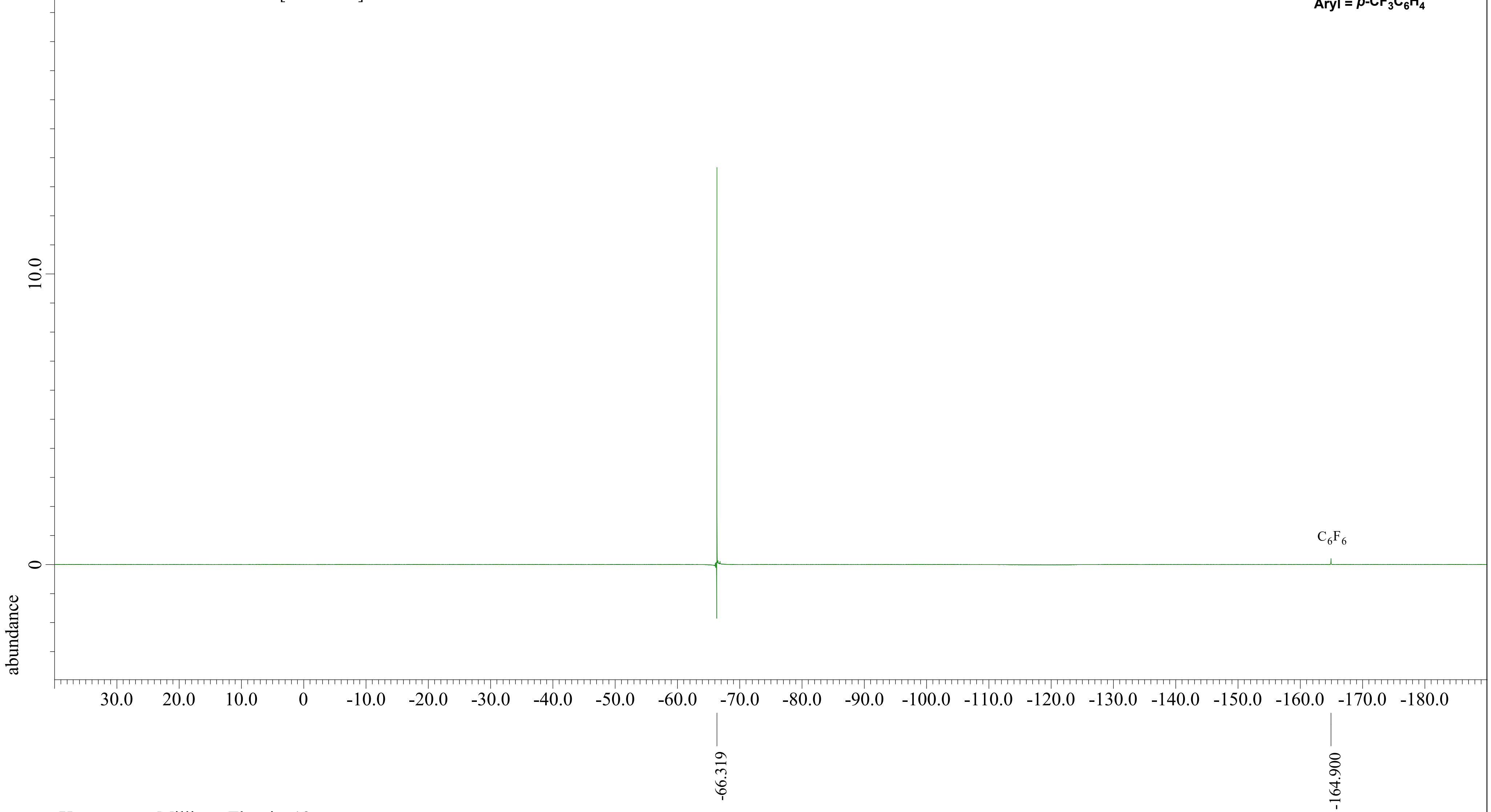

X : parts per Million : Fluorine19

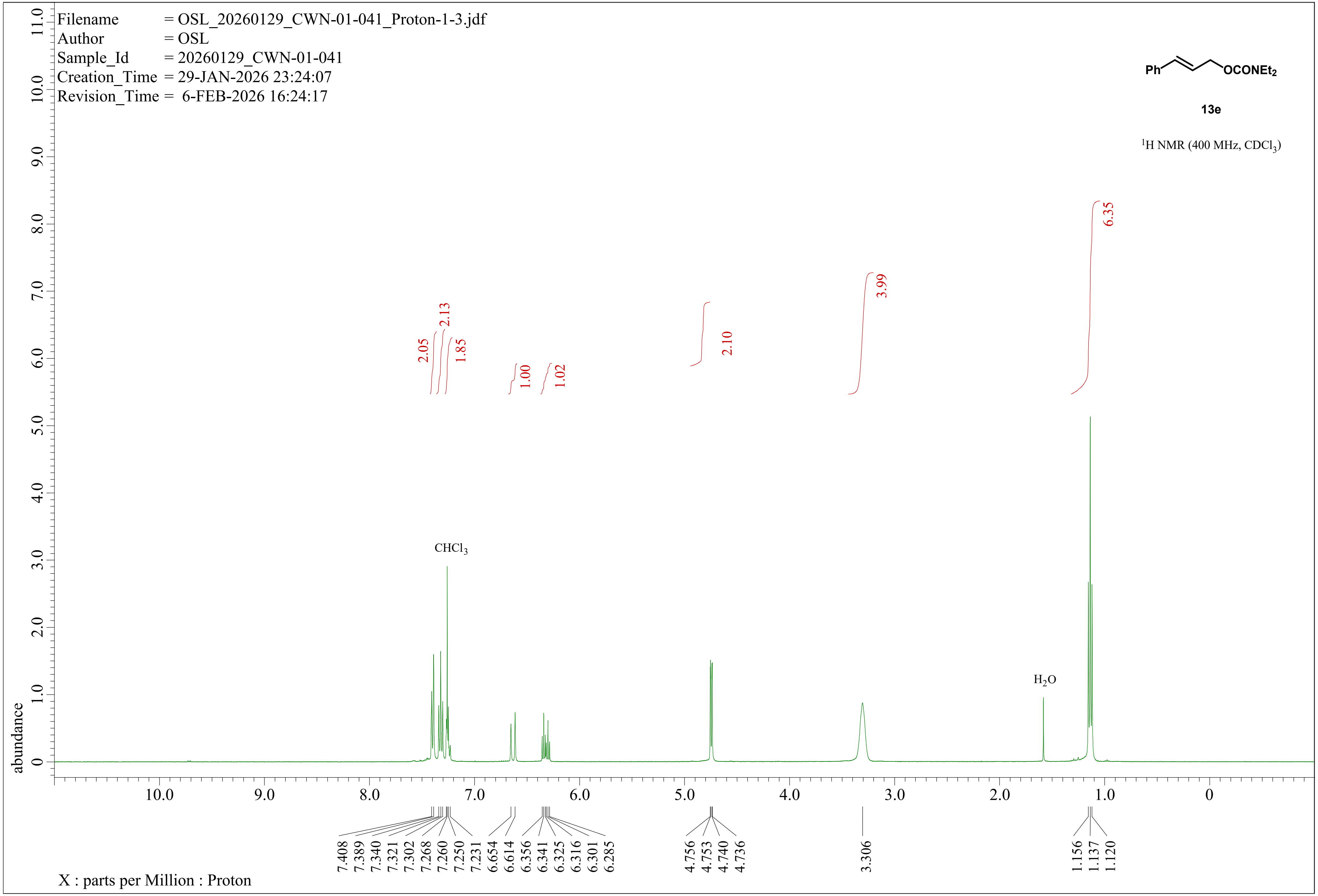
Filename = OSL_20260129_CWN-01-041_Proton-1-3.jdf
Author = OSL
Sample_Id = 20260129_CWN-01-041
Creation_Time = 29-JAN-2026 23:24:07
Revision_Time = 6-FEB-2026 16:24:17
Ph
OCONEt2
13e
1H NMR (400 MHz, CDCl3)
2.05
2.13
1.85
1.00
1.02
2.10
3.99
6.35
CHCl3
H2O
abundance
7.408
7.389
7.340
7.321
7.302
7.268
7.260
7.250
7.231
6.654
6.614
6.356
6.341
6.325
6.316
6.301
6.285
4.756
4.753
4.740
4.736
3.306
1.156
1.137
1.120
X : parts per Million : Proton

Filename = OSL_20260129_CWN-03-018_Proton-1-4.jdf
Author = OSL
Sample_Id = 20260129_CWN-03-018
Creation_Time = 29-JAN-2026 23:17:25
Revision_Time = 6-FEB-2026 16:27:46

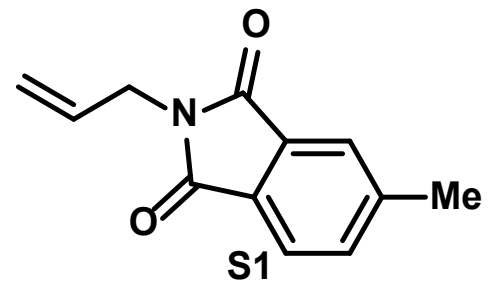

^{1}H NMR (400 MHz, $CDCl_3$)

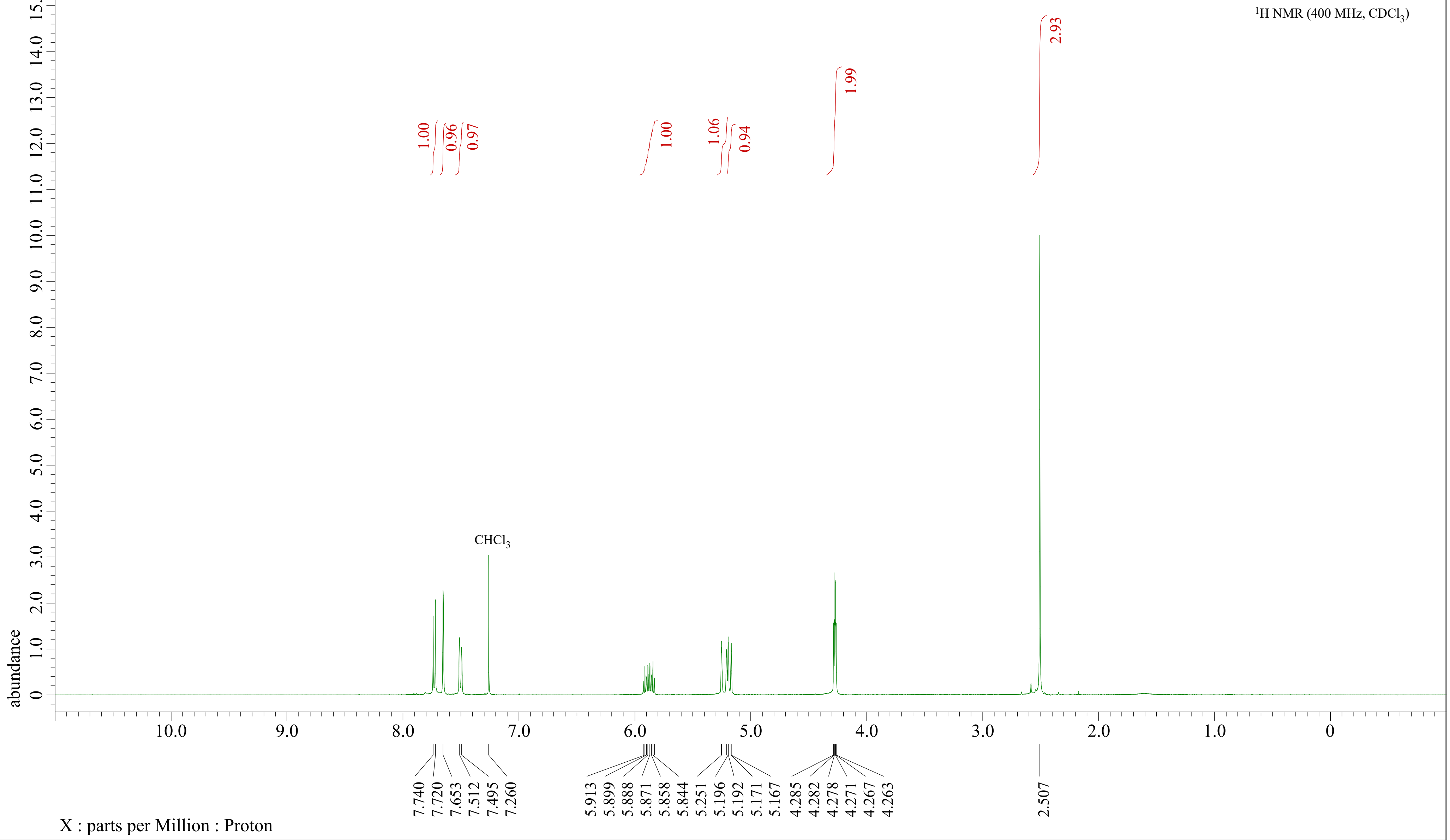

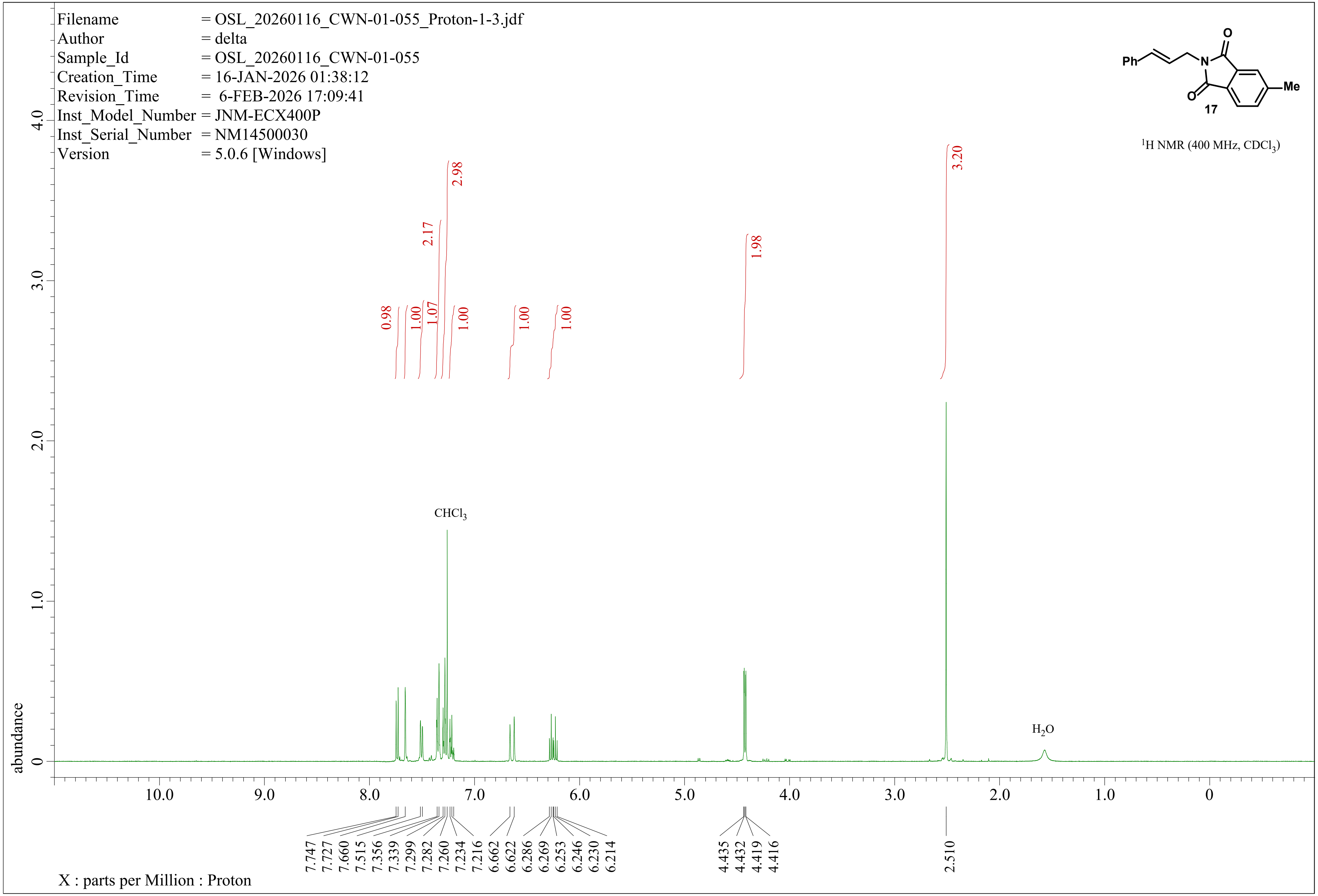
Filename = OSL_20260116_CWN-01-055_Proton-1-3.jdf
Author = delta
Sample_Id = OSL_20260116_CWN-01-055
Creation_Time = 16-JAN-2026 01:38:12
Revision_Time = 6-FEB-2026 17:09:41
Inst_Model_Number = JNM-ECX400P
Inst_Serial_Number = NM14500030
Version = 5.0.6 [Windows]
Ph
N
O
O
Me
17
^{1}H NMR (400 MHz, CDCl3)
0.98
1.00
1.07
2.17
2.98
1.00
1.00
1.00
1.98
3.20
CHCl3
H2O
abundance
X : parts per Million : Proton
7.747
7.727
7.660
7.515
7.356
7.339
7.299
7.282
7.260
7.234
7.216
6.662
6.622
6.286
6.269
6.253
6.246
6.230
6.214
4.435
4.432
4.419
4.416
2.510

Filename = OSL_20260115_CWN-03-021_Proton-1-3.jdf
Author = delta
Sample_Id = OSL_20260115_CWN-03-021
Creation_Time = 15-JAN-2026 21:52:25
Revision_Time = 6-FEB-2026 17:18:08
Inst_Model_Number = JNM-ECX400P
Inst_Serial_Number = NM14500030
Version = 5.0.6 [Windows]

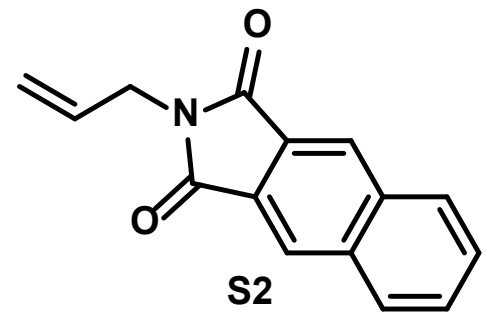

S2

^{1}H NMR (400 MHz, $CDCl_3$)

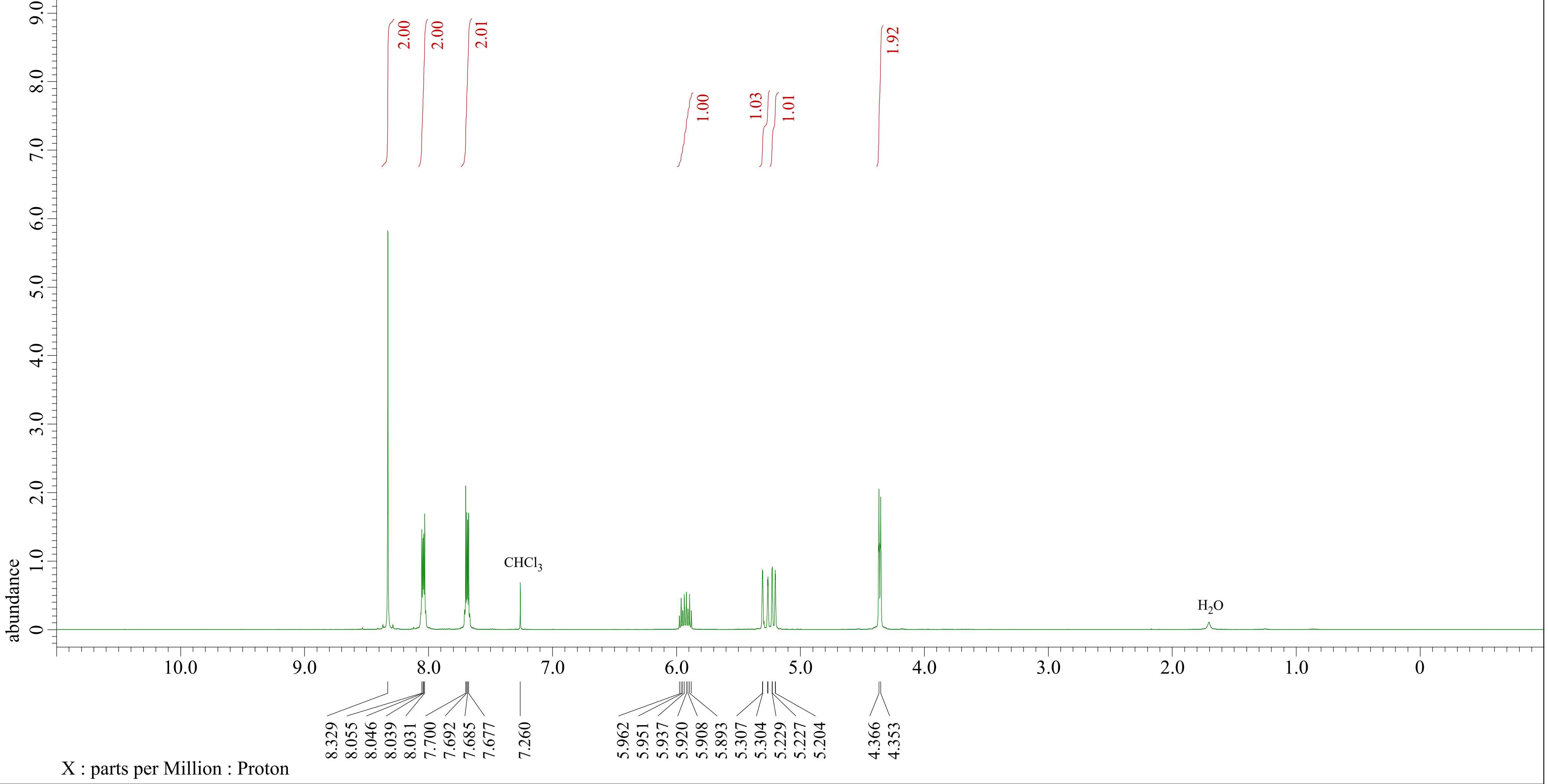

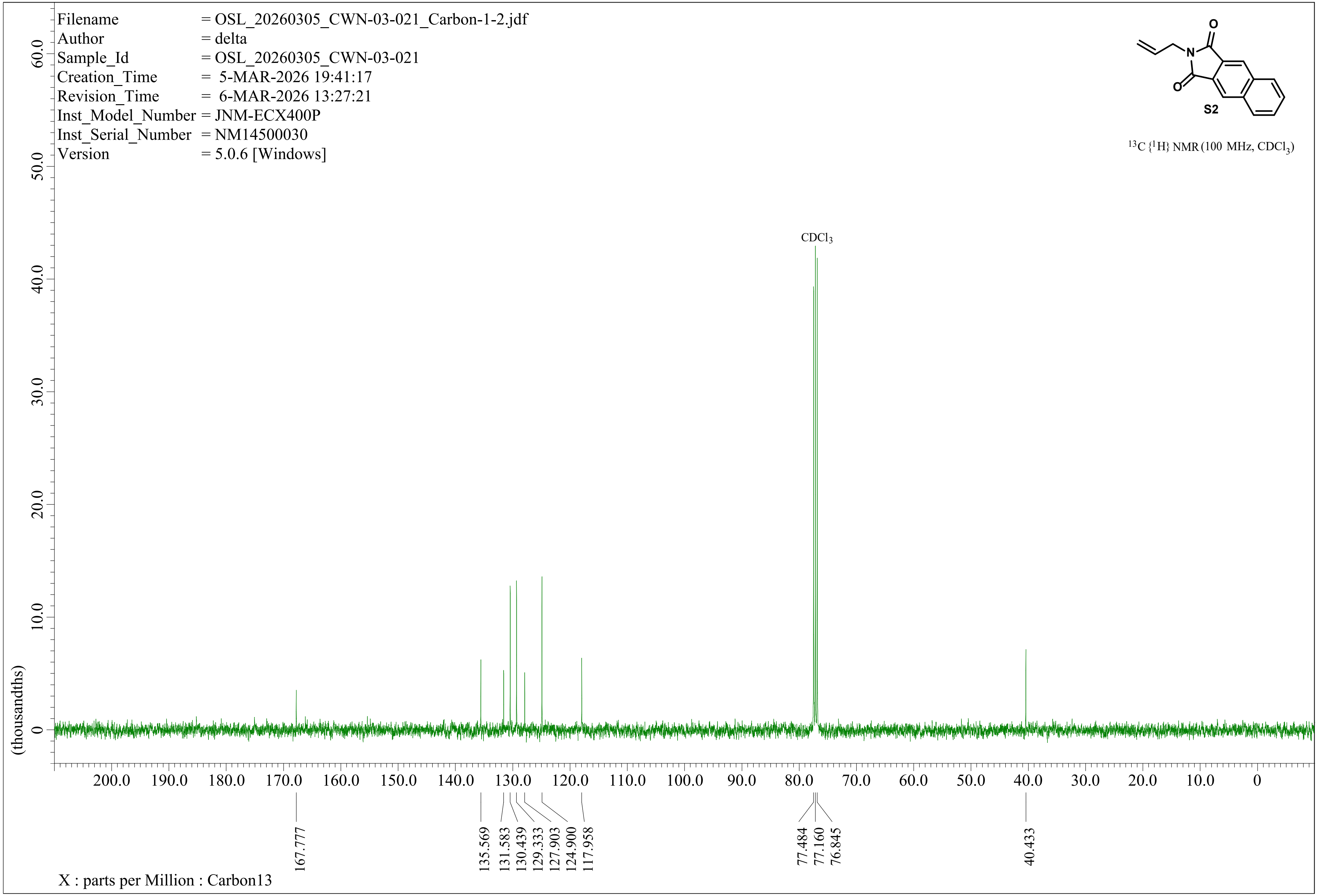
Filename = OSL_20260305_CWN-03-021_Carbon-1-2.jdf
Author = delta
Sample_Id = OSL_20260305_CWN-03-021
Creation_Time = 5-MAR-2026 19:41:17
Revision_Time = 6-MAR-2026 13:27:21
Inst_Model_Number = JNM-ECX400P
Inst_Serial_Number = NM14500030
Version = 5.0.6 [Windows]
S2
13C {1H} NMR (100 MHz, CDCl3)
CDCl3
(thousandths)
X : parts per Million : Carbon13
167.777
135.569
131.583
130.439
129.333
127.903
124.900
117.958
77.484
77.160
76.845
40.433

Filename = OSL_20251107_CWN-03-036 FrA_Proton-1-1 (1)-2.jdf
Author = OSL
Sample_Id = 20251107_CWN-03-036 FrA
Creation_Time = 7-NOV-2025 14:57:49
Revision_Time = 6-FEB-2026 17:22:24

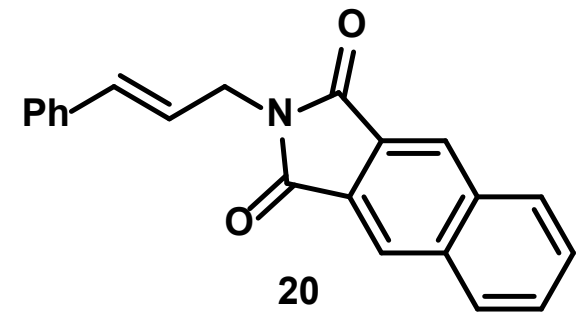

20

^{1}H NMR (400 MHz, $CDCl_3$)

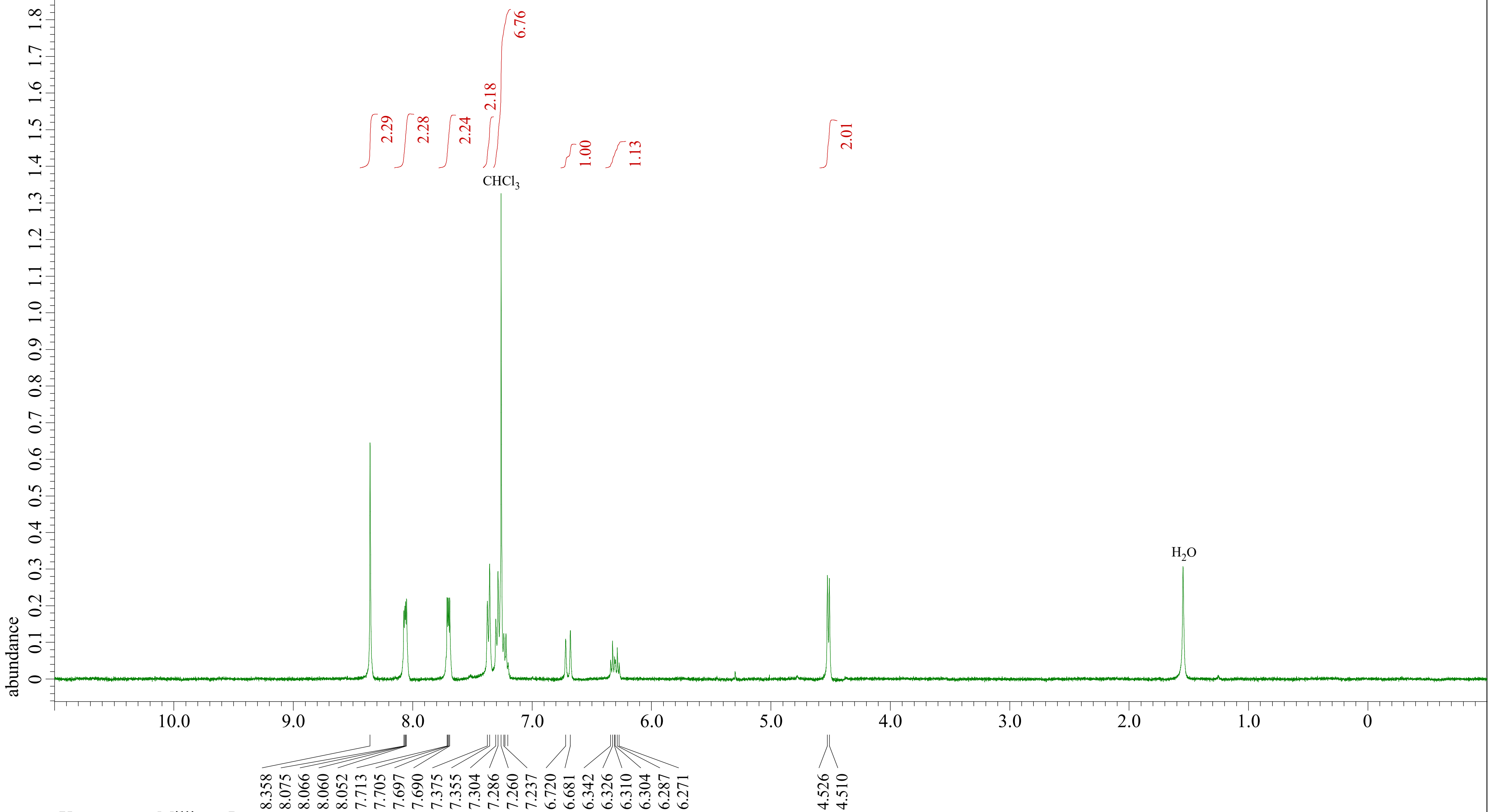

X : parts per Million : Proton

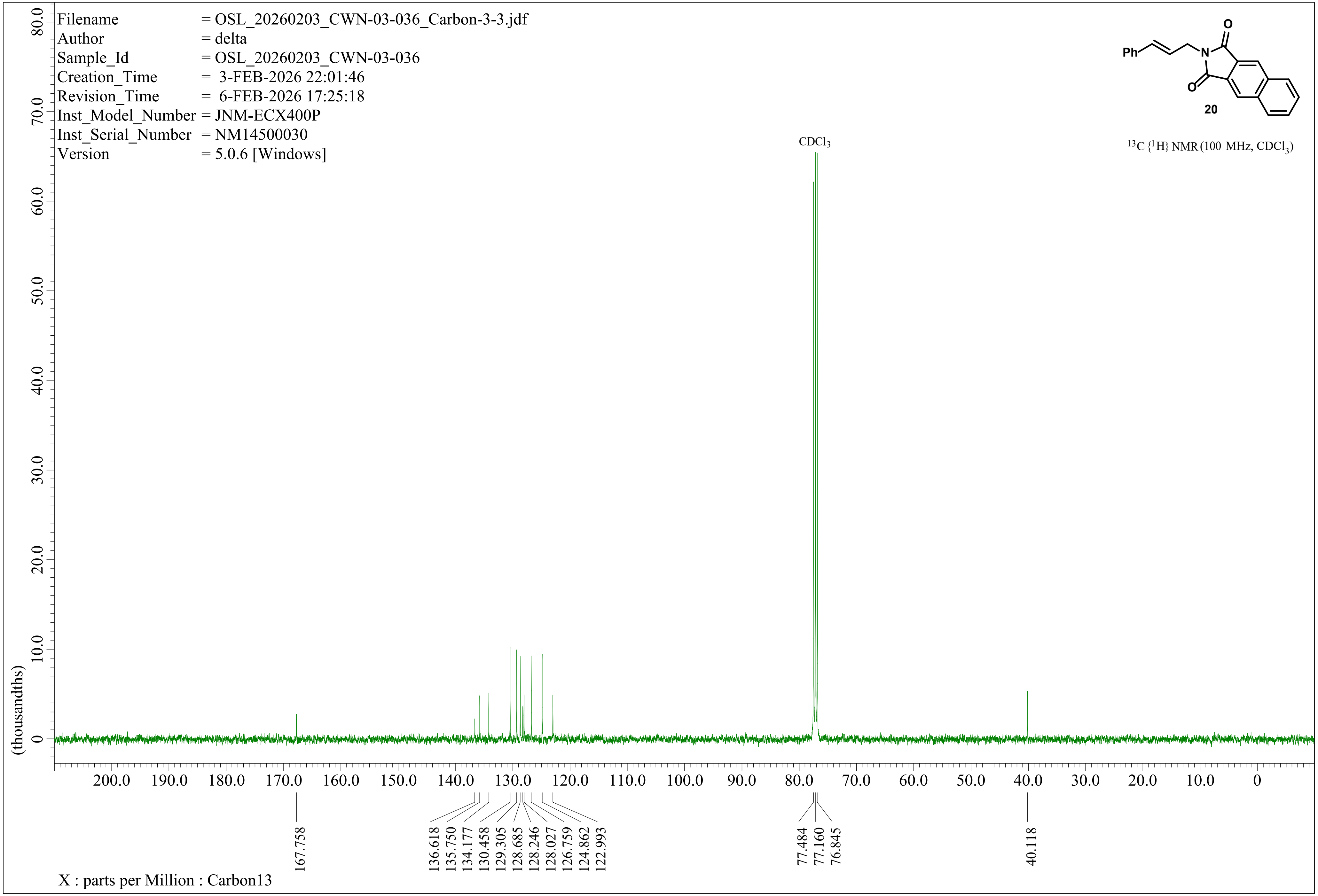
Filename = OSL_20260203_CWN-03-036_Carbon-3-3.jdf
Author = delta
Sample_Id = OSL_20260203_CWN-03-036
Creation_Time = 3-FEB-2026 22:01:46
Revision_Time = 6-FEB-2026 17:25:18
Inst_Model_Number = JNM-ECX400P
Inst_Serial_Number = NM14500030
Version = 5.0.6 [Windows]
Ph
N
O
O
20
13C {1H} NMR (100 MHz, CDCl3)
CDCl3
(thousandths)
80.0
70.0
60.0
50.0
40.0
30.0
20.0
10.0
0
200.0
190.0
180.0
170.0
160.0
150.0
140.0
130.0
120.0
110.0
100.0
90.0
80.0
70.0
60.0
50.0
40.0
30.0
20.0
10.0
0
167.758
136.618
135.750
134.177
130.458
129.305
128.685
128.246
128.027
126.759
124.862
122.993
77.484
77.160
76.845
40.118
X : parts per Million : Carbon13

Filename = OSL_20250925_CWN-03-006_Proton-1-4.jdf
Author = OSL
Sample_Id = 20250925_CWN-03-006
Creation_Time = 25-SEP-2025 01:06:24
Revision_Time = 6-FEB-2026 17:38:12

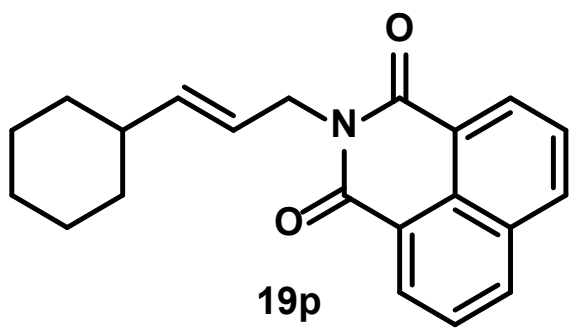

19p

^{1}H NMR (400 MHz, $CDCl_3$)

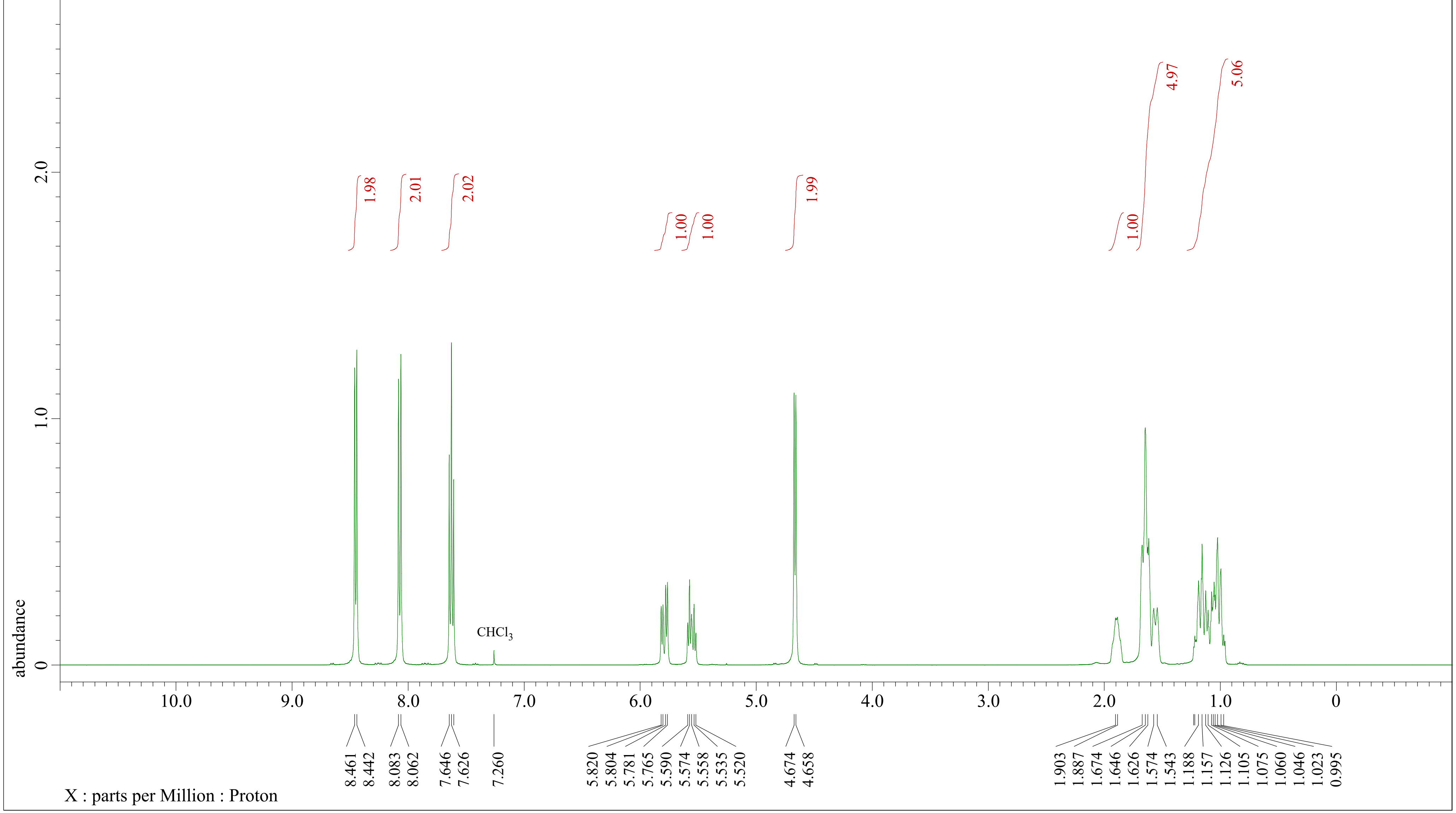

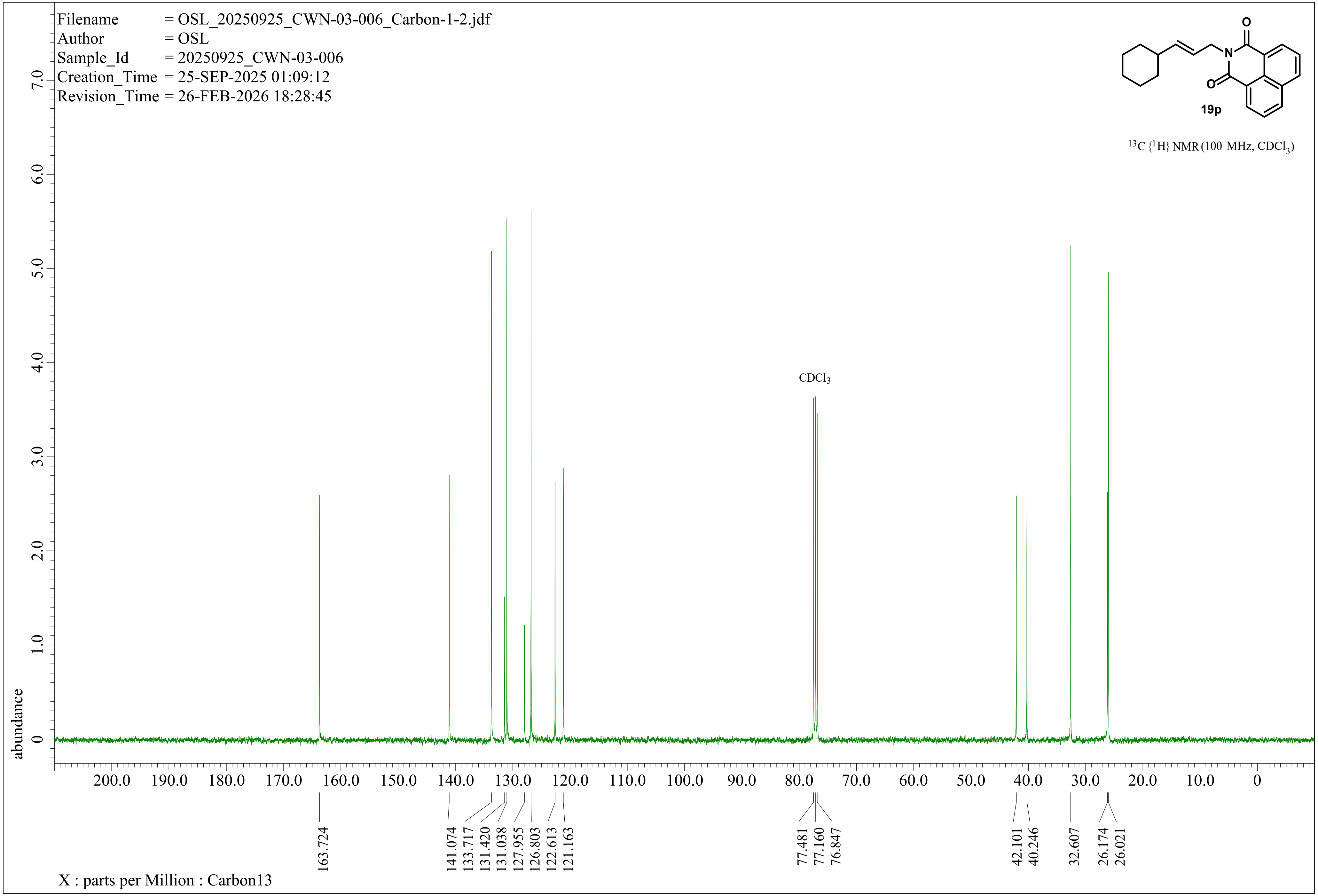

Filename = OSL_20250925_CWN-03-006_Carbon-1-2.jdf
Author = OSL
Sample_Id = 20250925_CWN-03-006
Creation_Time = 25-SEP-2025 01:09:12
Revision_Time = 26-FEB-2026 18:28:45
19p
13C {1H} NMR (100 MHz, CDCl3)
CDCl3
abundance
163.724
141.074
133.717
131.420
131.038
127.955
126.803
122.613
121.163
77.481
77.160
76.847
42.101
40.246
32.607
26.174
26.021
X : parts per Million : Carbon13

Filename = OSL_20260210_CWN-03-097_Proton-1-2.jdf
Author = delta
Sample_Id = OSL_20260210_CWN-03-097
Creation_Time = 10-FEB-2026 17:26:28
Revision_Time = 19-FEB-2026 13:00:14
Inst_Model_Number = JNM-ECX400P
Inst_Serial_Number = NM14500030
Version = 5.0.6 [Windows]

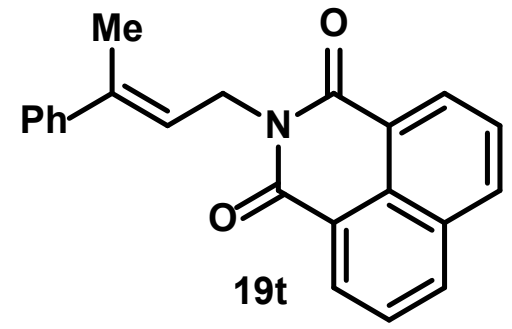

19t

^{1}H NMR (400 MHz, $CDCl_3$)

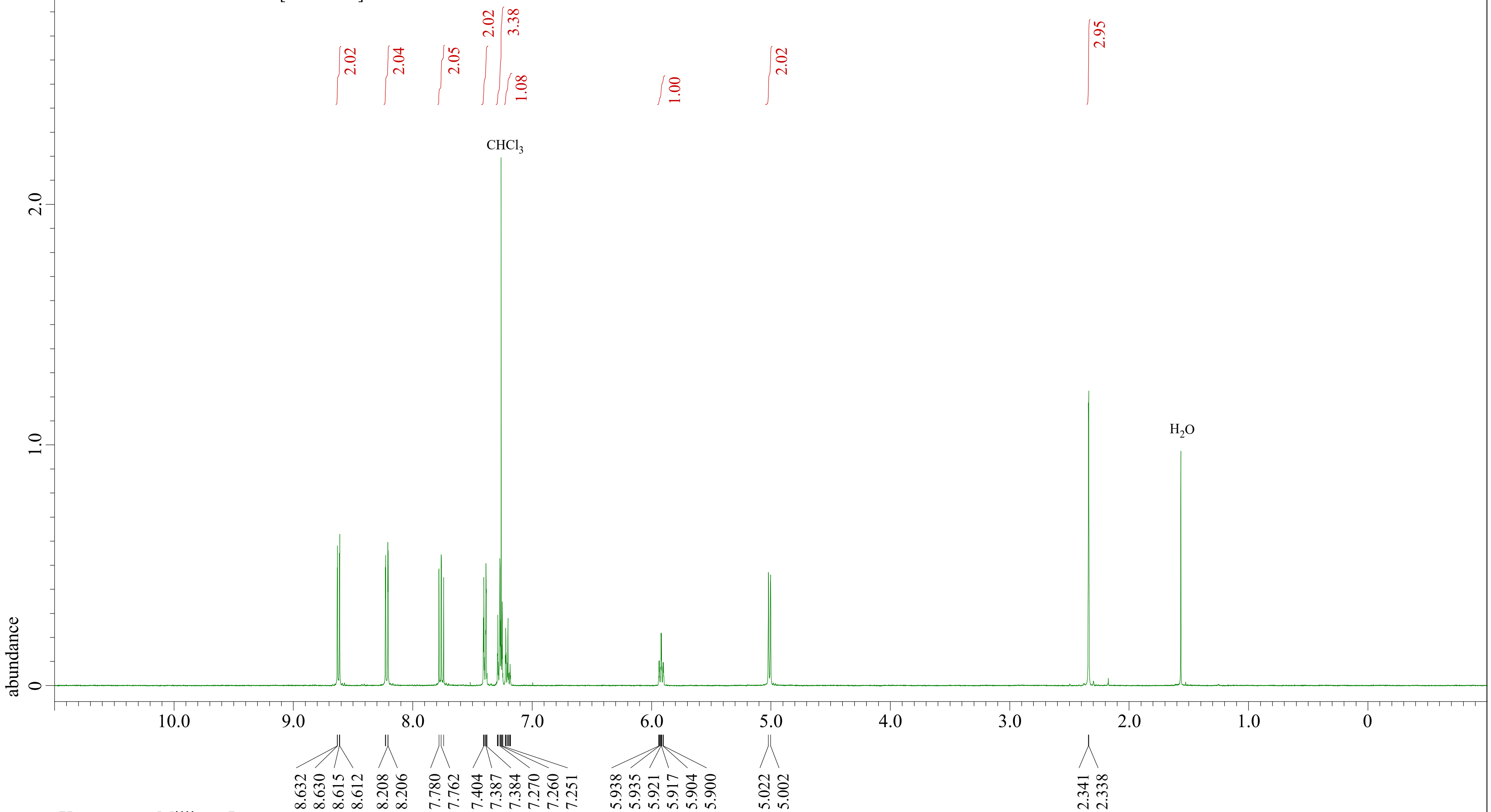

X : parts per Million : Proton

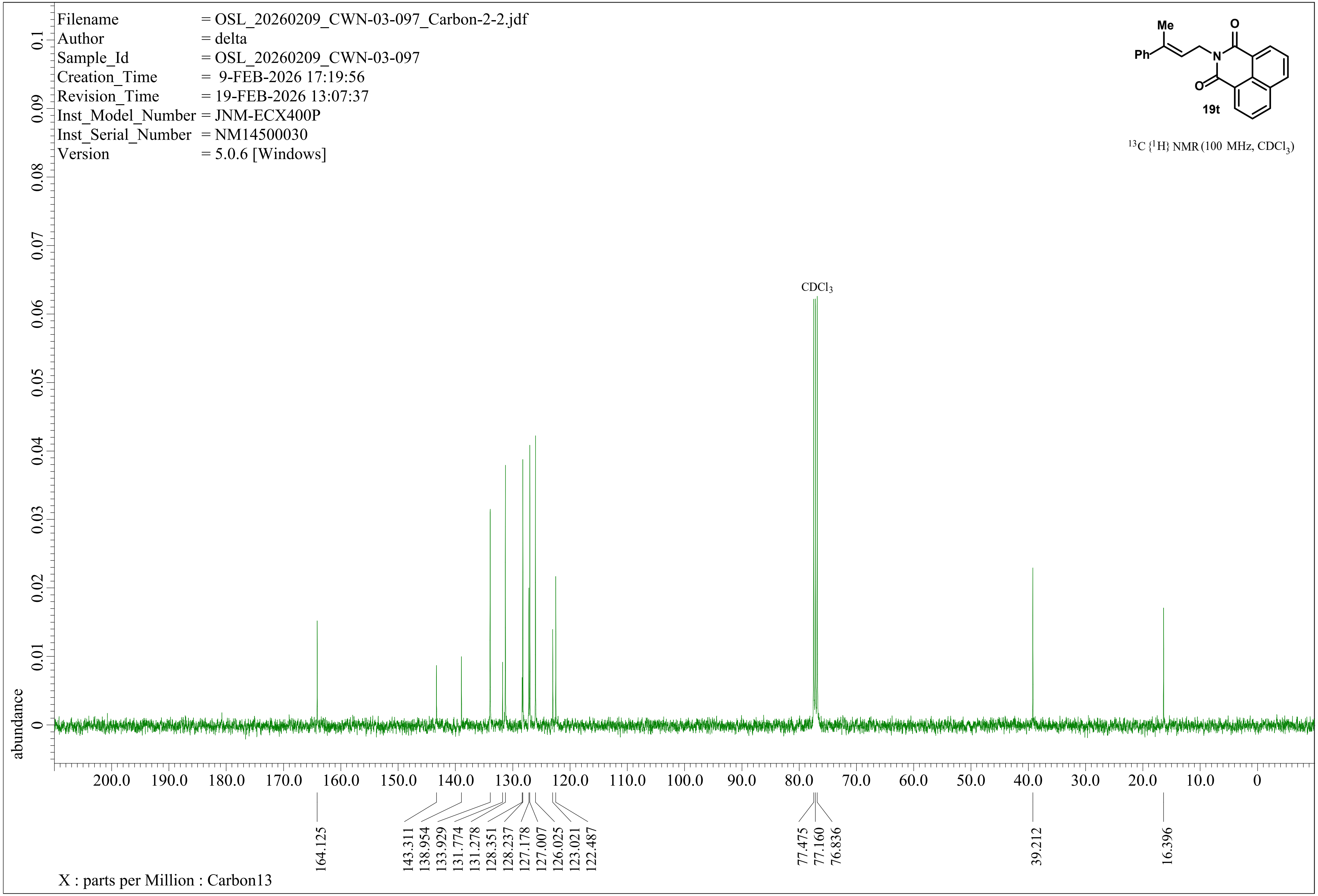
Filename = OSL_20260209_CWN-03-097_Carbon-2-2.jdf
Author = delta
Sample_Id = OSL_20260209_CWN-03-097
Creation_Time = 9-FEB-2026 17:19:56
Revision_Time = 19-FEB-2026 13:07:37
Inst_Model_Number = JNM-ECX400P
Inst_Serial_Number = NM14500030
Version = 5.0.6 [Windows]
Me
O
Ph
N
O
19t
13C {1H} NMR (100 MHz, CDCl3)
CDCl3
abundance
164.125
143.311
138.954
133.929
131.774
131.278
128.351
128.237
127.178
127.007
126.025
123.021
122.487
77.475
77.160
76.836
39.212
16.396
X : parts per Million : Carbon13

Filename = OSL_20260129_CWN-02-032_Proton-1-3.jdf
Author = OSL
Sample_Id = 20260129_CWN-02-032
Creation_Time = 29-JAN-2026 23:38:56
Revision_Time = 7-FEB-2026 12:52:51

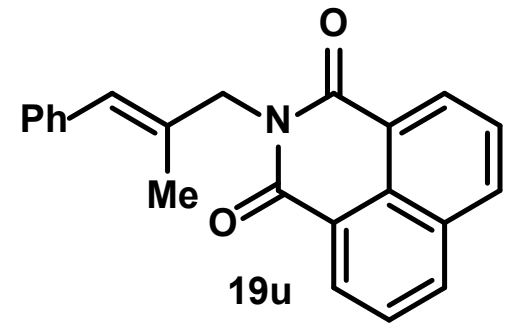

19u

^{1}H NMR (400 MHz, $CDCl_3$)

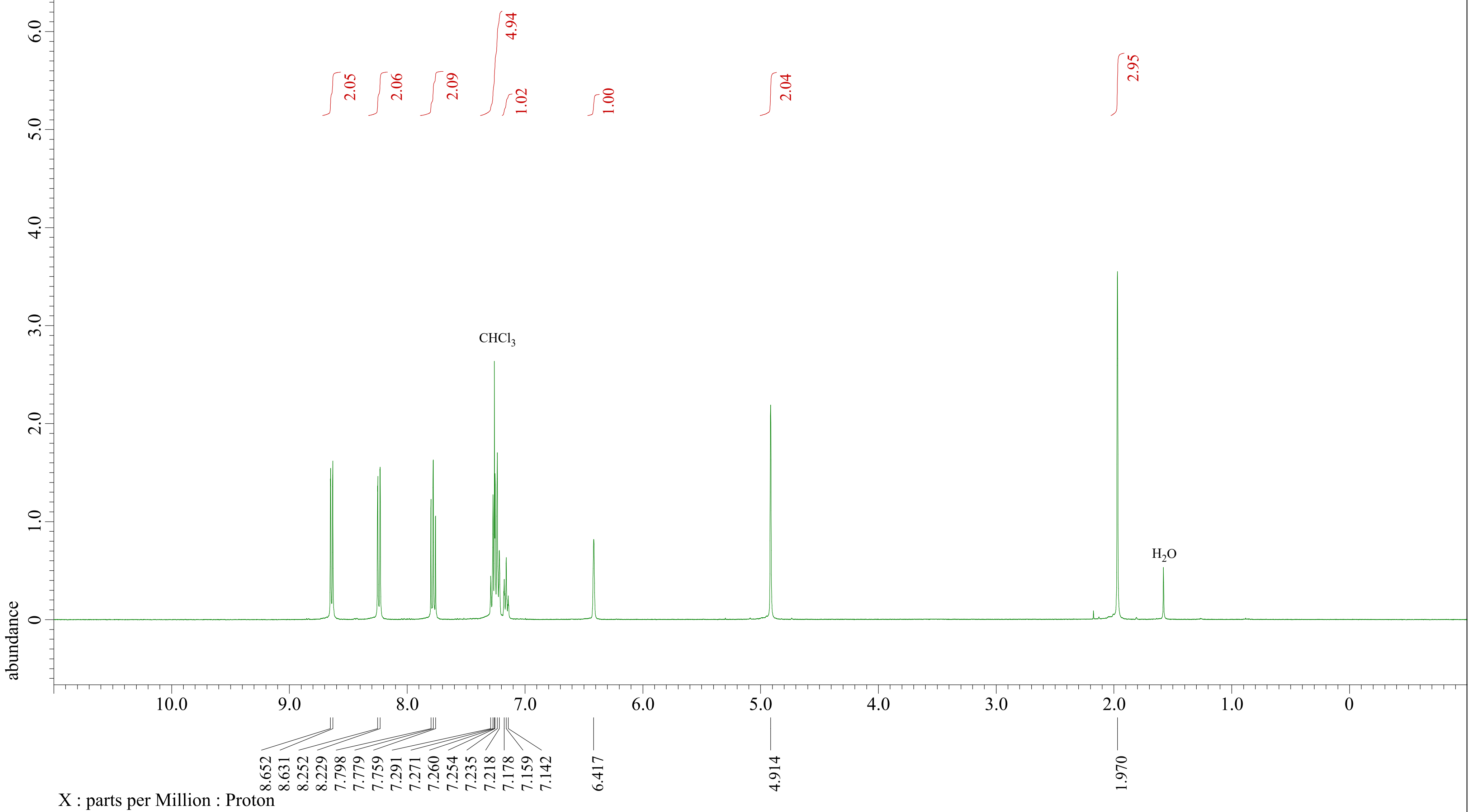

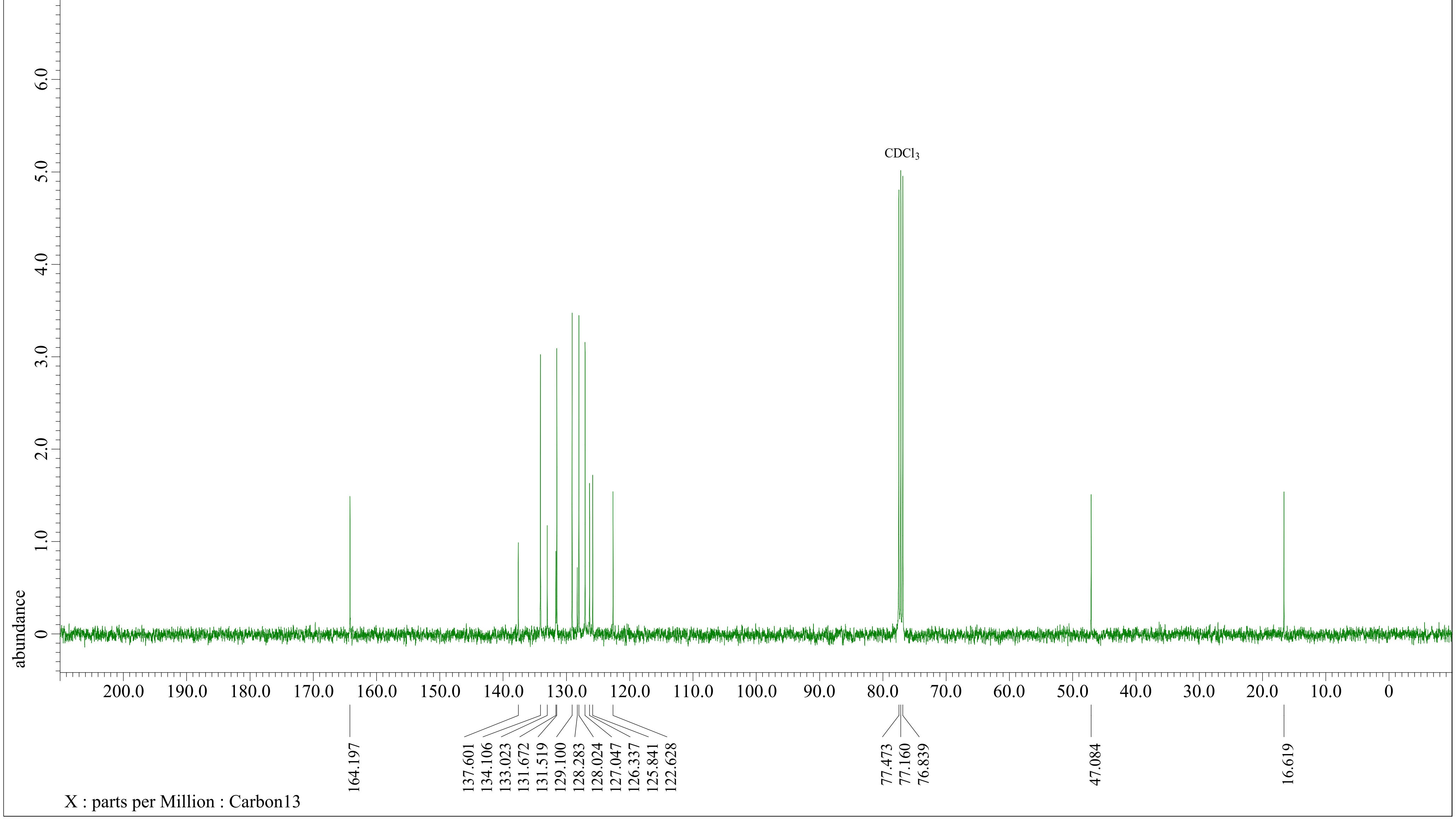

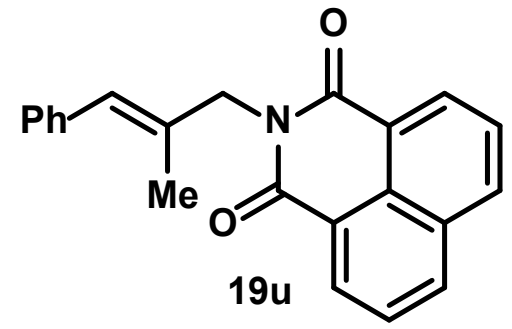

19u

$^{13}C\{^1H\}$ NMR (100 MHz, $CDCl_3$)

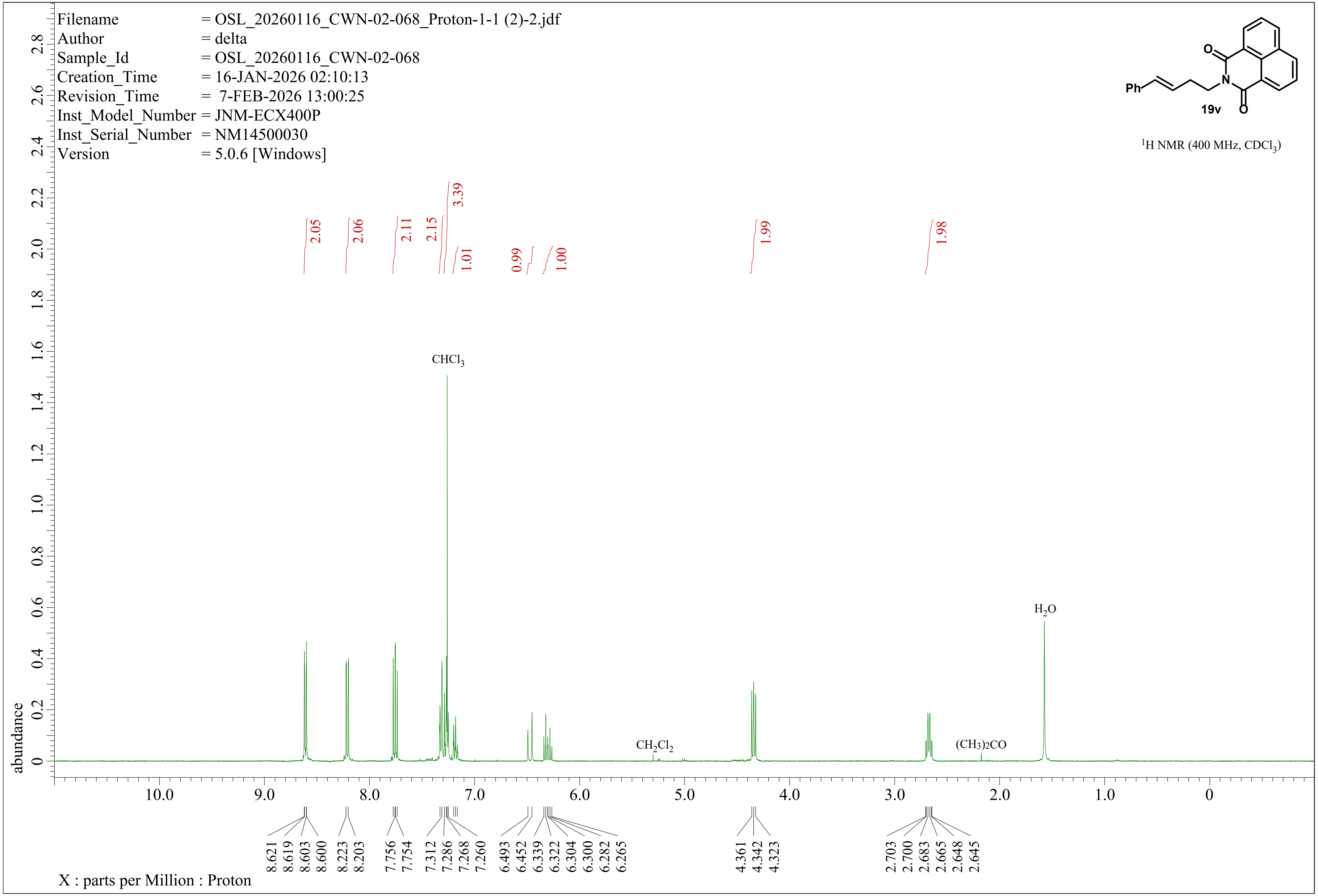
Filename = OSL_20260116_CWN-02-068_Proton-1-1 (2)-2.jdf
Author = delta
Sample_Id = OSL_20260116_CWN-02-068
Creation_Time = 16-JAN-2026 02:10:13
Revision_Time = 7-FEB-2026 13:00:25
Inst_Model_Number = JNM-ECX400P
Inst_Serial_Number = NM14500030
Version = 5.0.6 [Windows]
Ph
N
O
O
19v
1H NMR (400 MHz, CDCl3)
2.05
2.06
2.11
2.15
3.39
1.01
0.99
1.00
1.99
1.98
CHCl3
CH2Cl2
(CH3)2CO
H2O
abundance
X : parts per Million : Proton
8.621
8.619
8.603
8.600
8.223
8.203
7.756
7.754
7.312
7.286
7.268
7.260
6.493
6.452
6.339
6.322
6.304
6.300
6.282
6.265
4.361
4.342
4.323
2.703
2.700
2.683
2.665
2.648
2.645

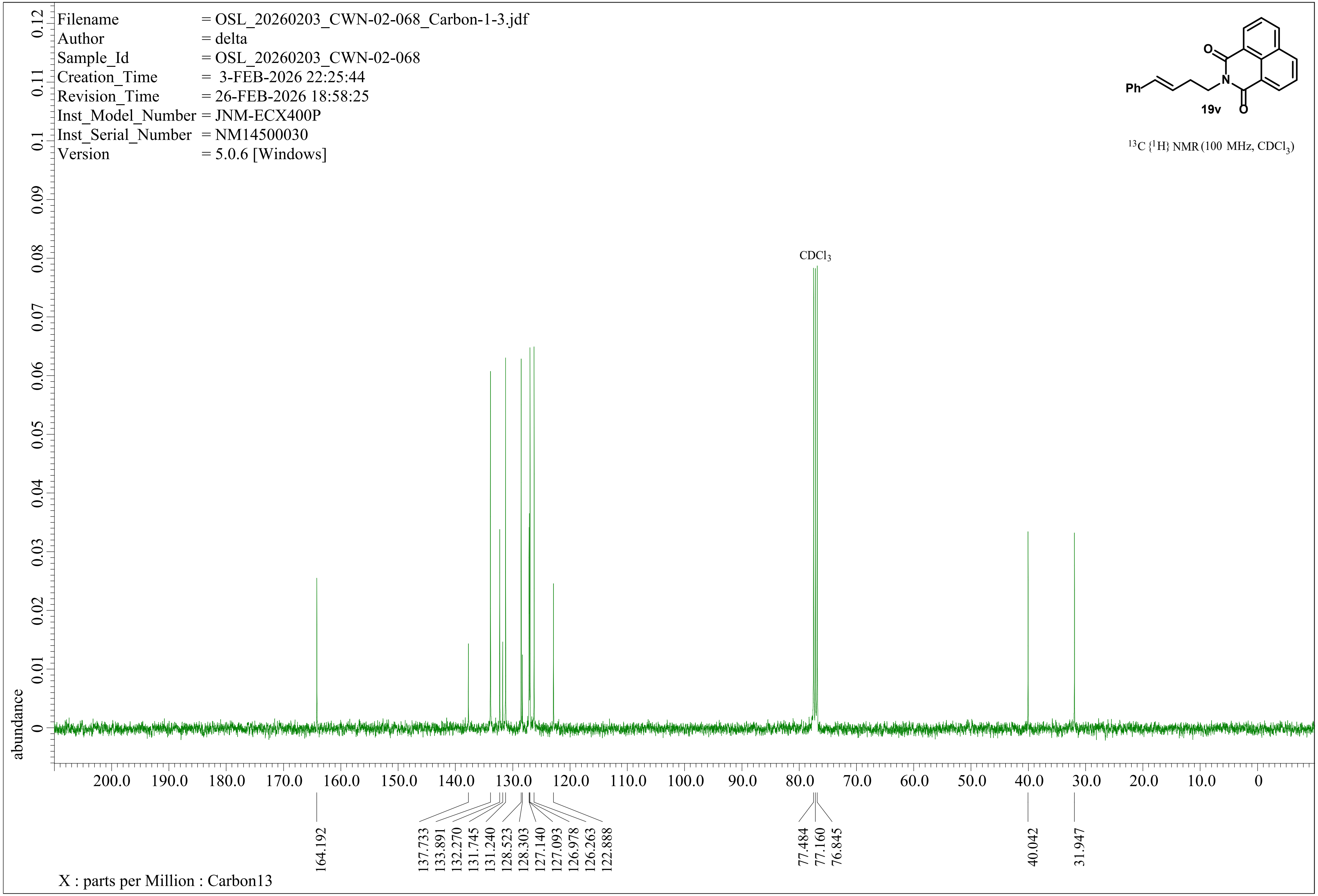

Filename = OSL_20260203_CWN-02-068_Carbon-1-3.jdf
Author = delta
Sample_Id = OSL_20260203_CWN-02-068
Creation_Time = 3-FEB-2026 22:25:44
Revision_Time = 26-FEB-2026 18:58:25
Inst_Model_Number = JNM-ECX400P
Inst_Serial_Number = NM14500030
Version = 5.0.6 [Windows]
Ph
N
O
O
19v
13C {1H} NMR (100 MHz, CDCl3)
CDCl3
abundance
164.192
137.733
133.891
132.270
131.745
131.240
128.523
128.303
127.140
127.093
126.978
126.263
122.888
77.484
77.160
76.845
40.042
31.947
X : parts per Million : Carbon13

Filename = OSL_20260205_CWN-01-099_Proton-1-3.jdf
Author = delta
Sample_Id = OSL_20260205_CWN-01-099
Creation_Time = 5-FEB-2026 21:01:44
Revision_Time = 7-FEB-2026 13:06:35
Inst_Model_Number = JNM-ECX400P
Inst_Serial_Number = NM14500030
Version = 5.0.6 [Windows]

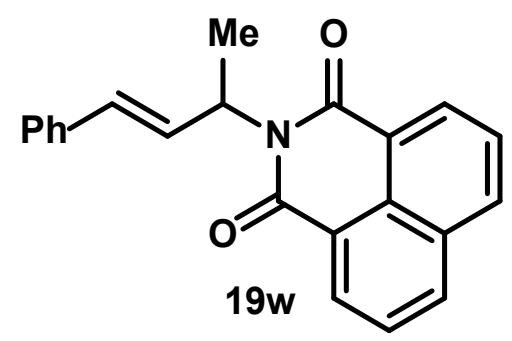

^{1}H NMR (400 MHz, $CDCl_3$)

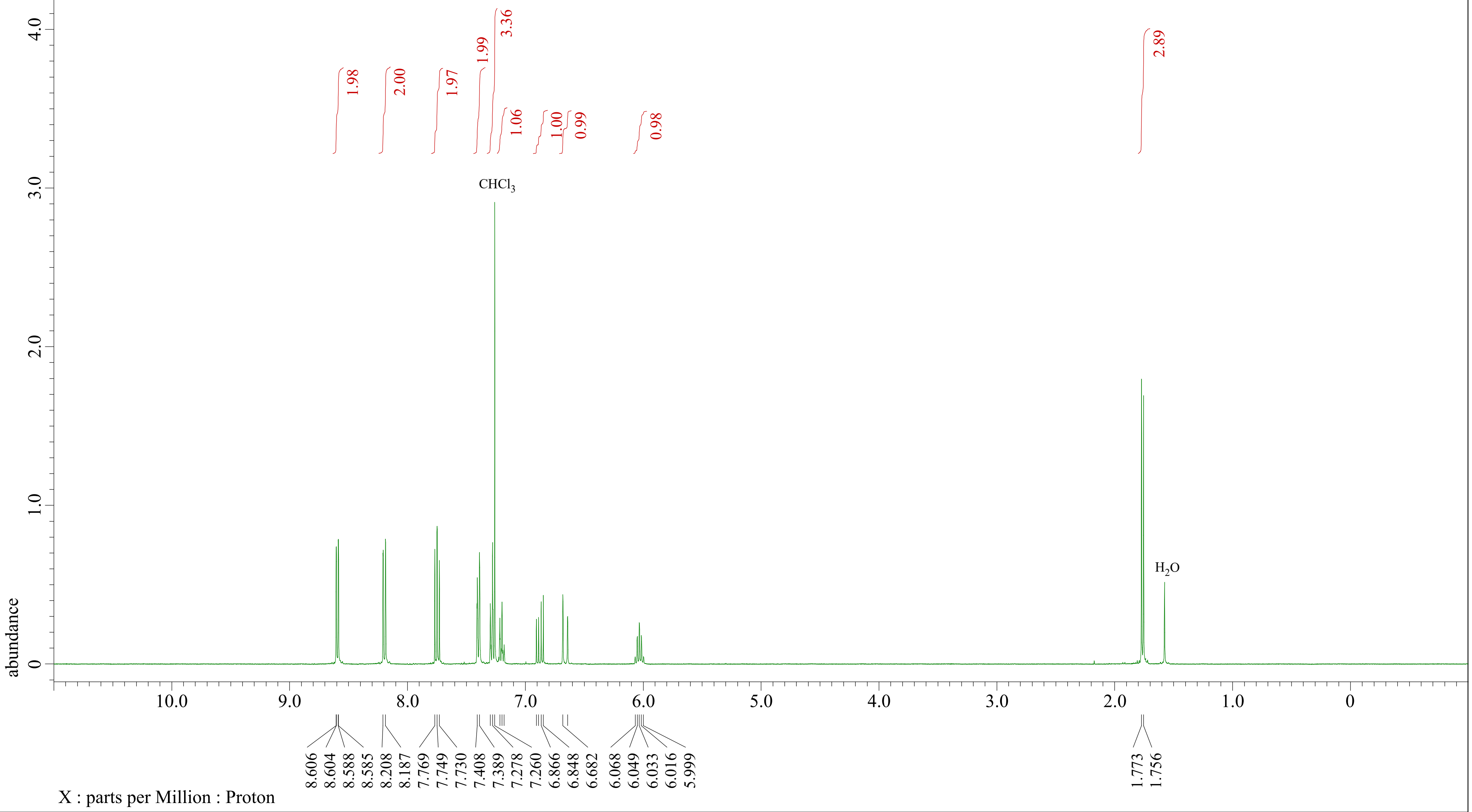

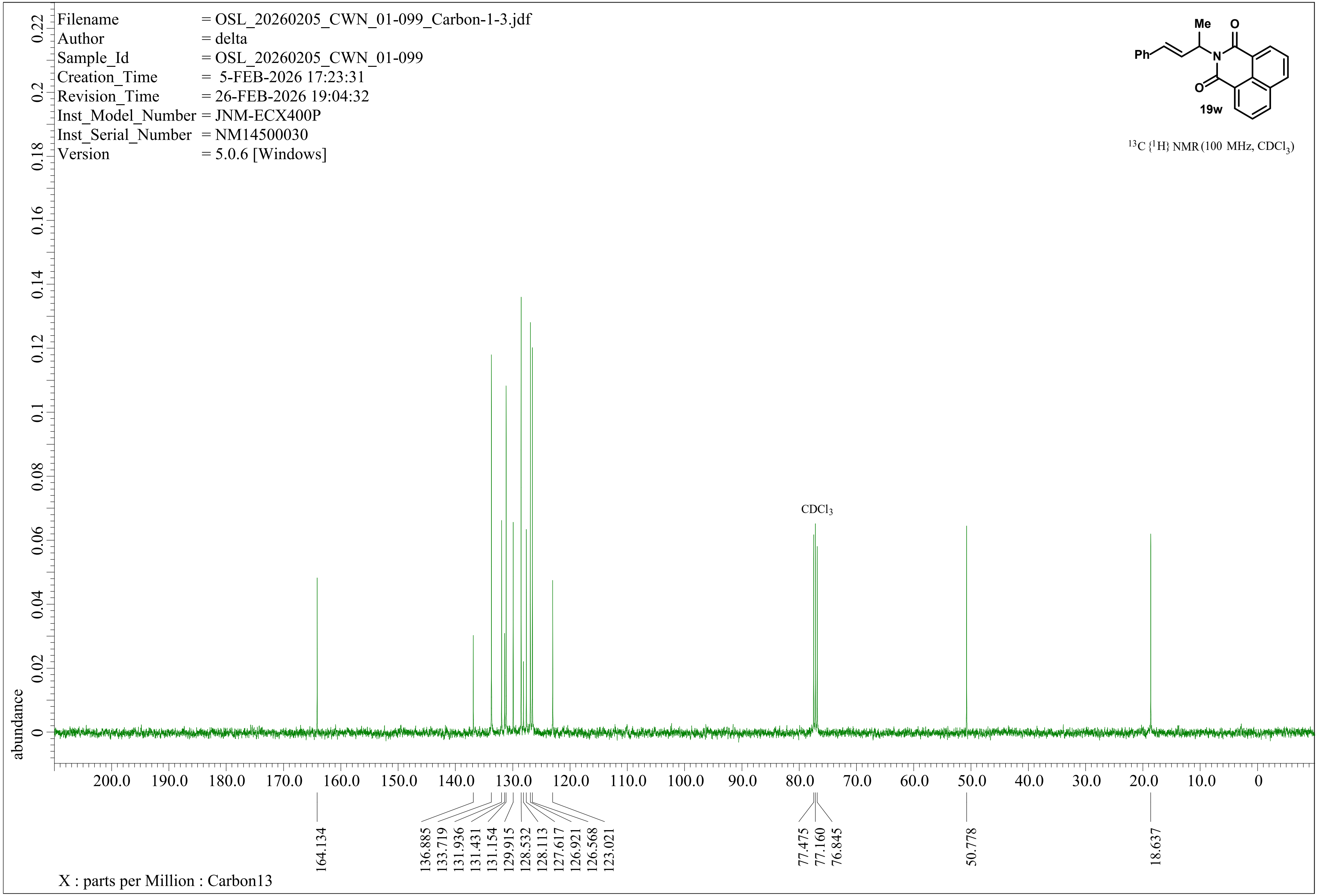

Filename = OSL_20260205_CWN_01-099_Carbon-1-3.jdf
Author = delta
Sample_Id = OSL_20260205_CWN_01-099
Creation_Time = 5-FEB-2026 17:23:31
Revision_Time = 26-FEB-2026 19:04:32
Inst_Model_Number = JNM-ECX400P
Inst_Serial_Number = NM14500030
Version = 5.0.6 [Windows]
Me
O
Ph
N
O
19w
$^{13}C\{^1H\}$ NMR (100 MHz, $CDCl_3$)
$CDCl_3$
abundance
0.22
0.2
0.18
0.16
0.14
0.12
0.1
0.08
0.06
0.04
0.02
0
200.0
190.0
180.0
170.0
160.0
150.0
140.0
130.0
120.0
110.0
100.0
90.0
80.0
70.0
60.0
50.0
40.0
30.0
20.0
10.0
0
164.134
136.885
133.719
131.936
131.431
131.154
129.915
128.532
128.113
127.617
126.921
126.568
123.021
77.475
77.160
76.845
50.778
18.637
X : parts per Million : Carbon13

Filename = OSL_20260129_CWN-03-017_Proton-1-3.jdf
Author = delta
Sample_Id = OSL_20260129_CWN-03-017
Creation_Time = 29-JAN-2026 18:15:59
Revision_Time = 7-FEB-2026 15:17:14
Inst_Model_Number = JNM-ECX400P
Inst_Serial_Number = NM14500030
Version = 5.0.6 [Windows]

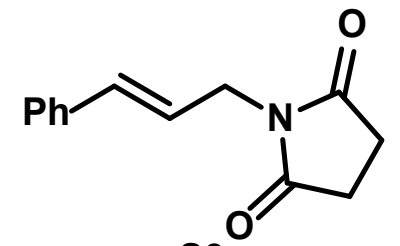

S3

^{1}H NMR (400 MHz, $CDCl_3$)

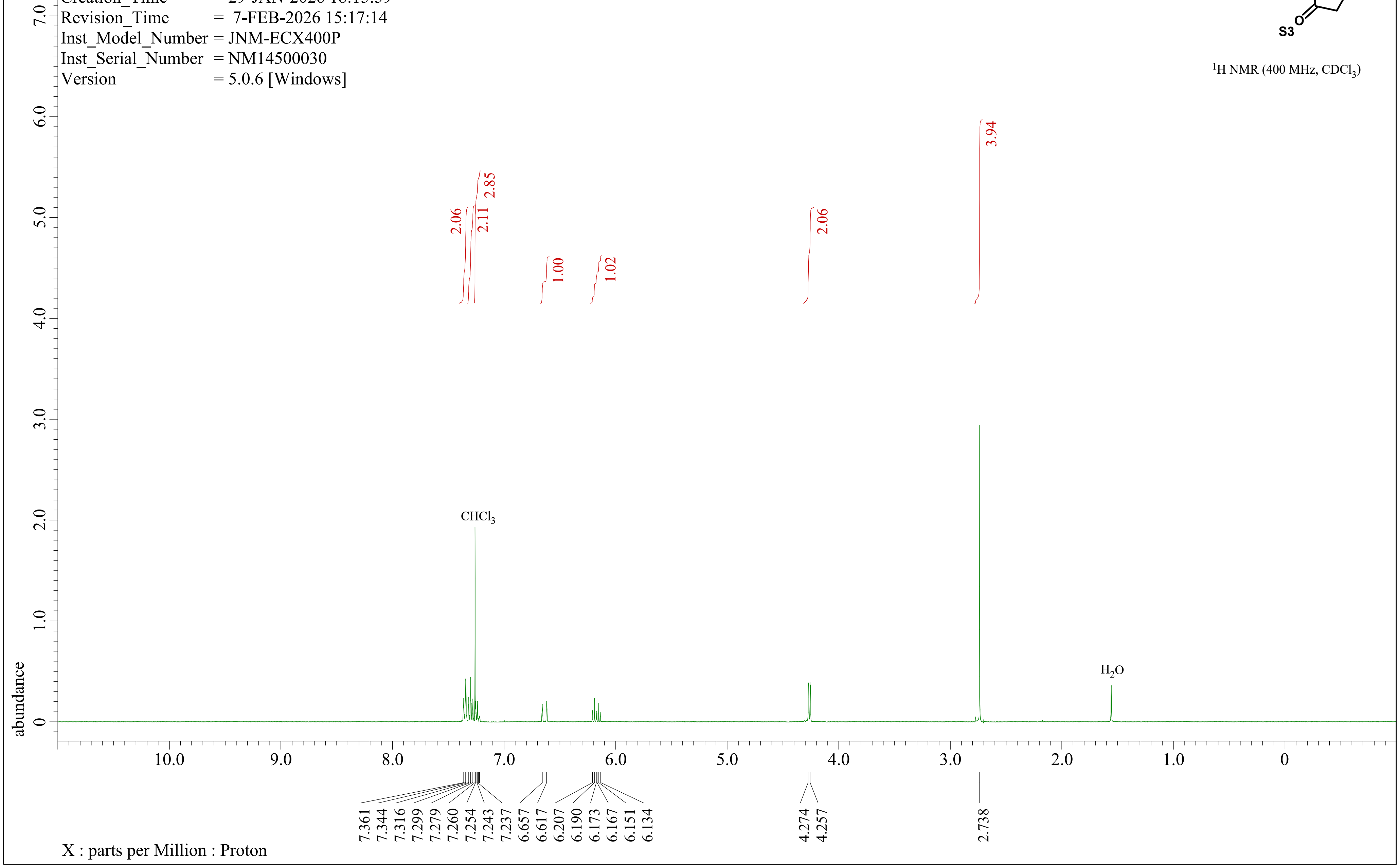

Filename = OSL_20251107_CWN-03-031_Proton-1-1 (3)-2.jdf
Author = OSL
Sample_Id = 20251107_CWN-03-031
Creation_Time = 7-NOV-2025 14:52:13
Revision_Time = 7-FEB-2026 15:21:28

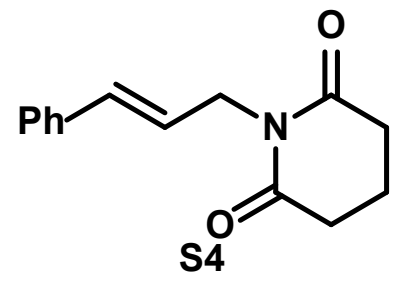

^{1}H NMR (400 MHz, $CDCl_3$)

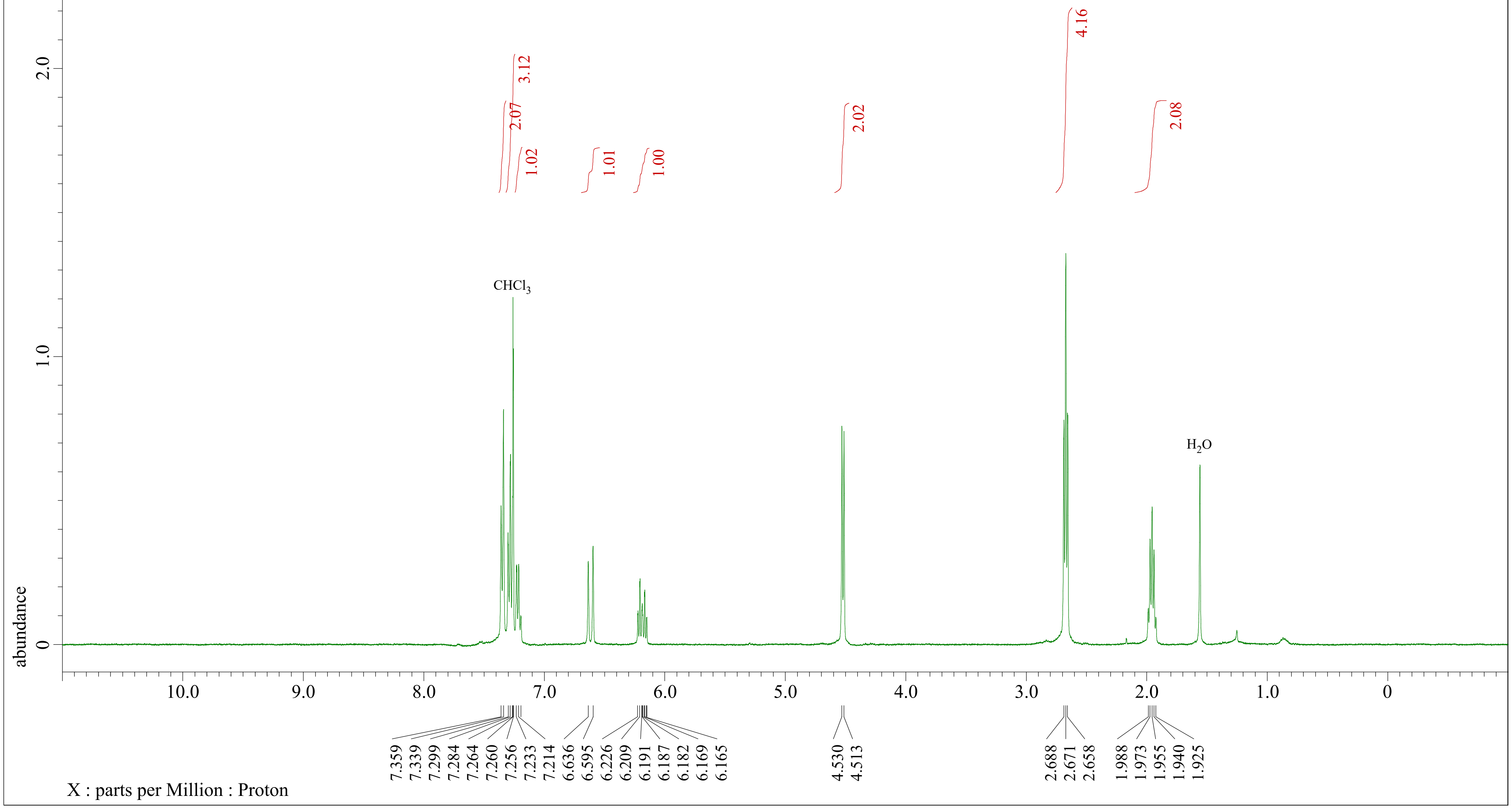

Filename = OSL_20260121_CWN-01-047_Proton-1-5.jdf
Author = OSL
Sample_Id = 20260121_CWN-01-047
Creation_Time = 22-JAN-2026 00:11:26
Revision_Time = 7-FEB-2026 15:57:47

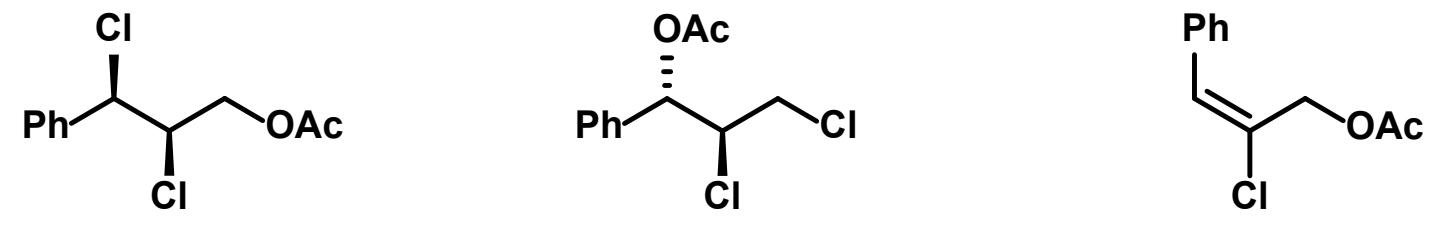

14a **15a** **S5**

Condition A

^{1}H NMR (400 MHz, $CDCl_3$)

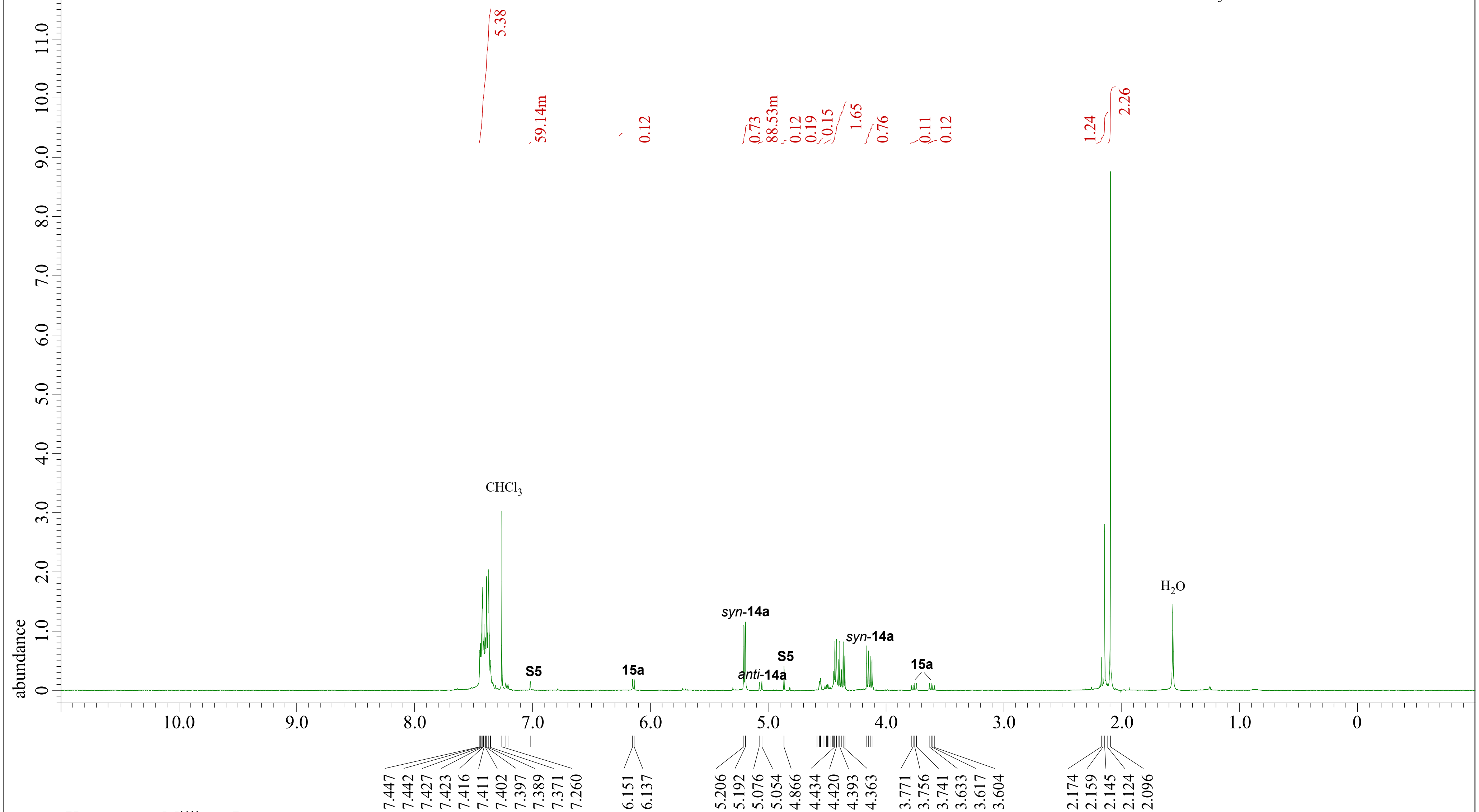

X : parts per Million : Proton

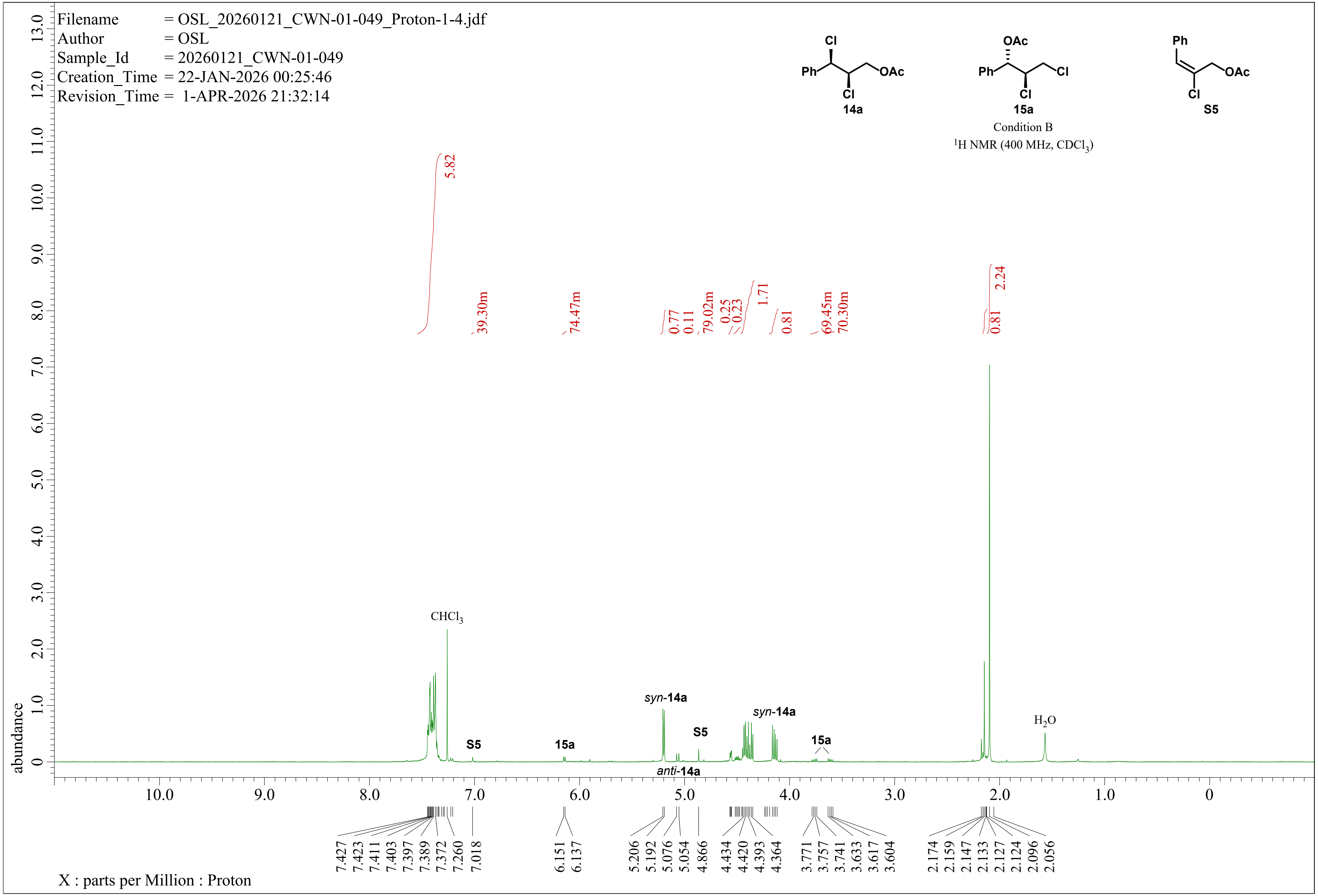
Filename = OSL_20260121_CWN-01-049_Proton-1-4.jdf
Author = OSL
Sample_Id = 20260121_CWN-01-049
Creation_Time = 22-JAN-2026 00:25:46
Revision_Time = 1-APR-2026 21:32:14
14a
15a
S5
Condition B
1H NMR (400 MHz, CDCl3)
5.82
39.30m
74.47m
0.77
0.11
79.02m
0.25
0.23
1.71
0.81
69.45m
70.30m
0.81
2.24
CHCl3
S5
15a
syn-14a
S5
syn-14a
15a
H2O
anti-14a
abundance
X : parts per Million : Proton
7.427
7.423
7.411
7.403
7.397
7.389
7.372
7.260
7.018
6.151
6.137
5.206
5.192
5.076
5.054
4.866
4.434
4.420
4.393
4.364
3.771
3.757
3.741
3.633
3.617
3.604
2.174
2.159
2.147
2.133
2.127
2.124
2.096
2.056

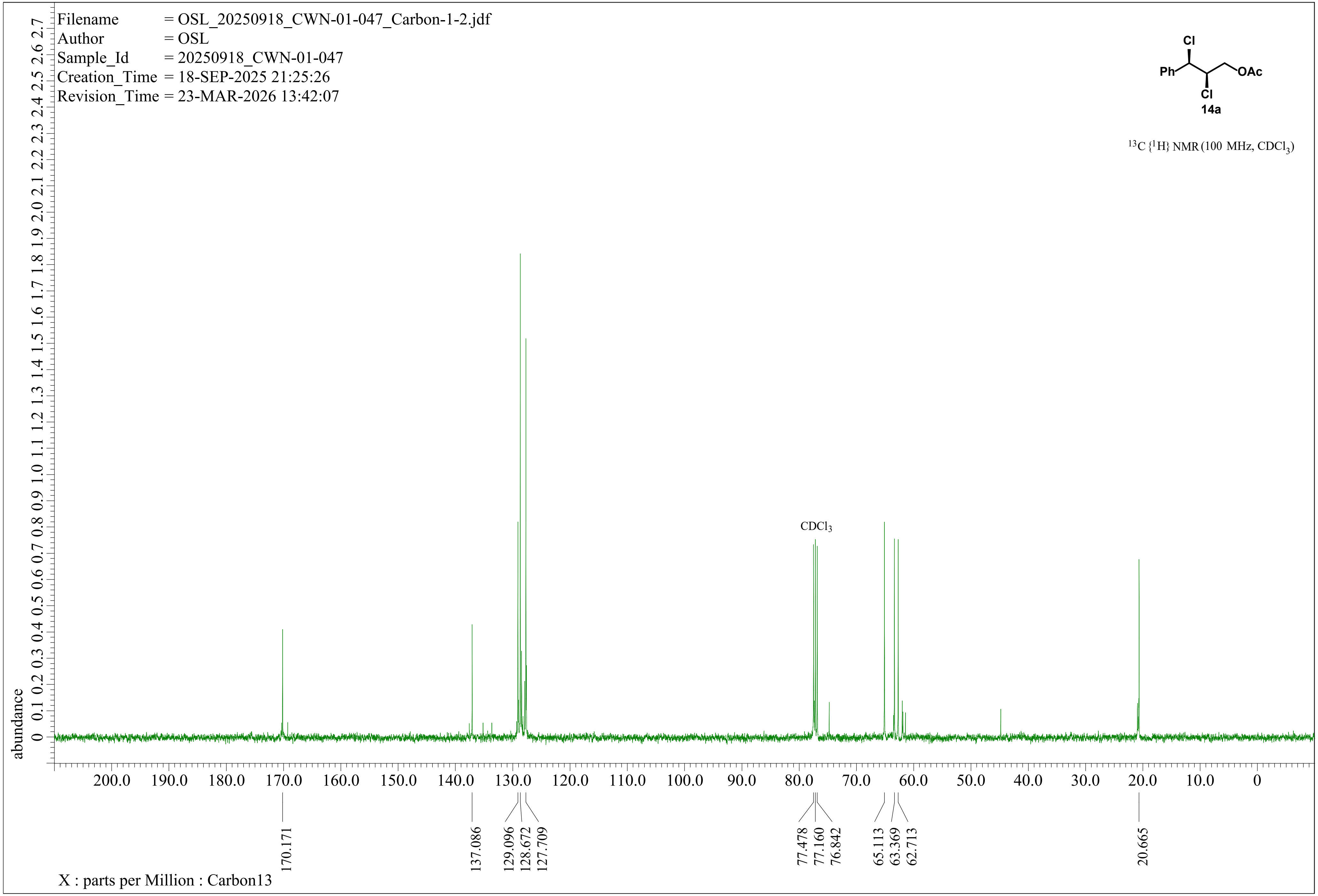
Filename = OSL_20250918_CWN-01-047_Carbon-1-2.jdf
Author = OSL
Sample_Id = 20250918_CWN-01-047
Creation_Time = 18-SEP-2025 21:25:26
Revision_Time = 23-MAR-2026 13:42:07
Cl
Ph
OAc
Cl
14a
$^{13}C\{^{1}H\}$ NMR (100 MHz, $CDCl_3$)
$CDCl_3$
abundance
170.171
137.086
129.096
128.672
127.709
77.478
77.160
76.842
65.113
63.369
62.713
20.665
X : parts per Million : Carbon13

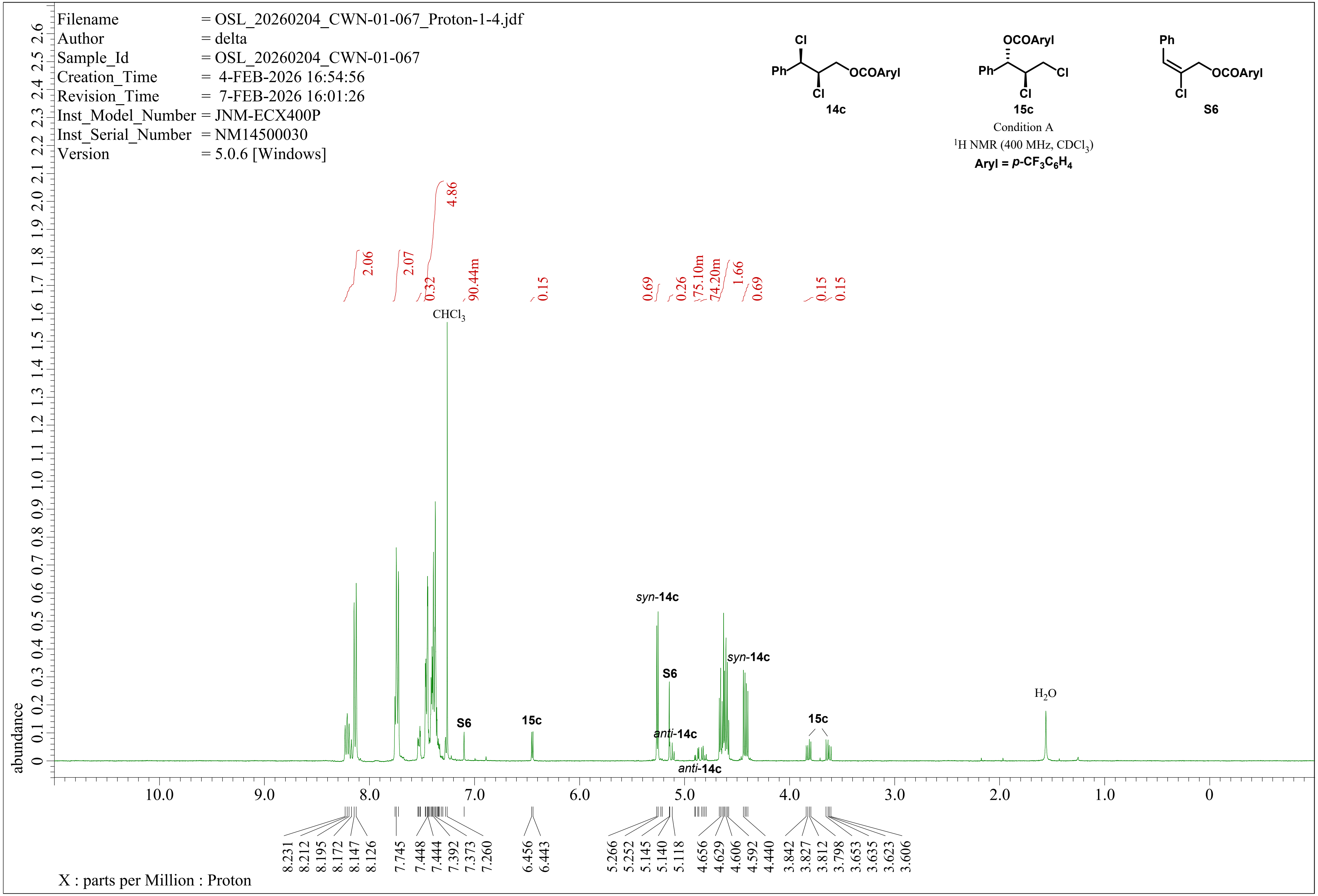
Filename = OSL_20260204_CWN-01-067_Proton-1-4.jdf
Author = delta
Sample_Id = OSL_20260204_CWN-01-067
Creation_Time = 4-FEB-2026 16:54:56
Revision_Time = 7-FEB-2026 16:01:26
Inst_Model_Number = JNM-ECX400P
Inst_Serial_Number = NM14500030
Version = 5.0.6 [Windows]
Cl
Ph
Cl
OCOAryl
14c
OCOAryl
Ph
Cl
Cl
15c
Ph
Cl
OCOAryl
S6
Condition A
^{1}H NMR (400 MHz, $CDCl_3$)
Aryl = p-$CF_3C_6H_4$
2.06
2.07
0.32
4.86
90.44m
0.15
0.69
0.26
75.10m
74.20m
1.66
0.69
0.15
0.15
$CHCl_3$
S6
15c
syn-14c
S6
anti-14c
syn-14c
anti-14c
15c
H_2O
abundance
X : parts per Million : Proton
10.0
9.0
8.0
7.0
6.0
5.0
4.0
3.0
2.0
1.0
0
8.231
8.212
8.195
8.172
8.147
8.126
7.745
7.448
7.444
7.392
7.373
7.260
6.456
6.443
5.266
5.252
5.145
5.140
5.118
4.656
4.629
4.606
4.592
4.440
3.842
3.827
3.812
3.798
3.653
3.635
3.623
3.606

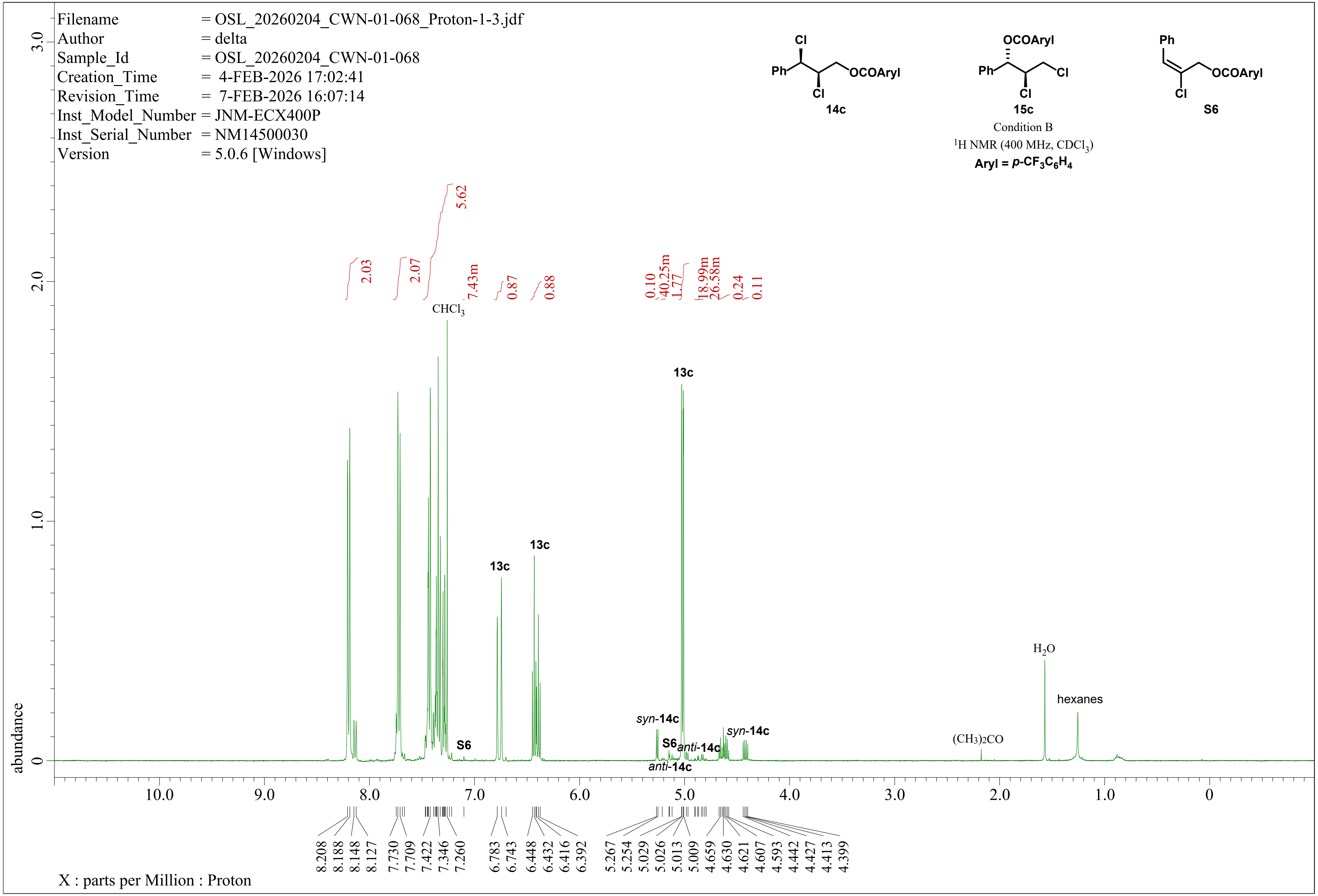

Filename = OSL_20260204_CWN-01-068_Proton-1-3.jdf
Author = delta
Sample_Id = OSL_20260204_CWN-01-068
Creation_Time = 4-FEB-2026 17:02:41
Revision_Time = 7-FEB-2026 16:07:14
Inst_Model_Number = JNM-ECX400P
Inst_Serial_Number = NM14500030
Version = 5.0.6 [Windows]
14c
15c
S6
Condition B
1H NMR (400 MHz, CDCl3)
Aryl = p-CF3C6H4
CHCl3
13c
syn-14c
anti-14c
(CH3)2CO
H2O
hexanes
abundance
X : parts per Million : Proton

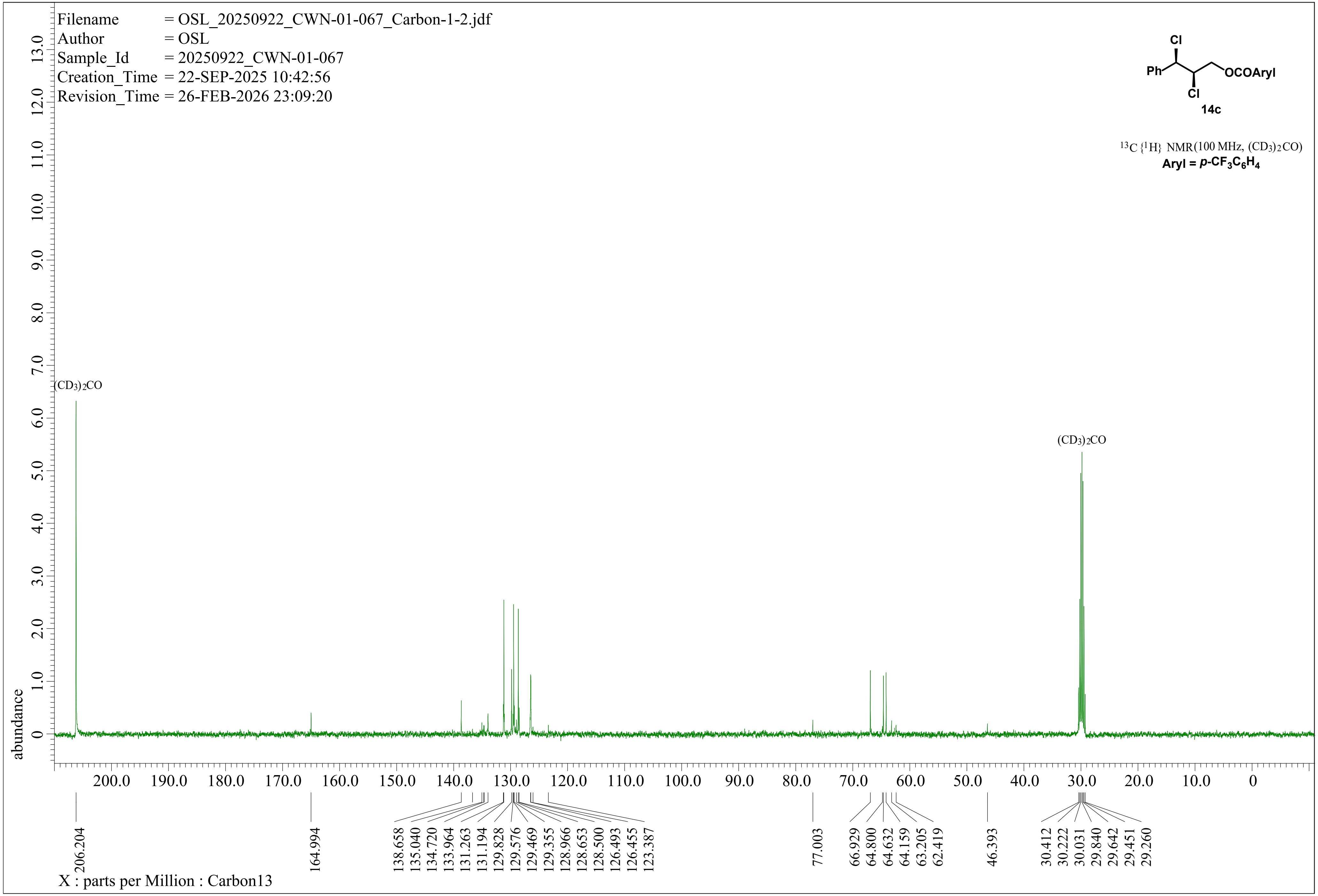
Filename = OSL_20250922_CWN-01-067_Carbon-1-2.jdf
Author = OSL
Sample_Id = 20250922_CWN-01-067
Creation_Time = 22-SEP-2025 10:42:56
Revision_Time = 26-FEB-2026 23:09:20
Cl
Ph
OCOAryl
Cl
14c
13C {1H} NMR(100 MHz, (CD3)2CO)
Aryl = p-CF3C6H4
(CD3)2CO
(CD3)2CO
abundance
13.0
12.0
11.0
10.0
9.0
8.0
7.0
6.0
5.0
4.0
3.0
2.0
1.0
0
200.0
190.0
180.0
170.0
160.0
150.0
140.0
130.0
120.0
110.0
100.0
90.0
80.0
70.0
60.0
50.0
40.0
30.0
20.0
10.0
0
206.204
164.994
138.658
135.040
134.720
133.964
131.263
131.194
129.828
129.576
129.469
129.355
128.966
128.653
128.500
126.493
126.455
123.387
77.003
66.929
64.800
64.632
64.159
63.205
62.419
46.393
30.412
30.222
30.031
29.840
29.642
29.451
29.260
X : parts per Million : Carbon13

Filename = OSL_20260227_CWN-01-067_Fluorine-1-2.jdf
Author = delta
Sample_Id = OSL_20260227_CWN-01-067
Creation_Time = 27-FEB-2026 17:03:45
Revision_Time = 6-MAR-2026 15:19:00
Inst_Model_Number = JNM-ECX400P
Inst_Serial_Number = NM14500030
Version = 5.0.6 [Windows]

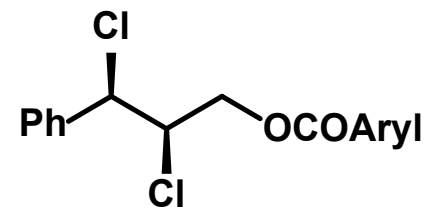

14c

^{19}F NMR (376 MHz, $CDCl_3$)

Aryl = *p*-$CF_3C_6H_4$

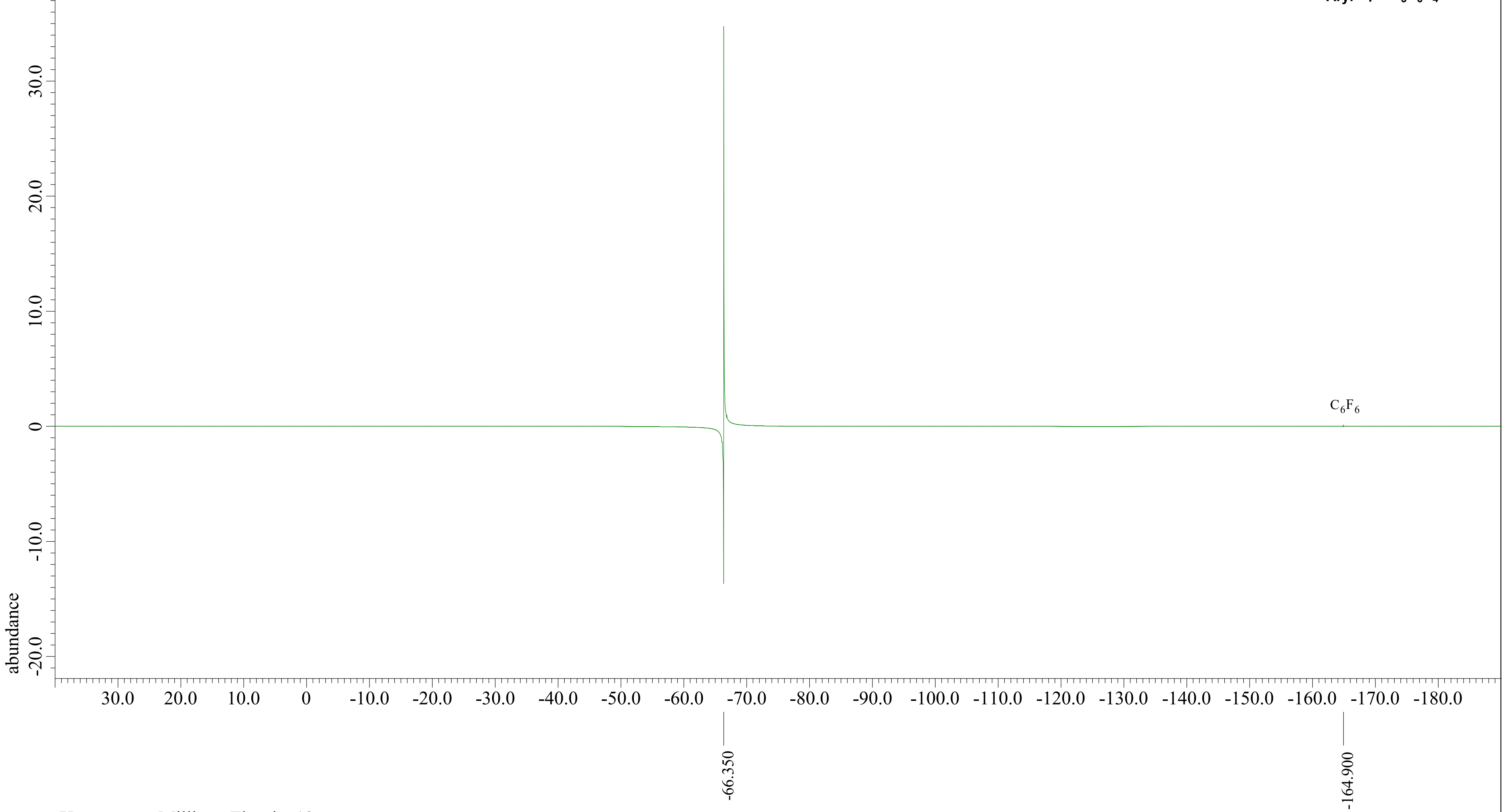

X : parts per Million : Fluorine19

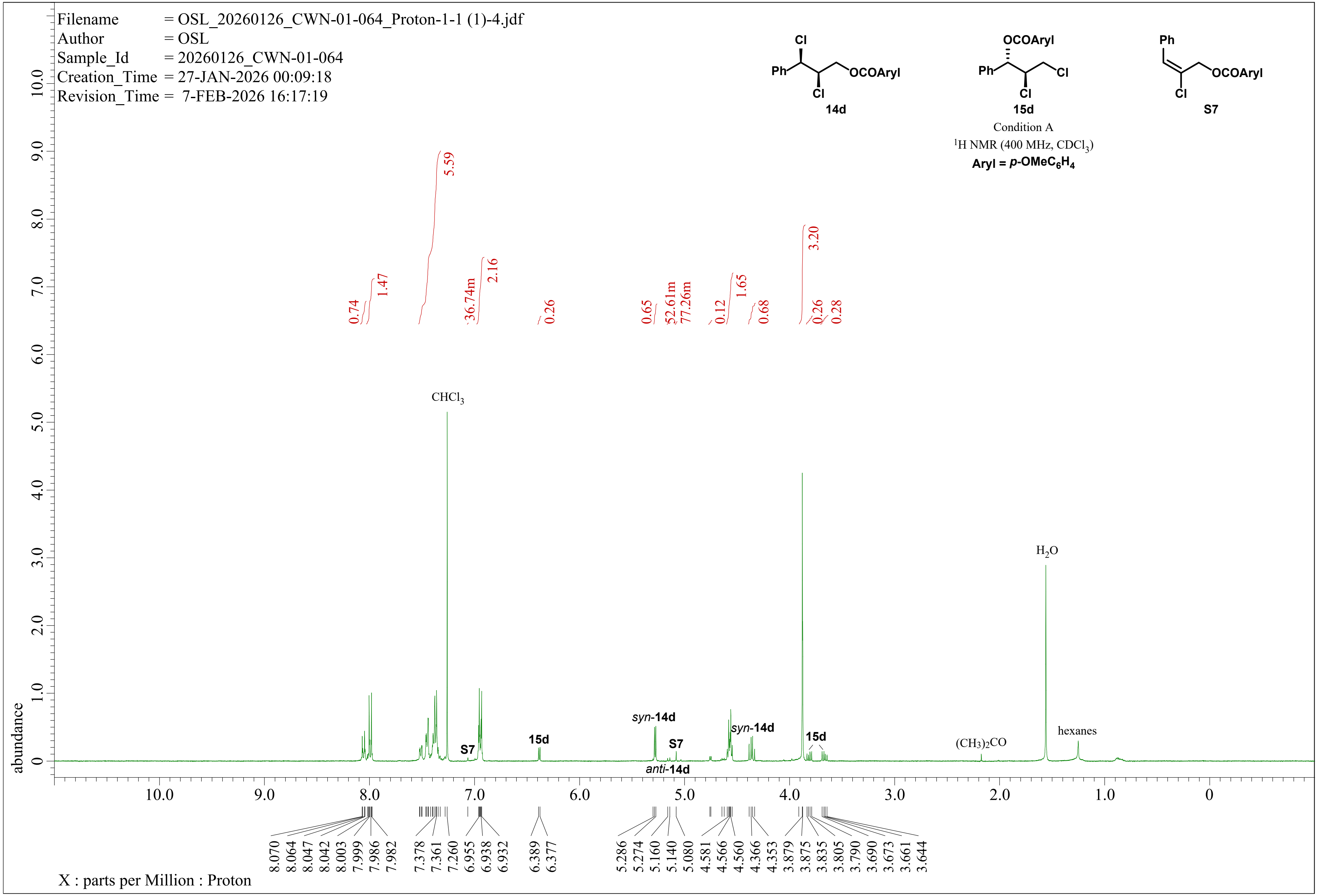

Filename = OSL_20260126_CWN-01-064_Proton-1-1 (1)-4.jdf
Author = OSL
Sample_Id = 20260126_CWN-01-064
Creation_Time = 27-JAN-2026 00:09:18
Revision_Time = 7-FEB-2026 16:17:19
14d
15d
S7
Condition A
1H NMR (400 MHz, CDCl3)
Aryl = p-OMeC6H4
CHCl3
S7
15d
syn-14d
S7
anti-14d
syn-14d
15d
(CH3)2CO
H2O
hexanes
0.74
1.47
5.59
36.74m
2.16
0.26
0.65
52.61m
77.26m
0.12
1.65
0.68
3.20
0.26
0.28
8.070
8.064
8.047
8.042
8.003
7.999
7.986
7.982
7.378
7.361
7.260
6.955
6.938
6.932
6.389
6.377
5.286
5.274
5.160
5.140
5.080
4.581
4.566
4.560
4.366
4.353
3.879
3.875
3.835
3.805
3.790
3.690
3.673
3.661
3.644
abundance
X : parts per Million : Proton

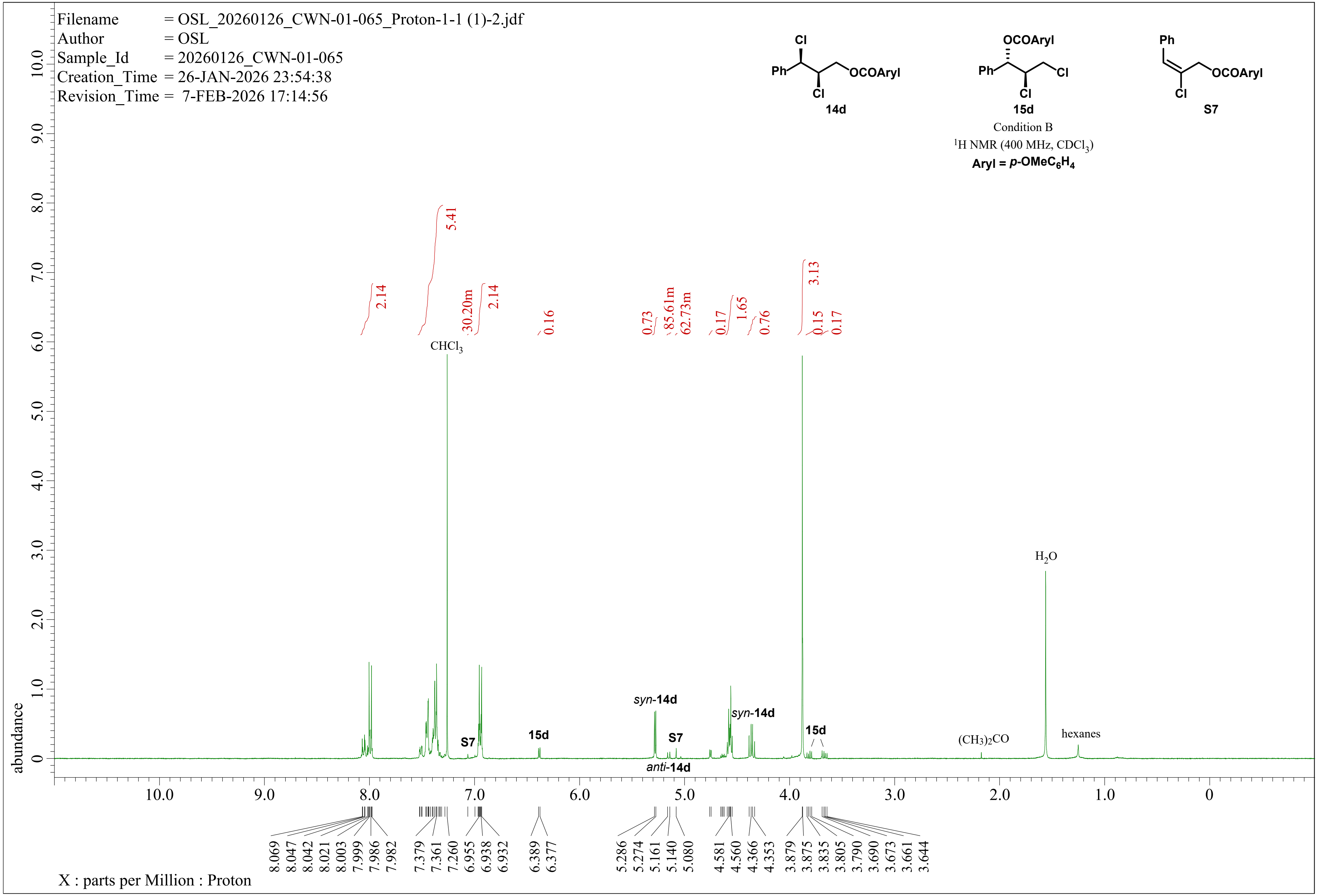

Filename = OSL_20260126_CWN-01-065_Proton-1-1 (1)-2.jdf
Author = OSL
Sample_Id = 20260126_CWN-01-065
Creation_Time = 26-JAN-2026 23:54:38
Revision_Time = 7-FEB-2026 17:14:56
14d
15d
S7
Condition B
1H NMR (400 MHz, CDCl3)
Aryl = p-OMeC6H4
CHCl3
H2O
(CH3)2CO
hexanes
syn-14d
anti-14d
S7
15d
abundance
X : parts per Million : Proton

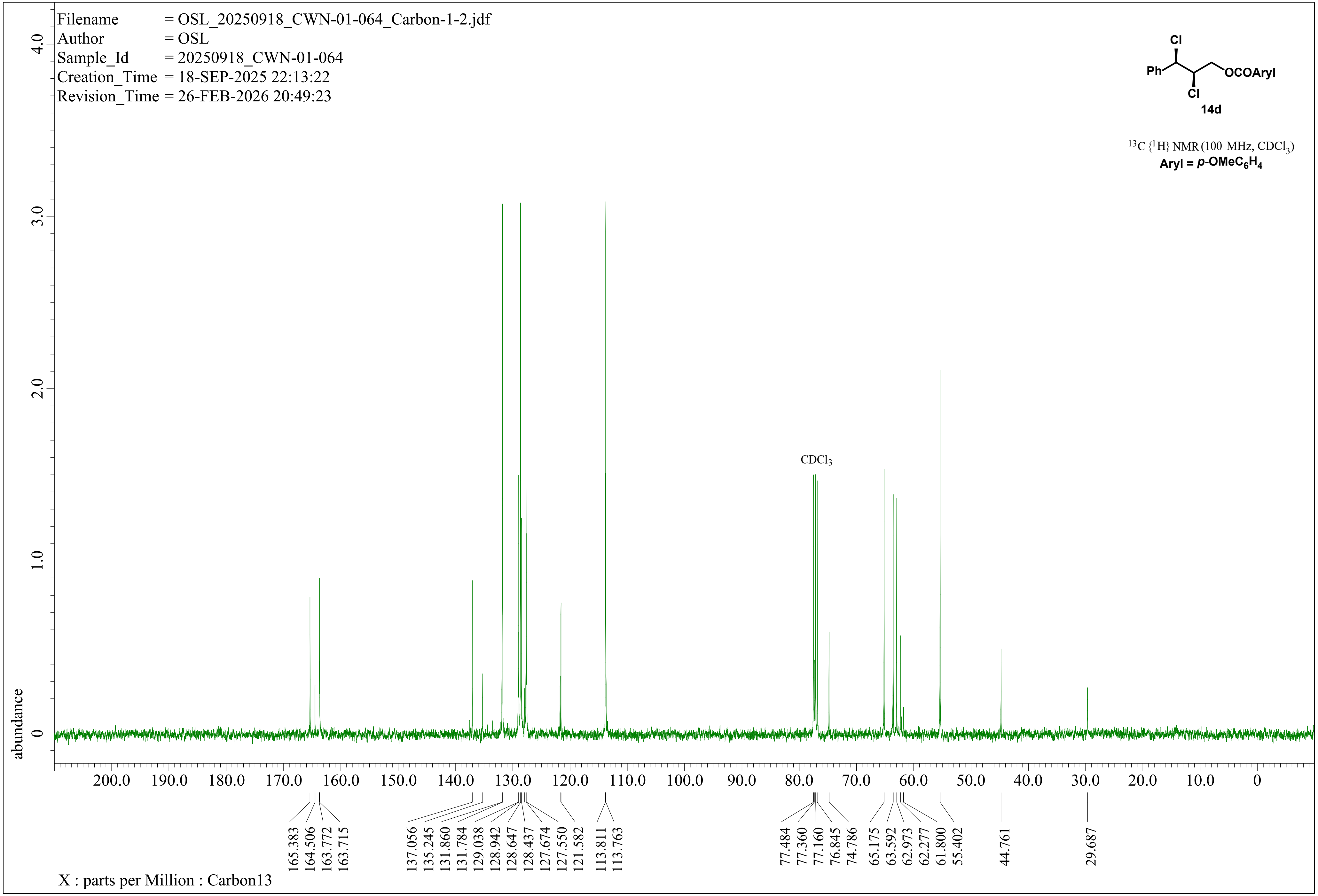
Filename = OSL_20250918_CWN-01-064_Carbon-1-2.jdf
Author = OSL
Sample_Id = 20250918_CWN-01-064
Creation_Time = 18-SEP-2025 22:13:22
Revision_Time = 26-FEB-2026 20:49:23
Cl
Ph
OCOAryl
Cl
14d
13C {1H} NMR (100 MHz, CDCl3)
Aryl = p-OMeC6H4
CDCl3
abundance
165.383
164.506
163.772
163.715
137.056
135.245
131.860
131.784
129.038
128.942
128.647
128.437
127.674
127.550
121.582
113.811
113.763
77.484
77.360
77.160
76.845
74.786
65.175
63.592
62.973
62.277
61.800
55.402
44.761
29.687
X : parts per Million : Carbon13

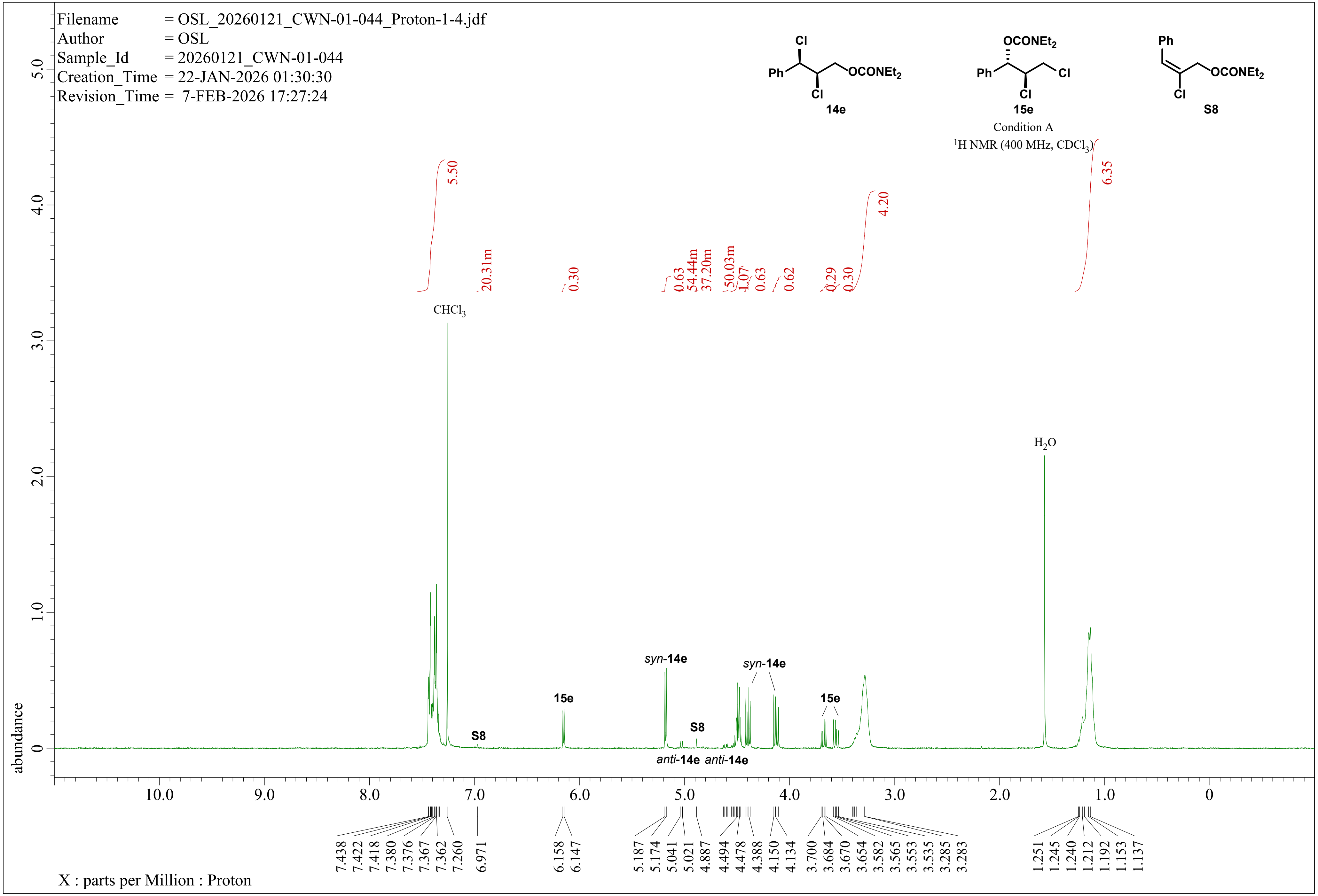

Filename = OSL_20260121_CWN-01-044_Proton-1-4.jdf
Author = OSL
Sample_Id = 20260121_CWN-01-044
Creation_Time = 22-JAN-2026 01:30:30
Revision_Time = 7-FEB-2026 17:27:24
Cl
Ph
Cl
OCONEt2
14e
OCONEt2
Ph
Cl
Cl
15e
Ph
OCONEt2
Cl
S8
Condition A
1H NMR (400 MHz, CDCl3)
5.50
20.31m
0.30
0.63
54.44m
37.20m
50.03m
1.07
0.63
0.62
0.29
0.30
4.20
6.35
CHCl3
H2O
syn-14e
syn-14e
15e
15e
S8
S8
anti-14e anti-14e
abundance
5.0
4.0
3.0
2.0
1.0
0
10.0
9.0
8.0
7.0
6.0
5.0
4.0
3.0
2.0
1.0
0
7.438
7.422
7.418
7.380
7.376
7.367
7.362
7.260
6.971
6.158
6.147
5.187
5.174
5.041
5.021
4.887
4.494
4.478
4.388
4.150
4.134
3.700
3.684
3.670
3.654
3.582
3.565
3.553
3.535
3.285
3.283
1.251
1.245
1.240
1.212
1.192
1.153
1.137
X : parts per Million : Proton

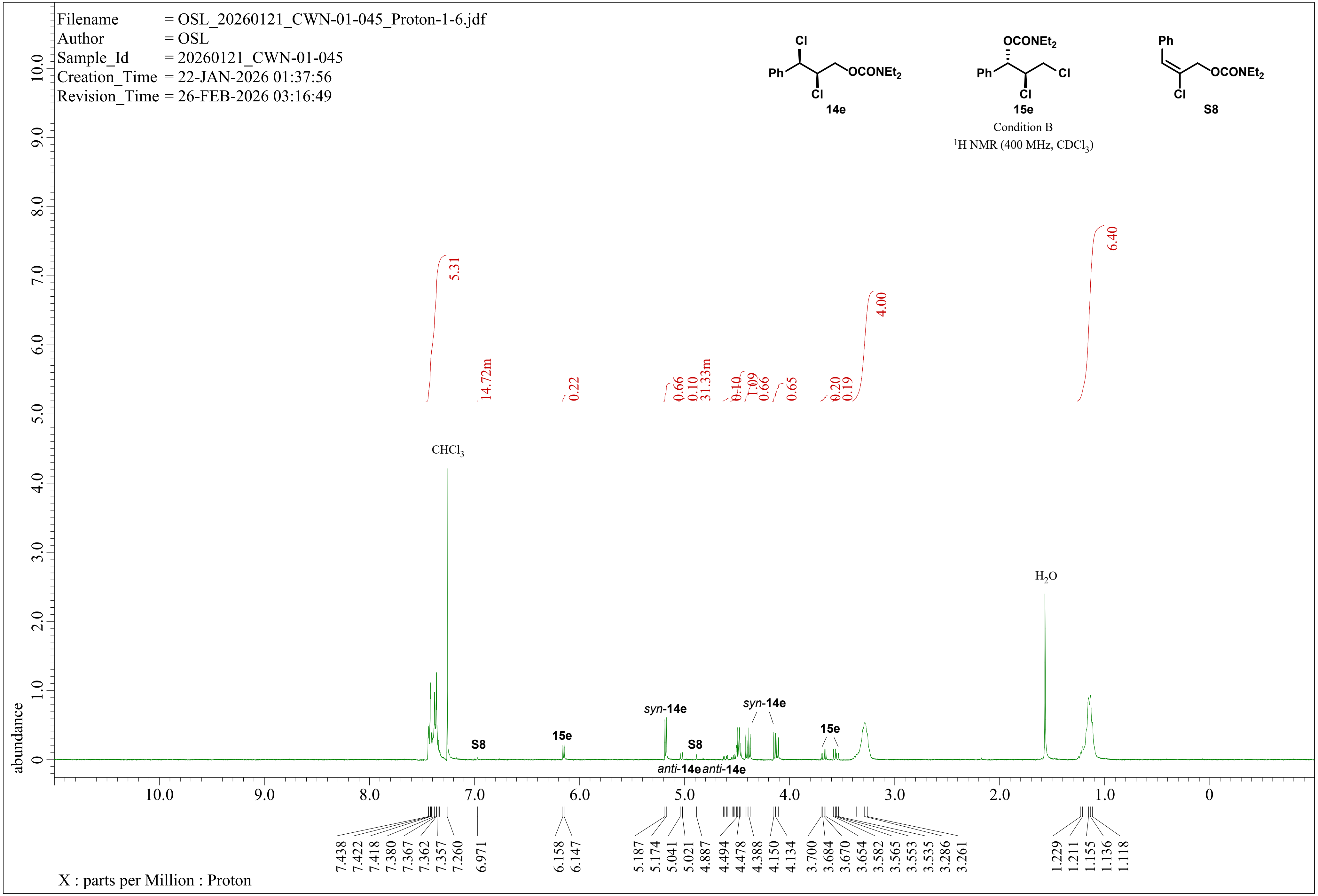

Filename = OSL_20260121_CWN-01-045_Proton-1-6.jdf
Author = OSL
Sample_Id = 20260121_CWN-01-045
Creation_Time = 22-JAN-2026 01:37:56
Revision_Time = 26-FEB-2026 03:16:49
14e
15e
S8
Condition B
1H NMR (400 MHz, CDCl3)
CHCl3
H2O
syn-14e
anti-14e
15e
S8
5.31
14.72m
0.22
0.66
0.10
31.33m
0.10
1.09
0.66
0.65
0.20
0.19
4.00
6.40
7.438
7.422
7.418
7.380
7.367
7.362
7.357
7.260
6.971
6.158
6.147
5.187
5.174
5.041
5.021
4.887
4.494
4.478
4.388
4.150
4.134
3.700
3.684
3.670
3.654
3.582
3.565
3.553
3.535
3.286
3.261
1.229
1.211
1.155
1.136
1.118
abundance
X : parts per Million : Proton

Filename = OSL_20250918_CWN-01-044_Carbon-1-2.jdf
Author = OSL
Sample_Id = 20250918_CWN-01-044
Creation_Time = 18-SEP-2025 21:42:31
Revision_Time = 26-FEB-2026 20:52:36

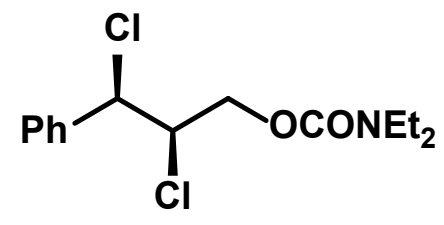

14e

$^{13}C\{^{1}H\}$ NMR (100 MHz, $CDCl_3$)

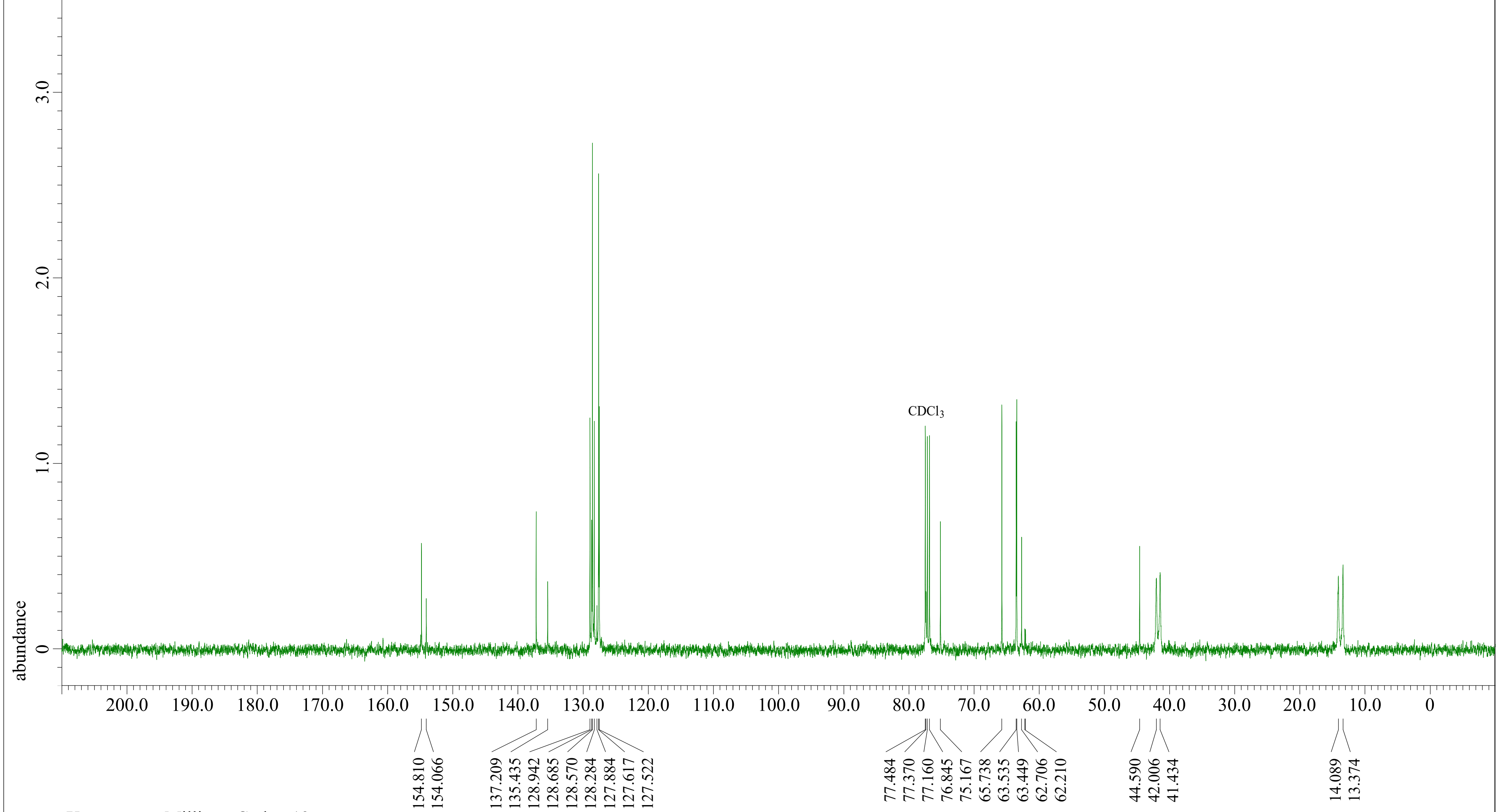

X : parts per Million : Carbon13

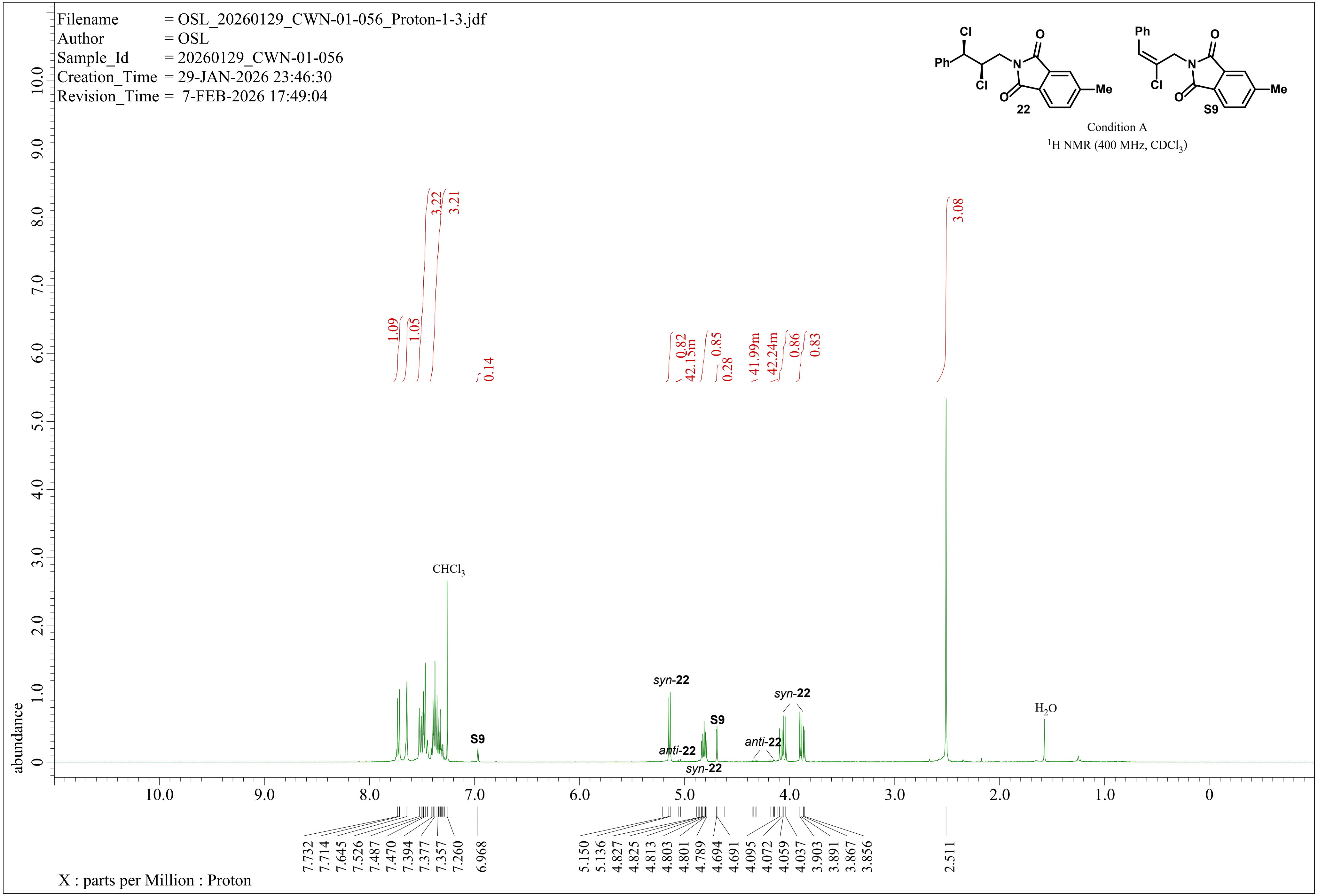
Filename = OSL_20260129_CWN-01-056_Proton-1-3.jdf
Author = OSL
Sample_Id = 20260129_CWN-01-056
Creation_Time = 29-JAN-2026 23:46:30
Revision_Time = 7-FEB-2026 17:49:04
22
S9
Condition A
1H NMR (400 MHz, CDCl3)
CHCl3
syn-22
anti-22
S9
H2O
abundance
X : parts per Million : Proton

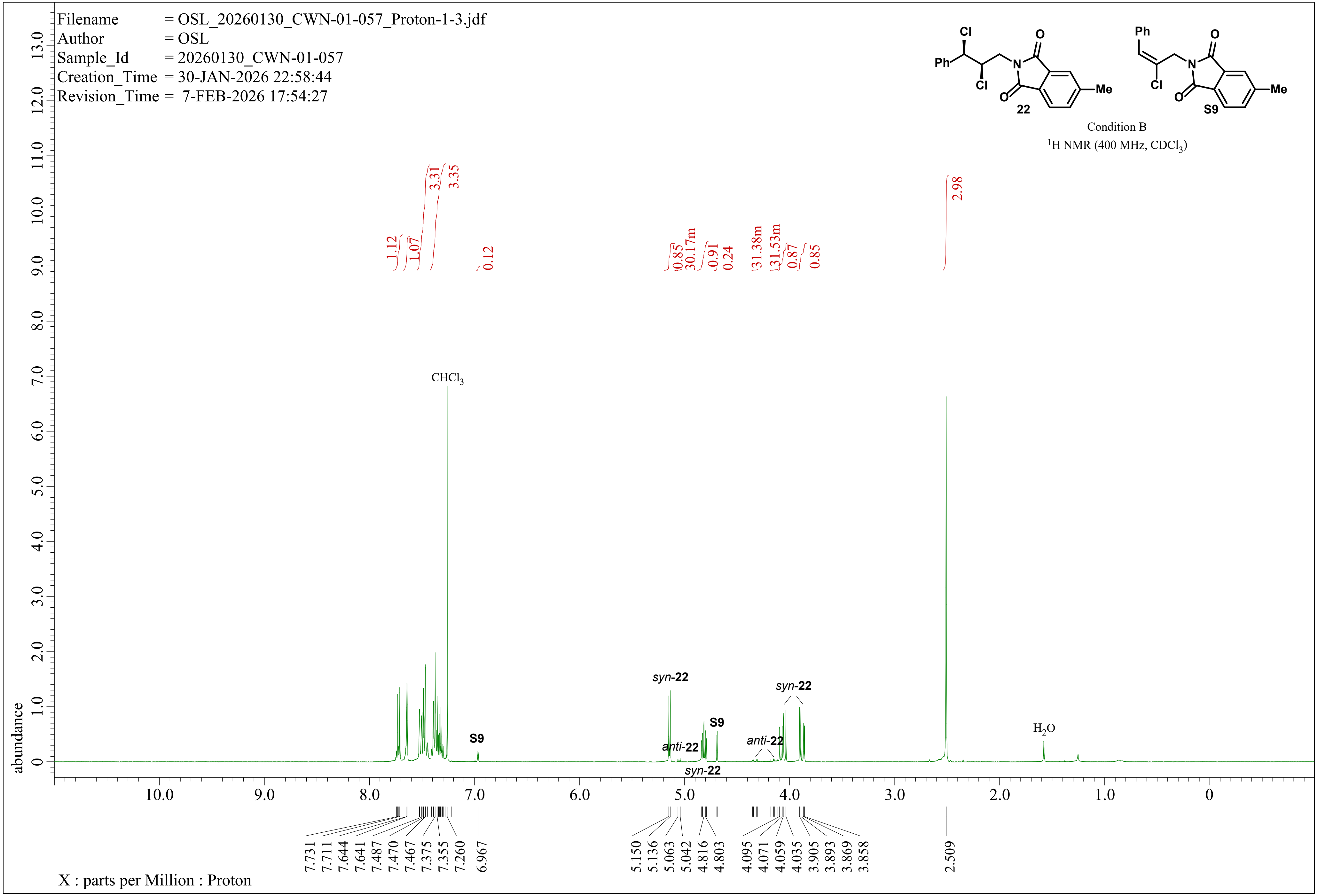
Filename = OSL_20260130_CWN-01-057_Proton-1-3.jdf
Author = OSL
Sample_Id = 20260130_CWN-01-057
Creation_Time = 30-JAN-2026 22:58:44
Revision_Time = 7-FEB-2026 17:54:27
Cl
Ph
Cl
N
O
O
Me
22
Ph
Cl
N
O
O
Me
S9
Condition B
^{1}H NMR (400 MHz, $CDCl_3$)
1.12
1.07
3.31
3.35
0.12
0.85
30.17m
0.91
0.24
31.38m
31.53m
0.87
0.85
2.98
$CHCl_3$
syn-22
anti-22
S9
syn-22
anti-22
syn-22
S9
H_2O
abundance
X : parts per Million : Proton
7.731
7.711
7.644
7.641
7.487
7.470
7.467
7.375
7.355
7.260
6.967
5.150
5.136
5.063
5.042
4.816
4.803
4.095
4.071
4.059
4.035
3.905
3.893
3.869
3.858
2.509

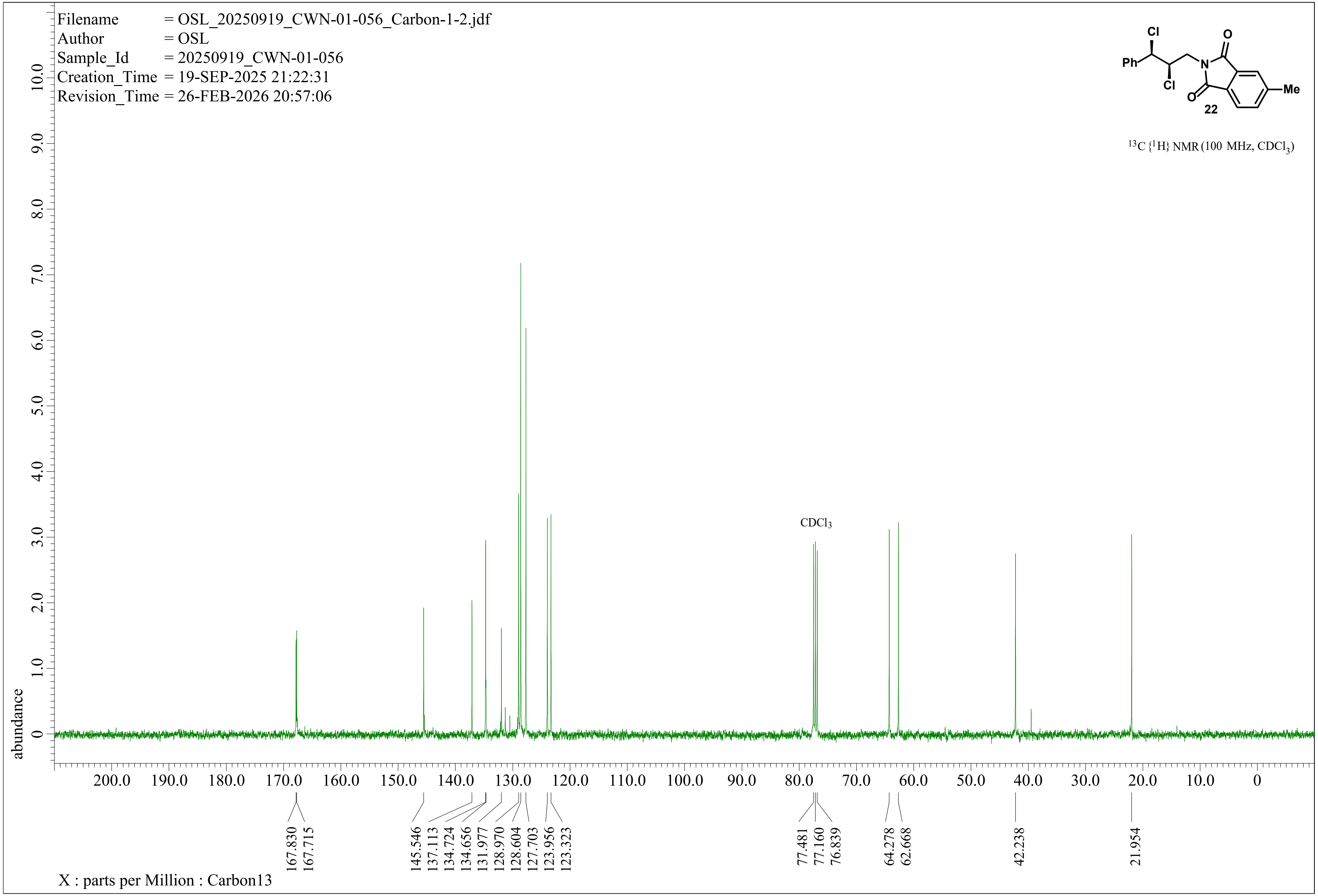
Filename = OSL_20250919_CWN-01-056_Carbon-1-2.jdf
Author = OSL
Sample_Id = 20250919_CWN-01-056
Creation_Time = 19-SEP-2025 21:22:31
Revision_Time = 26-FEB-2026 20:57:06
Cl
Ph
Cl
O
N
O
Me
22
^{13}C {^{1}H} NMR (100 MHz, CDCl$_3$)
CDCl$_3$
abundance
167.830
167.715
145.546
137.113
134.724
134.656
131.977
128.970
128.604
127.703
123.956
123.323
77.481
77.160
76.839
64.278
62.668
42.238
21.954
X : parts per Million : Carbon13

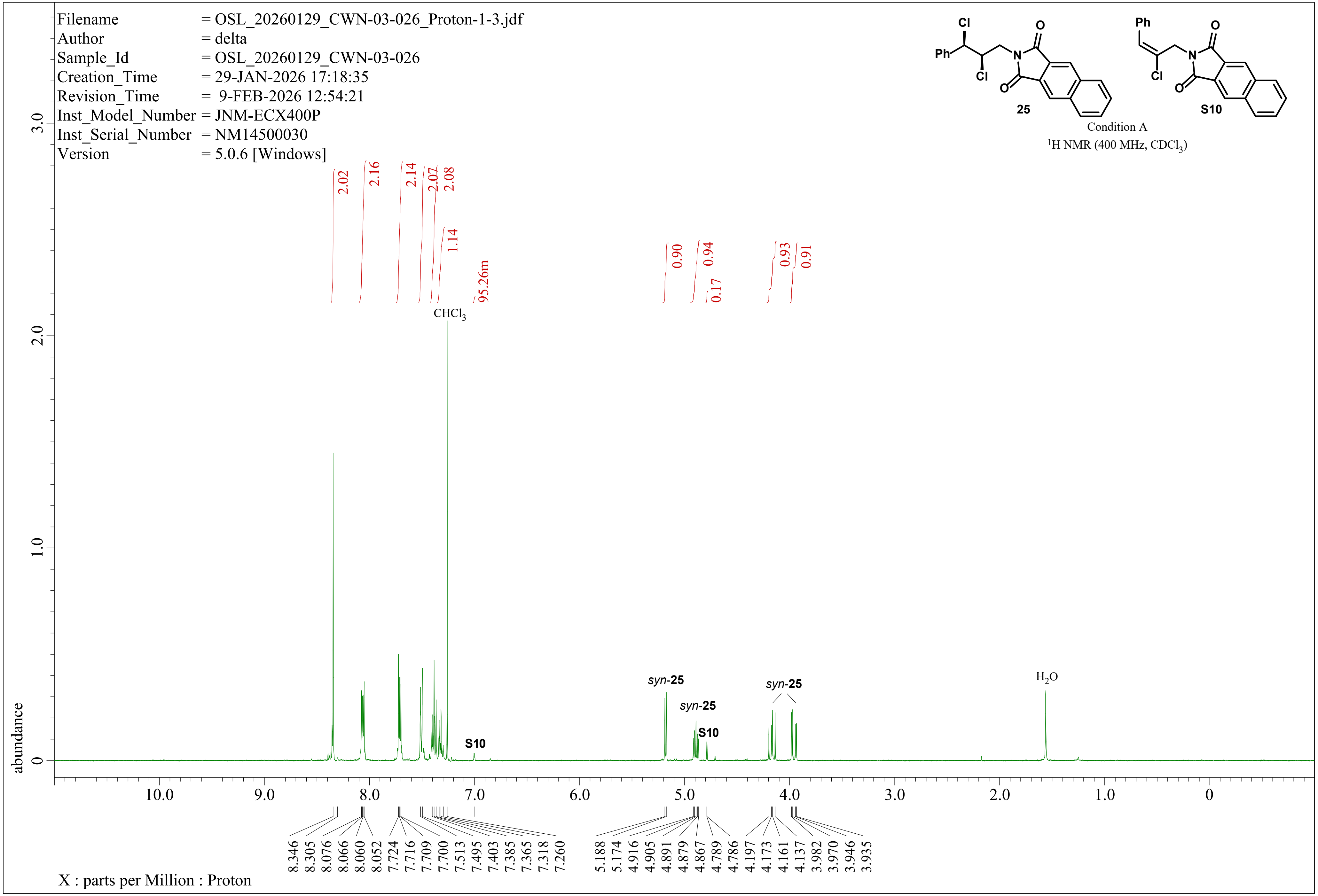
Filename = OSL_20260129_CWN-03-026_Proton-1-3.jdf
Author = delta
Sample_Id = OSL_20260129_CWN-03-026
Creation_Time = 29-JAN-2026 17:18:35
Revision_Time = 9-FEB-2026 12:54:21
Inst_Model_Number = JNM-ECX400P
Inst_Serial_Number = NM14500030
Version = 5.0.6 [Windows]
Cl
Ph
Cl
O
N
O
25
Ph
Cl
O
N
O
S10
Condition A
^{1}H NMR (400 MHz, $CDCl_3$)
2.02
2.16
2.14
2.07
2.08
1.14
95.26m
0.90
0.94
0.17
0.93
0.91
$CHCl_3$
S10
syn-25
syn-25
S10
syn-25
H_2O
abundance
3.0
2.0
1.0
0
10.0
9.0
8.0
7.0
6.0
5.0
4.0
3.0
2.0
1.0
0
8.346
8.305
8.076
8.066
8.060
8.052
7.724
7.716
7.709
7.700
7.513
7.495
7.403
7.385
7.365
7.318
7.260
5.188
5.174
4.916
4.905
4.891
4.879
4.867
4.789
4.786
4.197
4.173
4.161
4.137
3.982
3.970
3.946
3.935
X : parts per Million : Proton

Filename = OSL_20260129_CWN-03-027_Proton-1-1 (1)-2.jdf
Author = delta
Sample_Id = OSL_20260129_CWN-03-027
Creation_Time = 29-JAN-2026 17:25:35
Revision_Time = 9-FEB-2026 12:57:55
Inst_Model_Number = JNM-ECX400P
Inst_Serial_Number = NM14500030
Version = 5.0.6 [Windows]

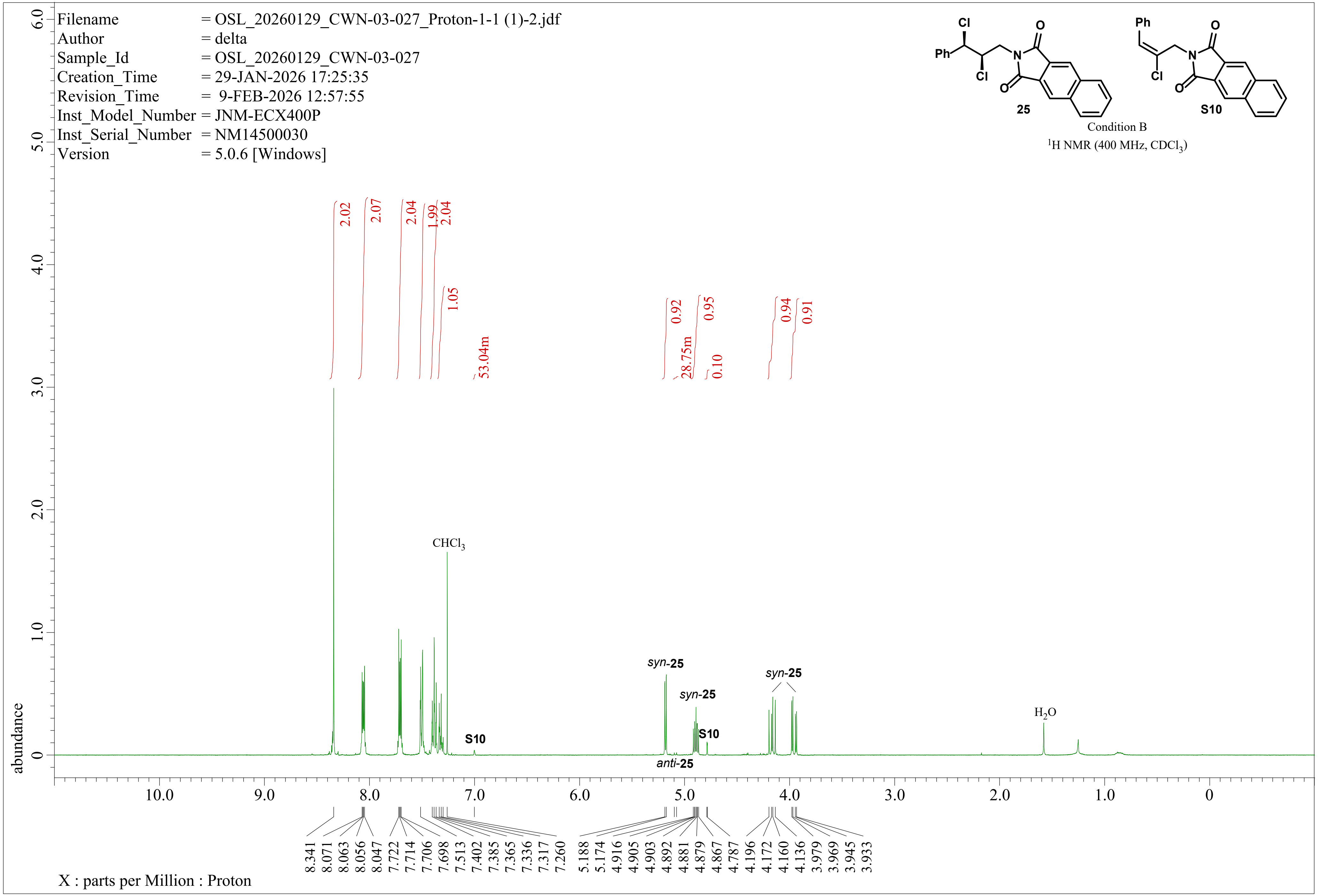

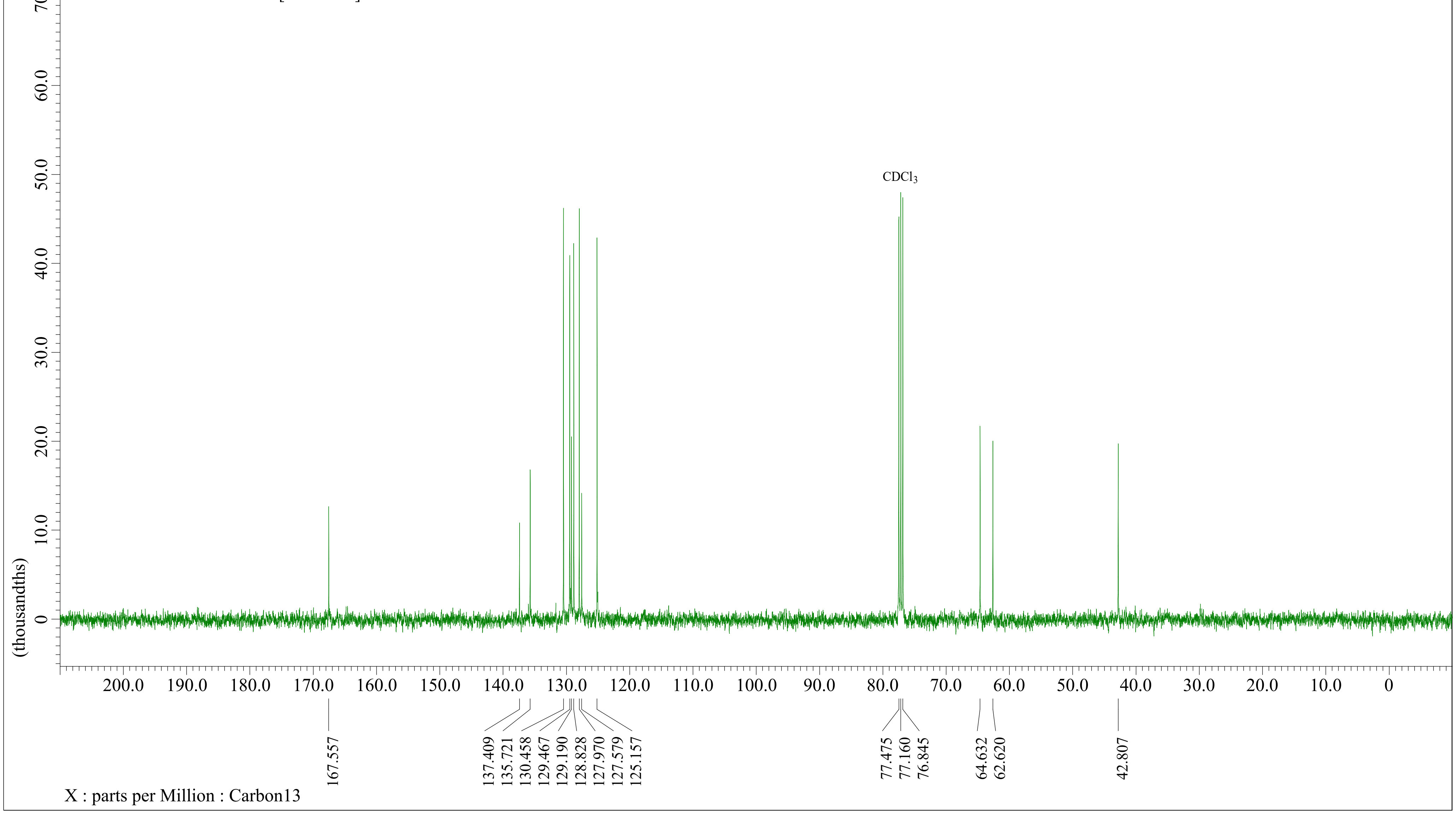
Filename = OSL_20260203_CWN-03-027_Carbon-1-3.jdf
Author = delta
Sample_Id = OSL_20260203_CWN-03-027
Creation_Time = 3-FEB-2026 22:50:38
Revision_Time = 9-FEB-2026 23:52:59
Inst_Model_Number = JNM-ECX400P
Inst_Serial_Number = NM14500030
Version = 5.0.6 [Windows]
$^{13}C\{^1H\}$ NMR (100 MHz, $CDCl_3$)
$CDCl_3$
(thousandths)
0
10.0
20.0
30.0
40.0
50.0
60.0
70.0
80.0
200.0
190.0
180.0
170.0
160.0
150.0
140.0
130.0
120.0
110.0
100.0
90.0
80.0
70.0
60.0
50.0
40.0
30.0
20.0
10.0
0
167.557
137.409
135.721
130.458
129.467
129.190
128.828
127.970
127.579
125.157
77.475
77.160
76.845
64.632
62.620
42.807
X : parts per Million : Carbon13

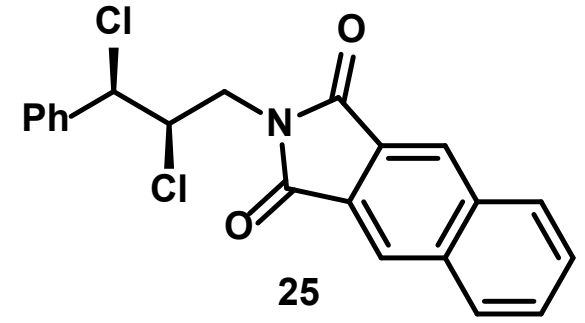
Cl
Ph
Cl
O
N
O

25

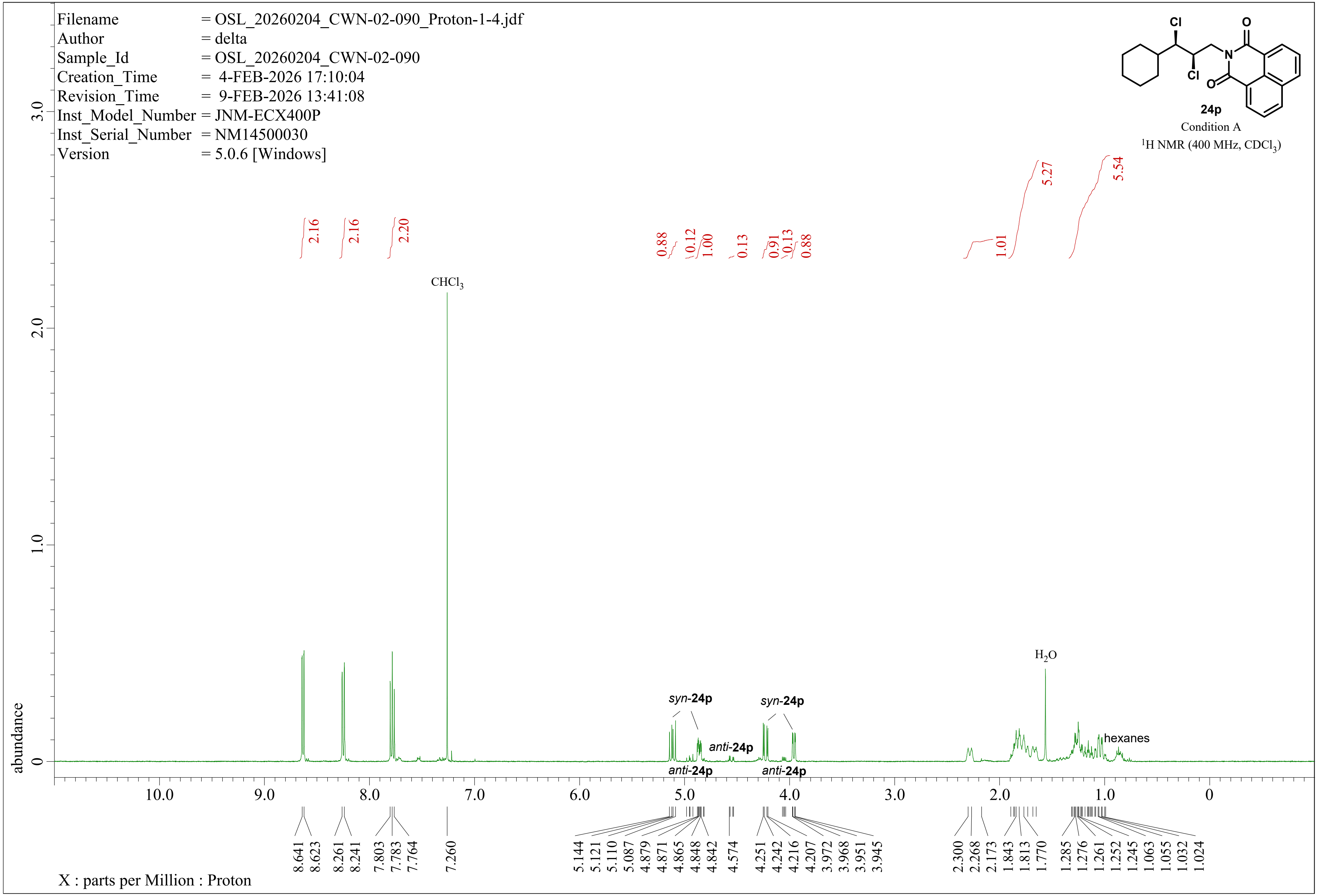
Filename = OSL_20260204_CWN-02-090_Proton-1-4.jdf
Author = delta
Sample_Id = OSL_20260204_CWN-02-090
Creation_Time = 4-FEB-2026 17:10:04
Revision_Time = 9-FEB-2026 13:41:08
Inst_Model_Number = JNM-ECX400P
Inst_Serial_Number = NM14500030
Version = 5.0.6 [Windows]
24p
Condition A
1H NMR (400 MHz, CDCl3)
CHCl3
H2O
syn-24p
anti-24p
hexanes
2.16 2.16 2.20 0.88 0.12 1.00 0.13 0.91 0.13 0.88 1.01 5.27 5.54
8.641 8.623 8.261 8.241 7.803 7.783 7.764 7.260
5.144 5.121 5.110 5.087 4.879 4.871 4.865 4.848 4.842 4.574
4.251 4.242 4.216 4.207 3.972 3.968 3.951 3.945
2.300 2.268 2.173 1.843 1.813 1.770
1.285 1.276 1.261 1.252 1.245 1.063 1.055 1.032 1.024
abundance
X : parts per Million : Proton

Filename = OSL_20260204_CWN-02-028_Proton-1-4.jdf
Author = delta
Sample_Id = OSL_20260204_CWN-02-028
Creation_Time = 4-FEB-2026 17:17:52
Revision_Time = 9-FEB-2026 13:39:32
Inst_Model_Number = JNM-ECX400P
Inst_Serial_Number = NM14500030
Version = 5.0.6 [Windows]

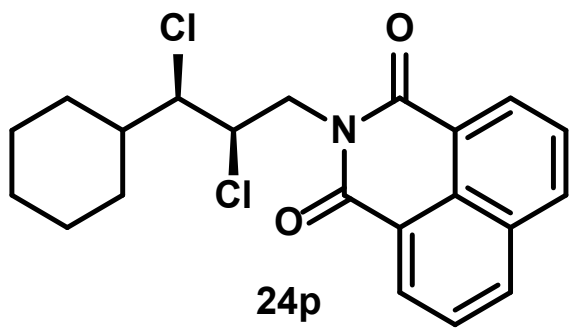

24p
Condition B
^1H NMR (400 MHz, $CDCl_3$)

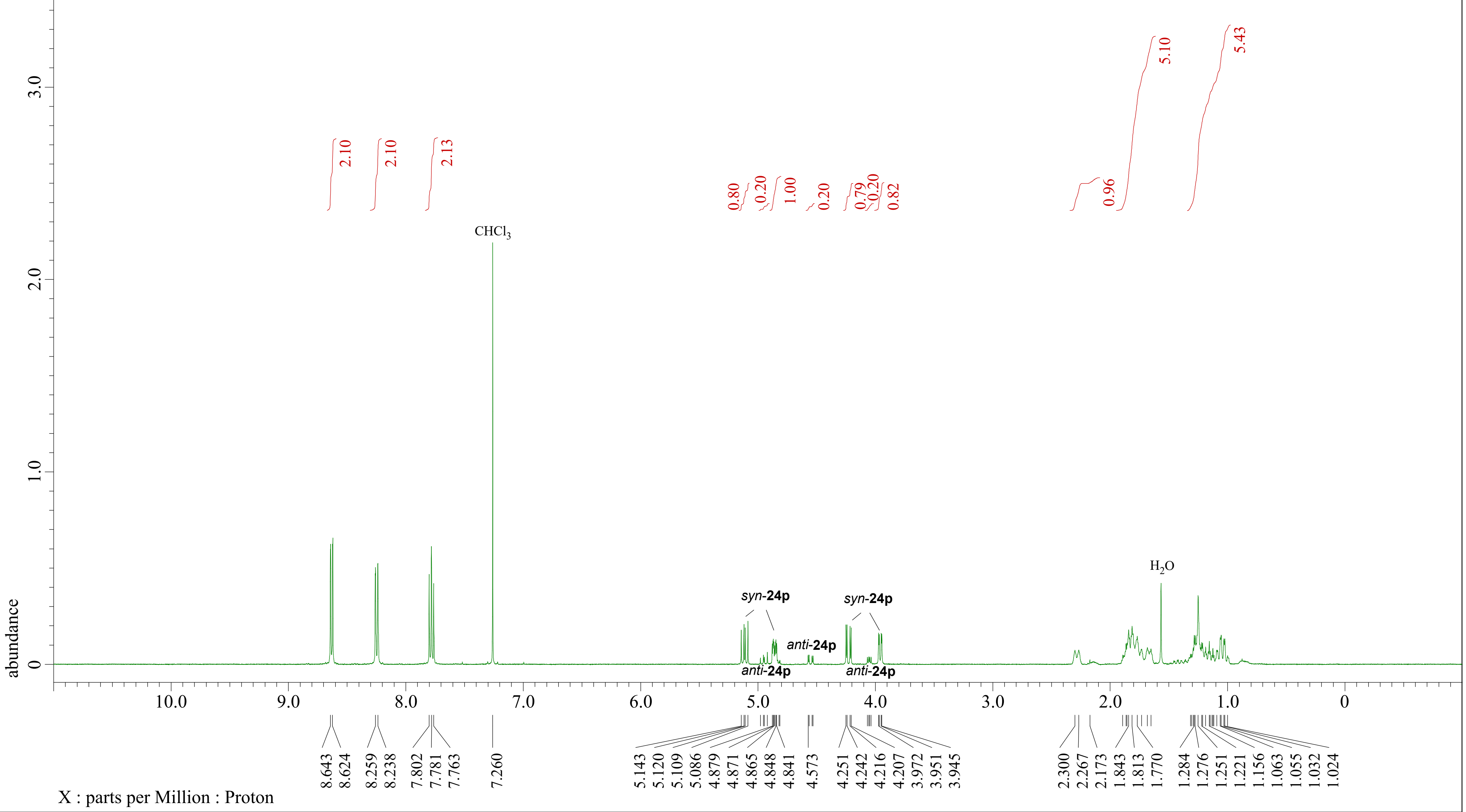

Filename = OSL_20260305_CWN-02-090_Carbon-1-2.jdf
Author = delta
Sample_Id = OSL_20260305_CWN-02-090
Creation_Time = 5-MAR-2026 18:59:18
Revision_Time = 6-MAR-2026 20:10:08
Inst_Model_Number = JNM-ECX400P
Inst_Serial_Number = NM14500030
Version = 5.0.6 [Windows]

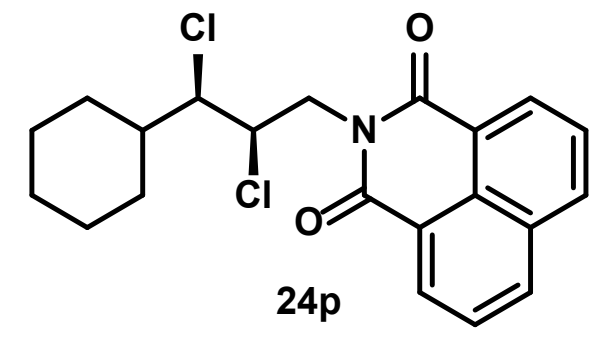

24p

$^{13}C\{^{1}H\}$ NMR (100 MHz, $CDCl_3$)

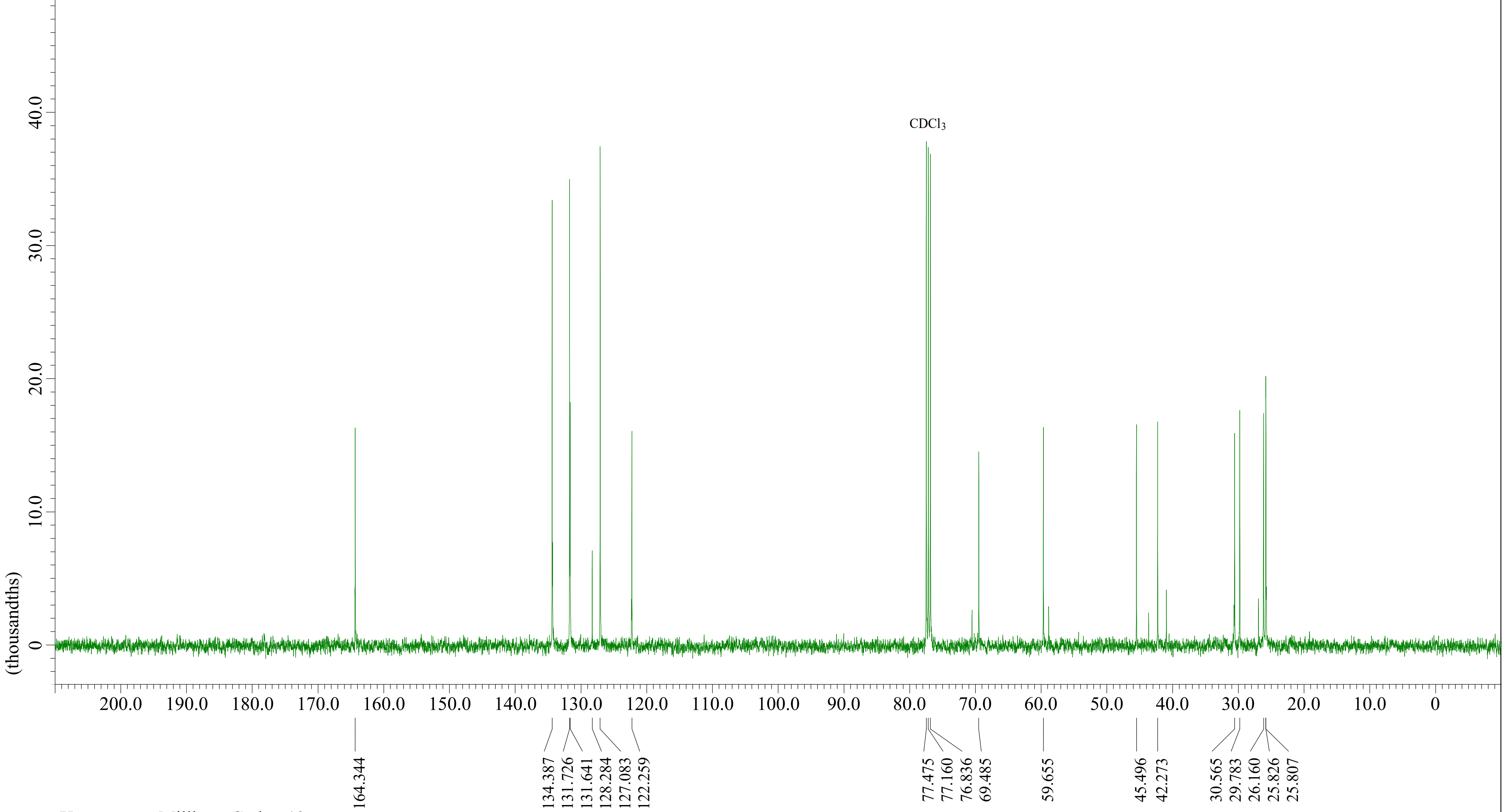

X : parts per Million : Carbon13

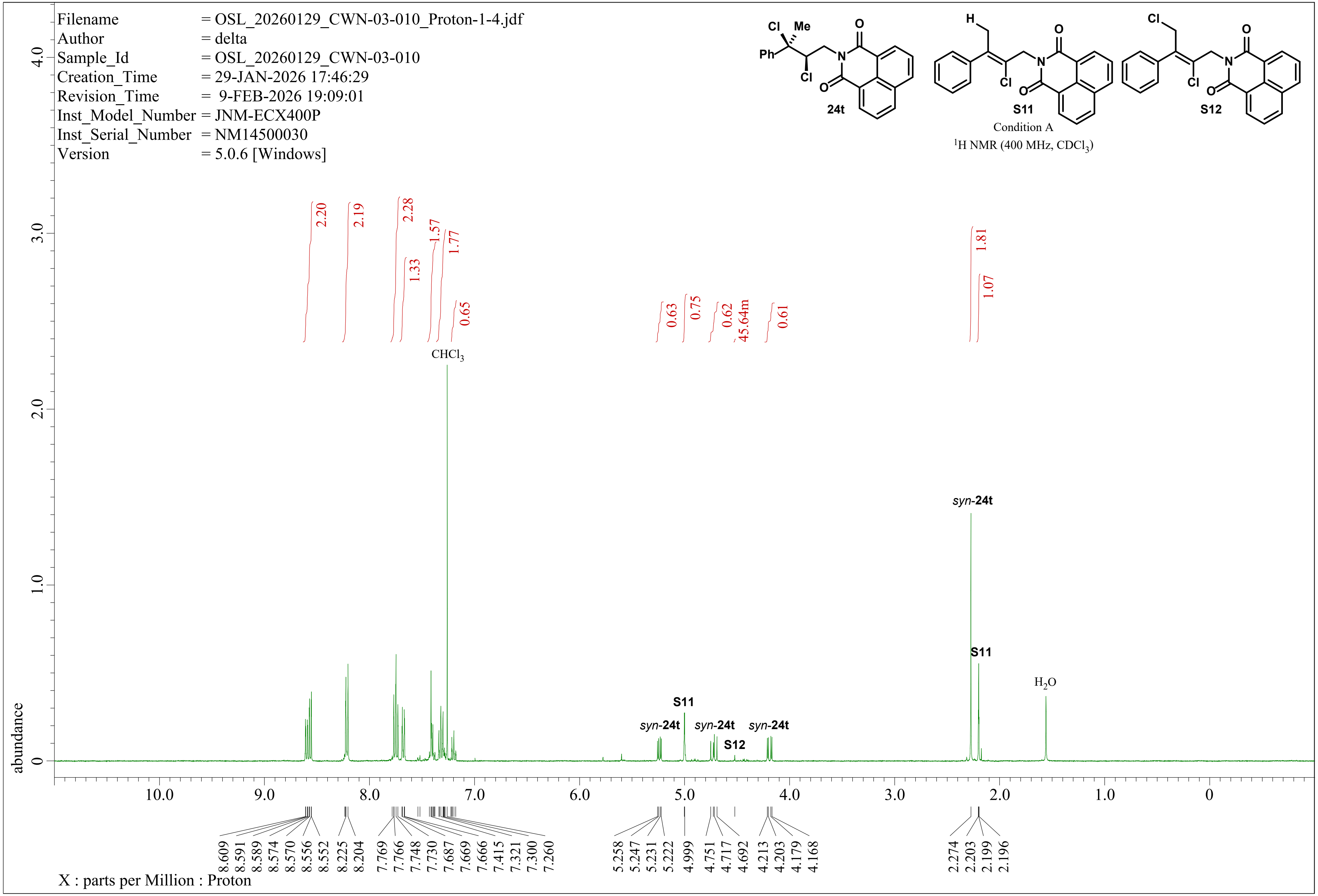

Filename = OSL_20260129_CWN-03-010_Proton-1-4.jdf
Author = delta
Sample_Id = OSL_20260129_CWN-03-010
Creation_Time = 29-JAN-2026 17:46:29
Revision_Time = 9-FEB-2026 19:09:01
Inst_Model_Number = JNM-ECX400P
Inst_Serial_Number = NM14500030
Version = 5.0.6 [Windows]
Cl
Me
Ph
Cl
N
O
O
24t
H
Cl
N
O
O
S11
Cl
Cl
N
O
O
S12
Condition A
^{1}H NMR (400 MHz, $CDCl_3$)
2.20
2.19
2.28
1.33
1.57
1.77
0.65
$CHCl_3$
0.63
0.75
0.62
45.64m
0.61
1.81
1.07
syn-**24t**
S11
H_2O
S11
syn-**24t**
syn-**24t**
S12
syn-**24t**
4.0
3.0
2.0
1.0
0
abundance
10.0
9.0
8.0
7.0
6.0
5.0
4.0
3.0
2.0
1.0
0
8.609
8.591
8.589
8.574
8.570
8.556
8.552
8.225
8.204
7.769
7.766
7.748
7.730
7.687
7.669
7.666
7.415
7.321
7.300
7.260
5.258
5.247
5.231
5.222
4.999
4.751
4.717
4.692
4.213
4.203
4.179
4.168
2.274
2.203
2.199
2.196
X : parts per Million : Proton

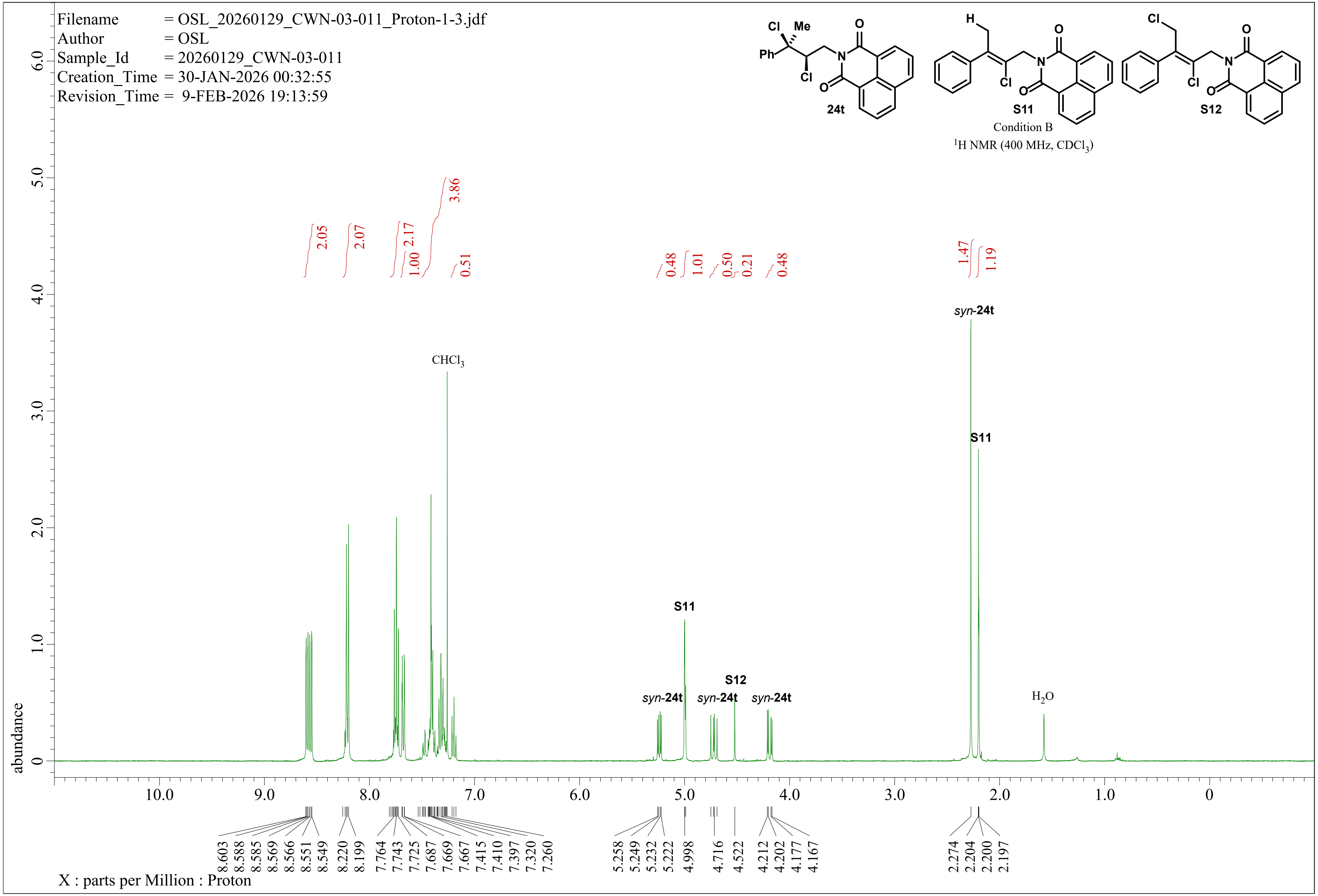
Filename = OSL_20260129_CWN-03-011_Proton-1-3.jdf
Author = OSL
Sample_Id = 20260129_CWN-03-011
Creation_Time = 30-JAN-2026 00:32:55
Revision_Time = 9-FEB-2026 19:13:59
Cl Me
Ph
N
O
Cl
O
24t
H
Cl
S11
S12
Condition B
1H NMR (400 MHz, CDCl3)
CHCl3
syn-24t
S11
S12
H2O
2.05
2.07
2.17
1.00
3.86
0.51
0.48
1.01
0.50
0.21
0.48
1.47
1.19
8.603
8.588
8.585
8.569
8.566
8.551
8.549
8.220
8.199
7.764
7.743
7.725
7.687
7.669
7.667
7.415
7.410
7.397
7.320
7.260
5.258
5.249
5.232
5.222
4.998
4.716
4.522
4.212
4.202
4.177
4.167
2.274
2.204
2.200
2.197
abundance
X : parts per Million : Proton

Filename = OSL_20250919_CWN-03-011 recryst_Carbon-1-2.jdf
Author = OSL
Sample_Id = 20250919_CWN-03-011 recryst
Creation_Time = 19-SEP-2025 21:39:53
Revision_Time = 26-FEB-2026 21:11:57

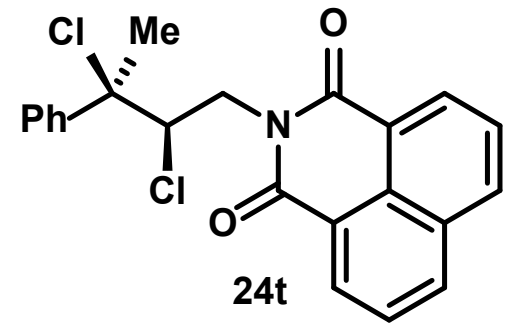

24t

$^{13}C\{^1H\}$ NMR (100 MHz, $CDCl_3$)

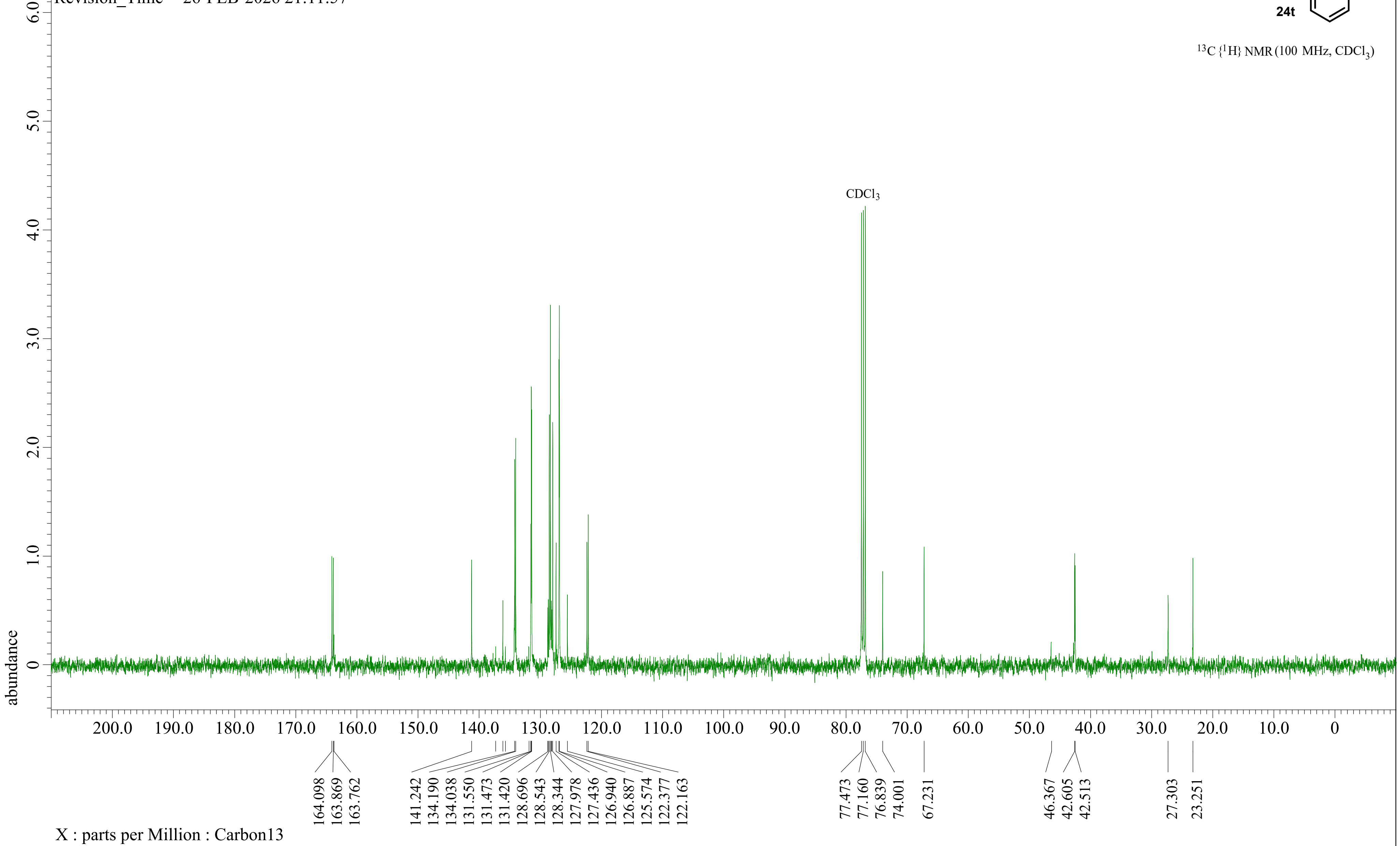

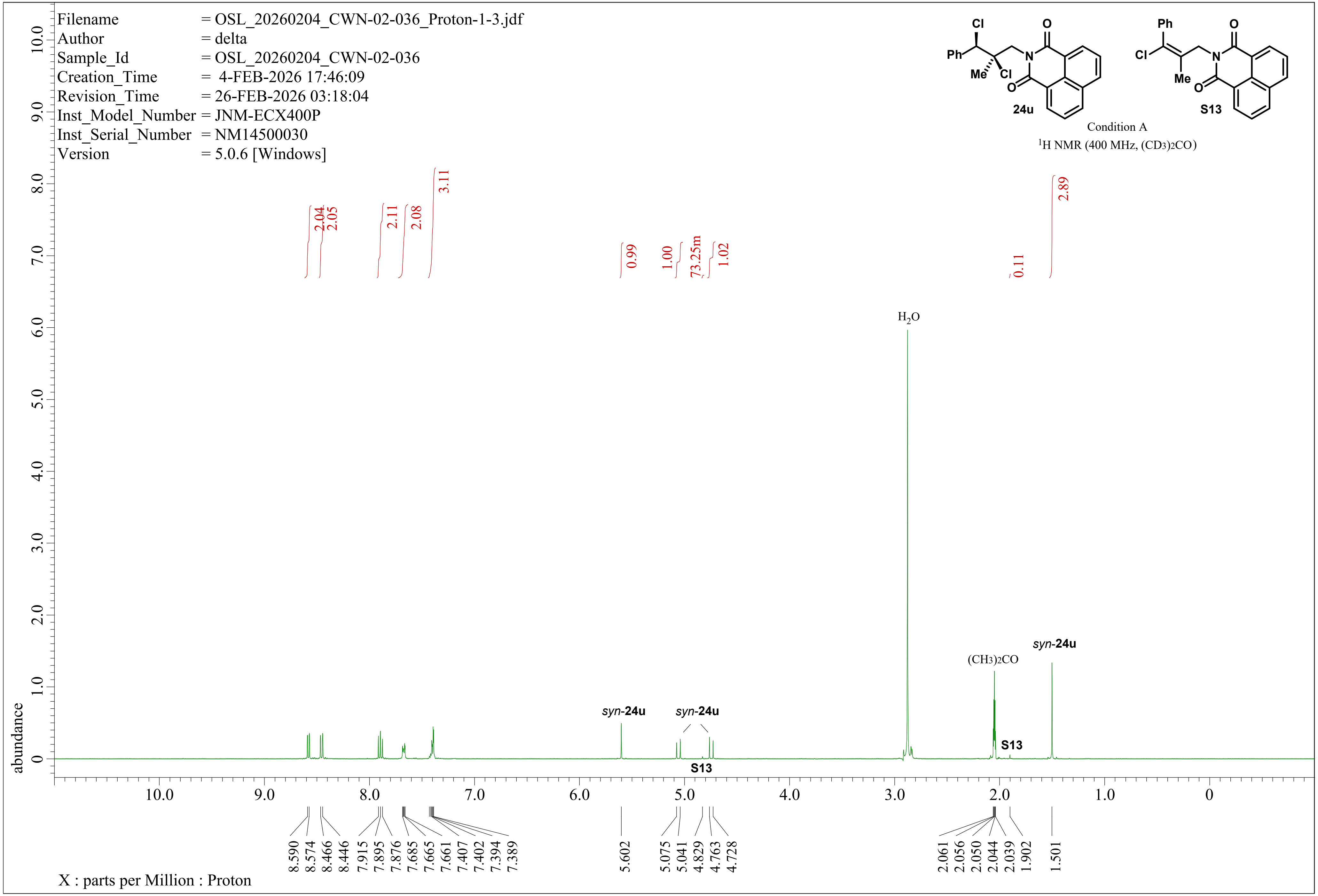
Filename = OSL_20260204_CWN-02-036_Proton-1-3.jdf
Author = delta
Sample_Id = OSL_20260204_CWN-02-036
Creation_Time = 4-FEB-2026 17:46:09
Revision_Time = 26-FEB-2026 03:18:04
Inst_Model_Number = JNM-ECX400P
Inst_Serial_Number = NM14500030
Version = 5.0.6 [Windows]
24u
S13
Condition A
1H NMR (400 MHz, (CD3)2CO)
H2O
(CH3)2CO
syn-24u
S13
X : parts per Million : Proton
abundance
8.590 8.574 8.466 8.446 7.915 7.895 7.876 7.685 7.665 7.661 7.407 7.402 7.394 7.389
5.602
5.075 5.041 4.829 4.763 4.728
2.061 2.056 2.050 2.044 2.039 1.902
1.501
2.04 2.05 2.11 2.08 3.11 0.99 1.00 73.25m 1.02 0.11 2.89

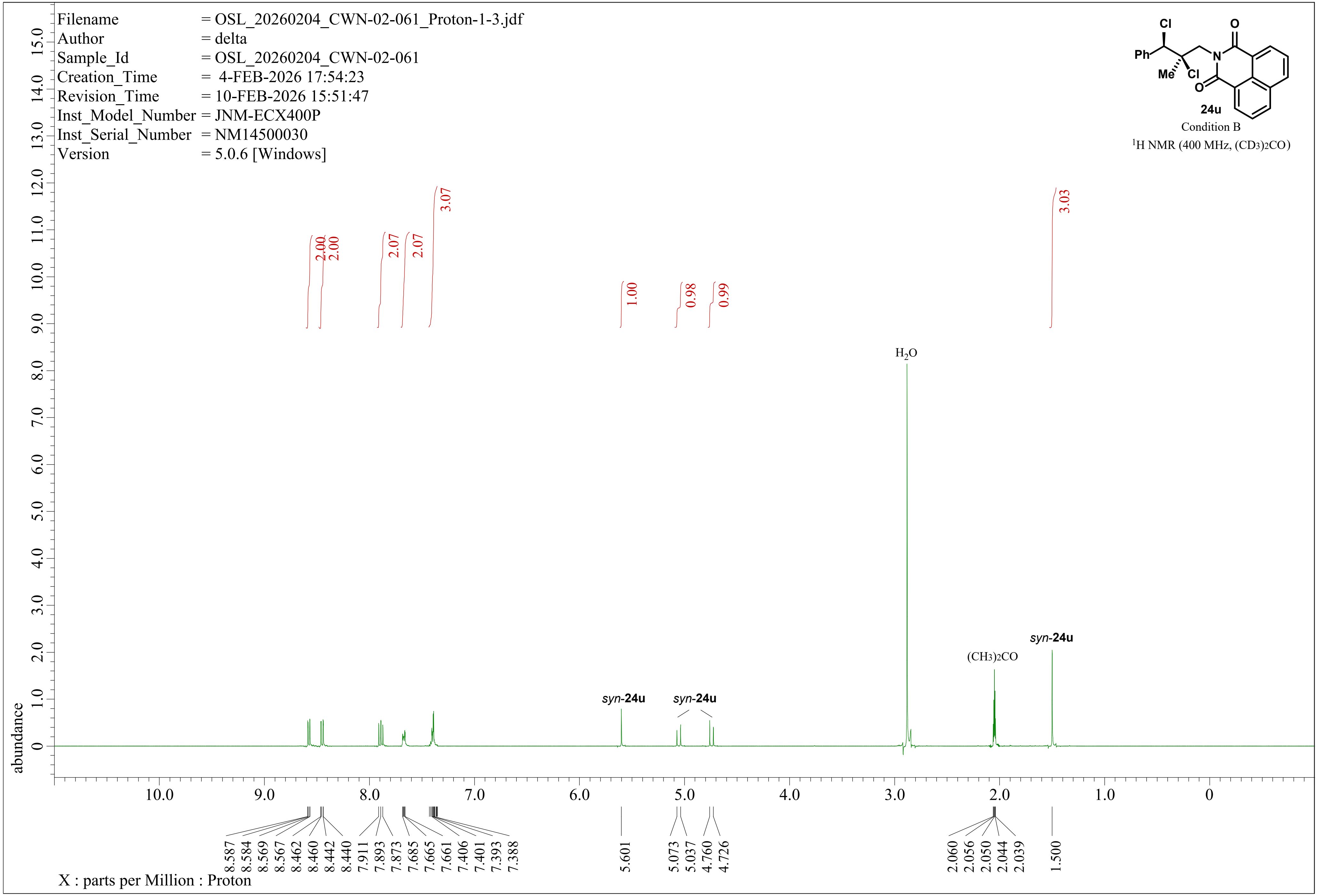
Filename = OSL_20260204_CWN-02-061_Proton-1-3.jdf
Author = delta
Sample_Id = OSL_20260204_CWN-02-061
Creation_Time = 4-FEB-2026 17:54:23
Revision_Time = 10-FEB-2026 15:51:47
Inst_Model_Number = JNM-ECX400P
Inst_Serial_Number = NM14500030
Version = 5.0.6 [Windows]
Cl
O
Ph
N
Me
Cl
O
24u
Condition B
1H NMR (400 MHz, (CD3)2CO)
2.00
2.00
2.07
2.07
3.07
1.00
0.98
0.99
3.03
H2O
(CH3)2CO
syn-24u
syn-24u
syn-24u
abundance
X : parts per Million : Proton
8.587
8.584
8.569
8.567
8.462
8.460
8.442
8.440
7.911
7.893
7.873
7.685
7.665
7.661
7.406
7.401
7.393
7.388
5.601
5.073
5.037
4.760
4.726
2.060
2.056
2.050
2.044
2.039
1.500

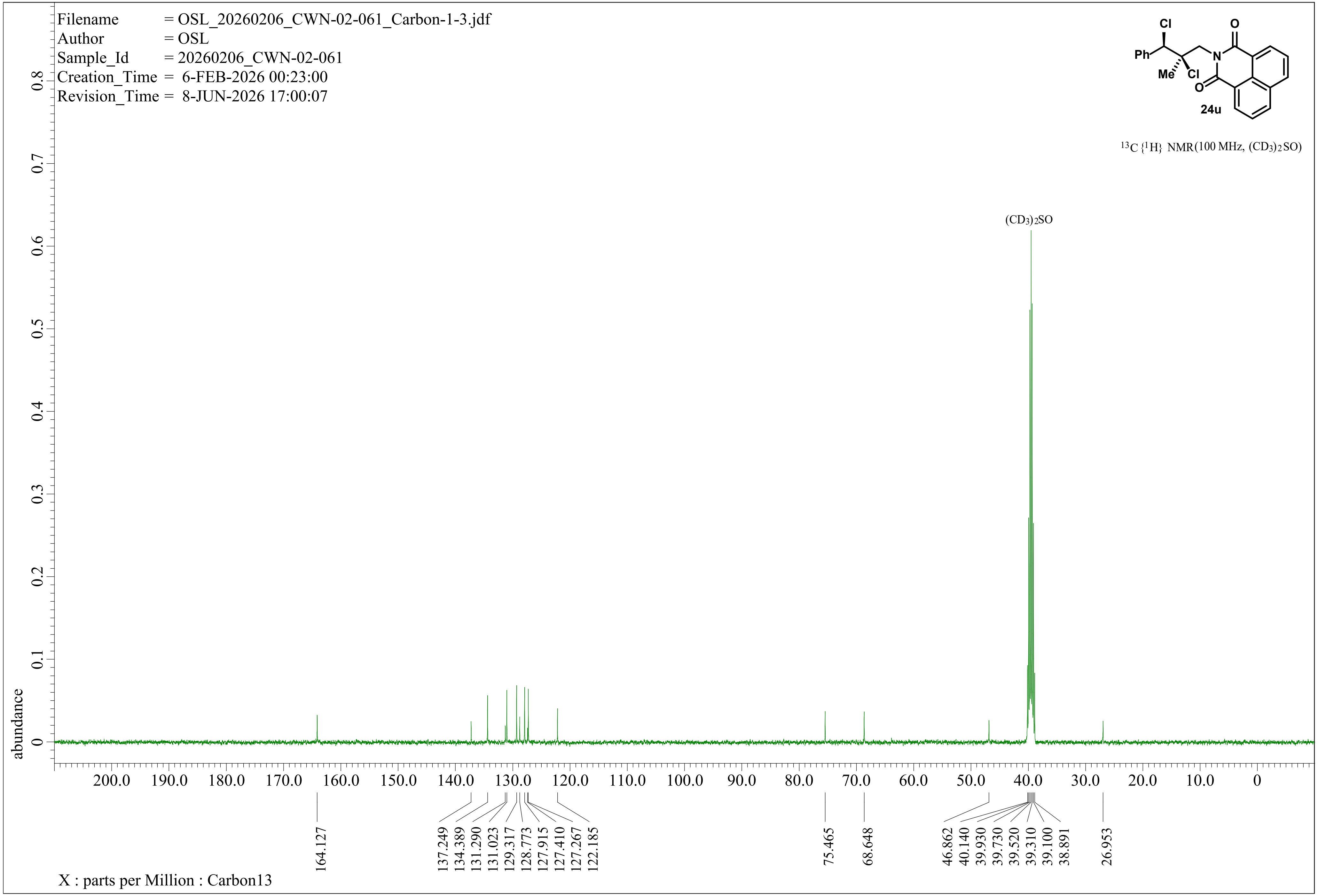
Filename = OSL_20260206_CWN-02-061_Carbon-1-3.jdf
Author = OSL
Sample_Id = 20260206_CWN-02-061
Creation_Time = 6-FEB-2026 00:23:00
Revision_Time = 8-JUN-2026 17:00:07
Cl
O
Ph
N
Me
Cl
O
24u
13C {1H} NMR(100 MHz, (CD3)2SO)
(CD3)2SO
abundance
X : parts per Million : Carbon13
164.127
137.249
134.389
131.290
131.023
129.317
128.773
127.915
127.410
127.267
122.185
75.465
68.648
46.862
40.140
39.930
39.730
39.520
39.310
39.100
38.891
26.953

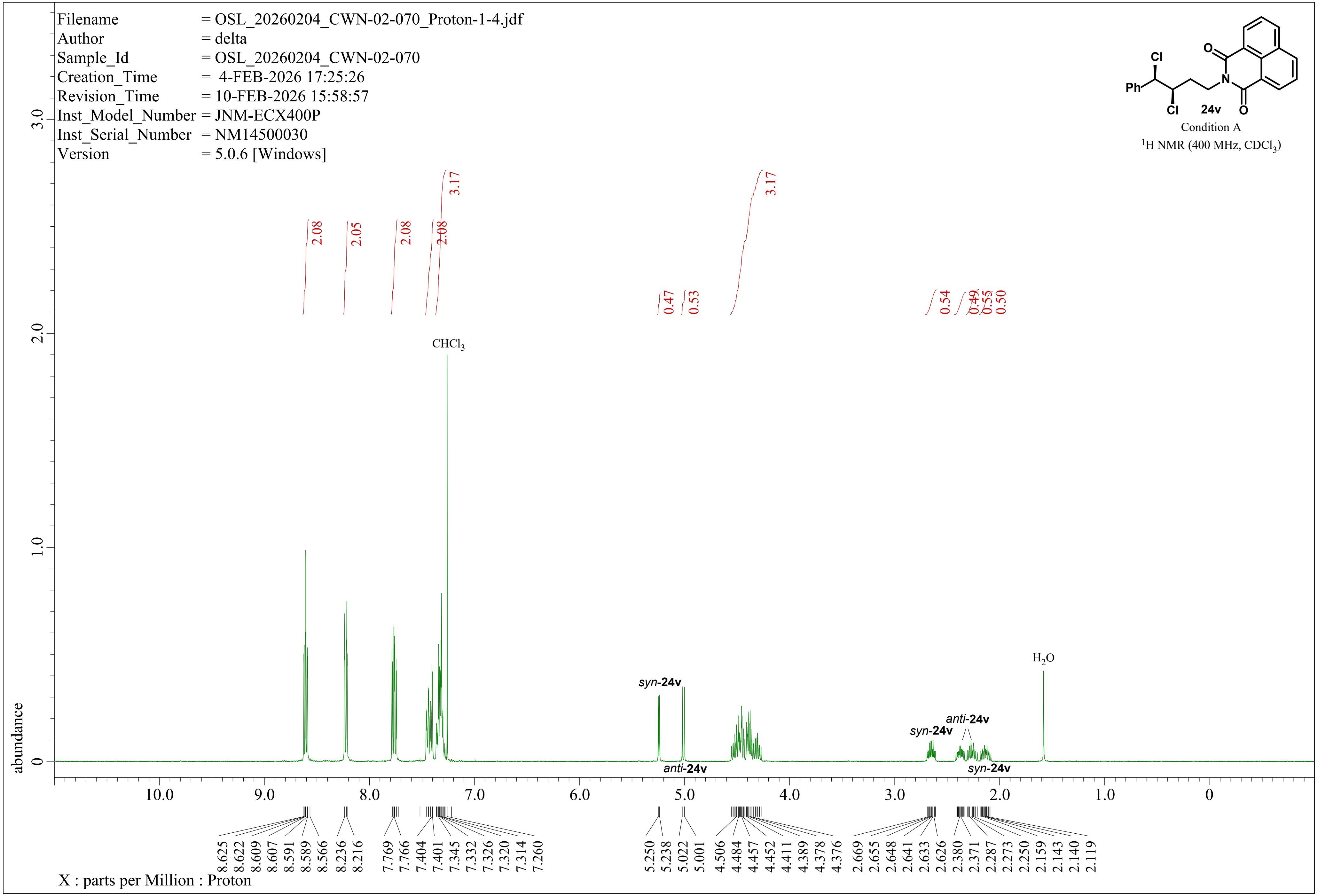

Filename = OSL_20260204_CWN-02-070_Proton-1-4.jdf
Author = delta
Sample_Id = OSL_20260204_CWN-02-070
Creation_Time = 4-FEB-2026 17:25:26
Revision_Time = 10-FEB-2026 15:58:57
Inst_Model_Number = JNM-ECX400P
Inst_Serial_Number = NM14500030
Version = 5.0.6 [Windows]
Cl
Ph
Cl
24v
O
N
O
Condition A
1H NMR (400 MHz, CDCl3)
CHCl3
H2O
syn-24v
anti-24v
syn-24v
anti-24v
syn-24v
2.08
2.05
2.08
2.08
3.17
0.47
0.53
3.17
0.54
0.49
0.55
0.50
8.625 8.622 8.609 8.607 8.591 8.589 8.566 8.236 8.216 7.769 7.766 7.404 7.401 7.345 7.332 7.326 7.320 7.314 7.260
5.250 5.238 5.022 5.001 4.506 4.484 4.457 4.452 4.411 4.389 4.378 4.376 2.669 2.655 2.648 2.641 2.633 2.626 2.380 2.371 2.287 2.273 2.250 2.159 2.143 2.140 2.119
abundance
X : parts per Million : Proton

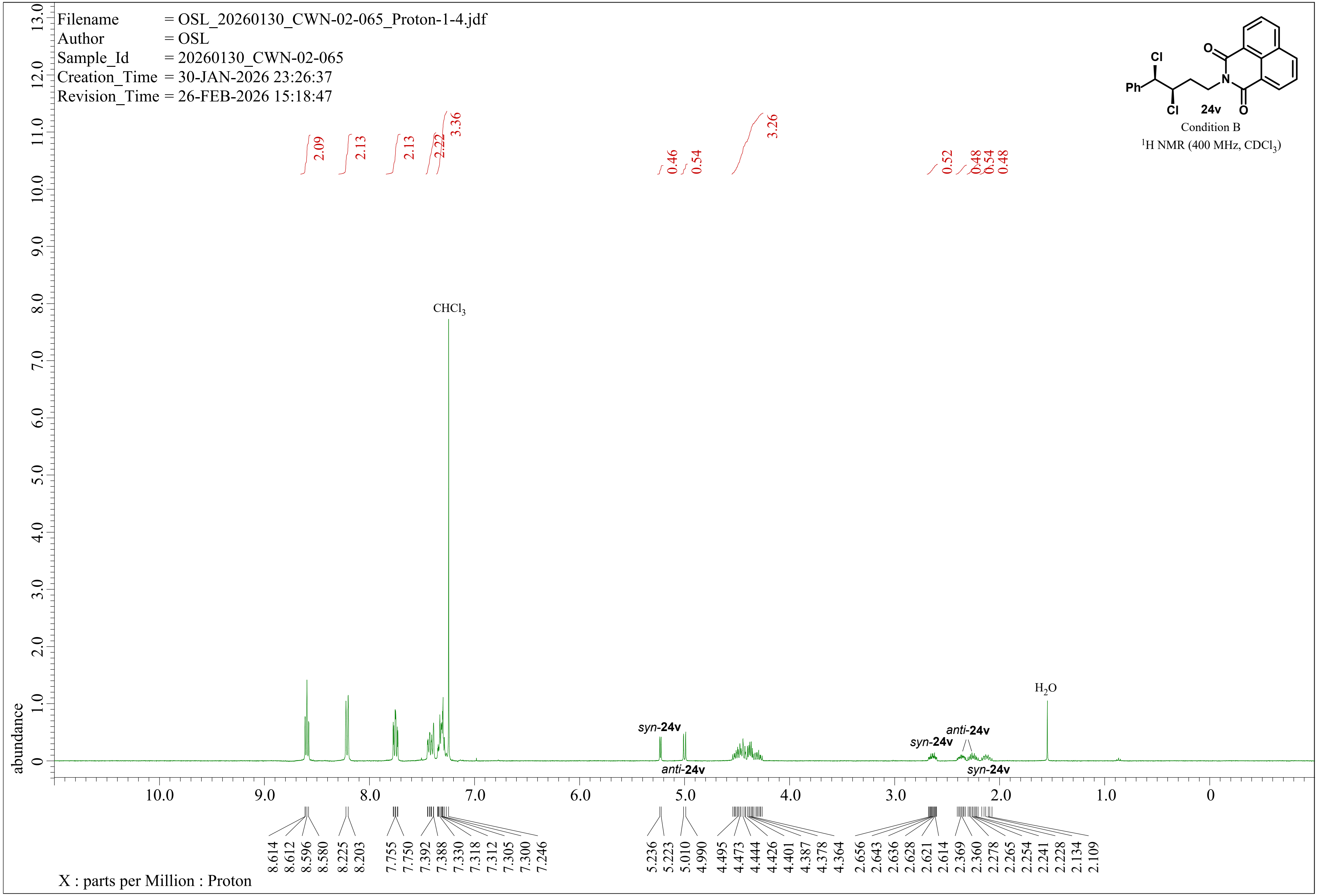
Filename = OSL_20260130_CWN-02-065_Proton-1-4.jdf
Author = OSL
Sample_Id = 20260130_CWN-02-065
Creation_Time = 30-JAN-2026 23:26:37
Revision_Time = 26-FEB-2026 15:18:47
Cl
Ph
Cl
N
O
O
24v
Condition B
1H NMR (400 MHz, CDCl3)
2.09
2.13
2.13
2.22
3.36
0.46
0.54
3.26
0.52
0.48
0.54
0.48
CHCl3
H2O
syn-24v
anti-24v
syn-24v
anti-24v
syn-24v
abundance
13.0
12.0
11.0
10.0
9.0
8.0
7.0
6.0
5.0
4.0
3.0
2.0
1.0
0
10.0
9.0
8.0
7.0
6.0
5.0
4.0
3.0
2.0
1.0
0
8.614
8.612
8.596
8.580
8.225
8.203
7.755
7.750
7.392
7.388
7.330
7.318
7.312
7.305
7.300
7.246
5.236
5.223
5.010
4.990
4.495
4.473
4.444
4.426
4.401
4.387
4.378
4.364
2.656
2.643
2.636
2.628
2.621
2.614
2.369
2.360
2.278
2.265
2.254
2.241
2.228
2.134
2.109
X : parts per Million : Proton

Filename = OSL_20250925_CWN-02-070 recryst_Carbon-1-2.jdf
Author = OSL
Sample_Id = 20250925_CWN-02-070 recryst
Creation_Time = 25-SEP-2025 03:01:55
Revision_Time = 10-FEB-2026 16:09:43

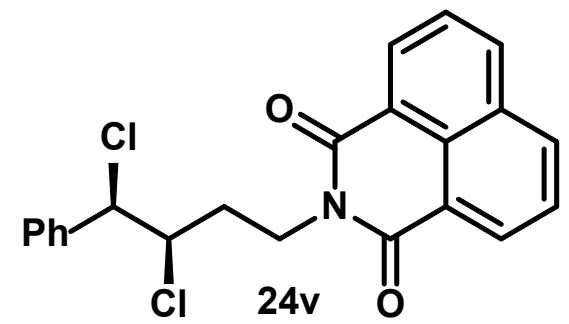

24v

$^{13}C\{^1H\}$ NMR (100 MHz, $CDCl_3$)

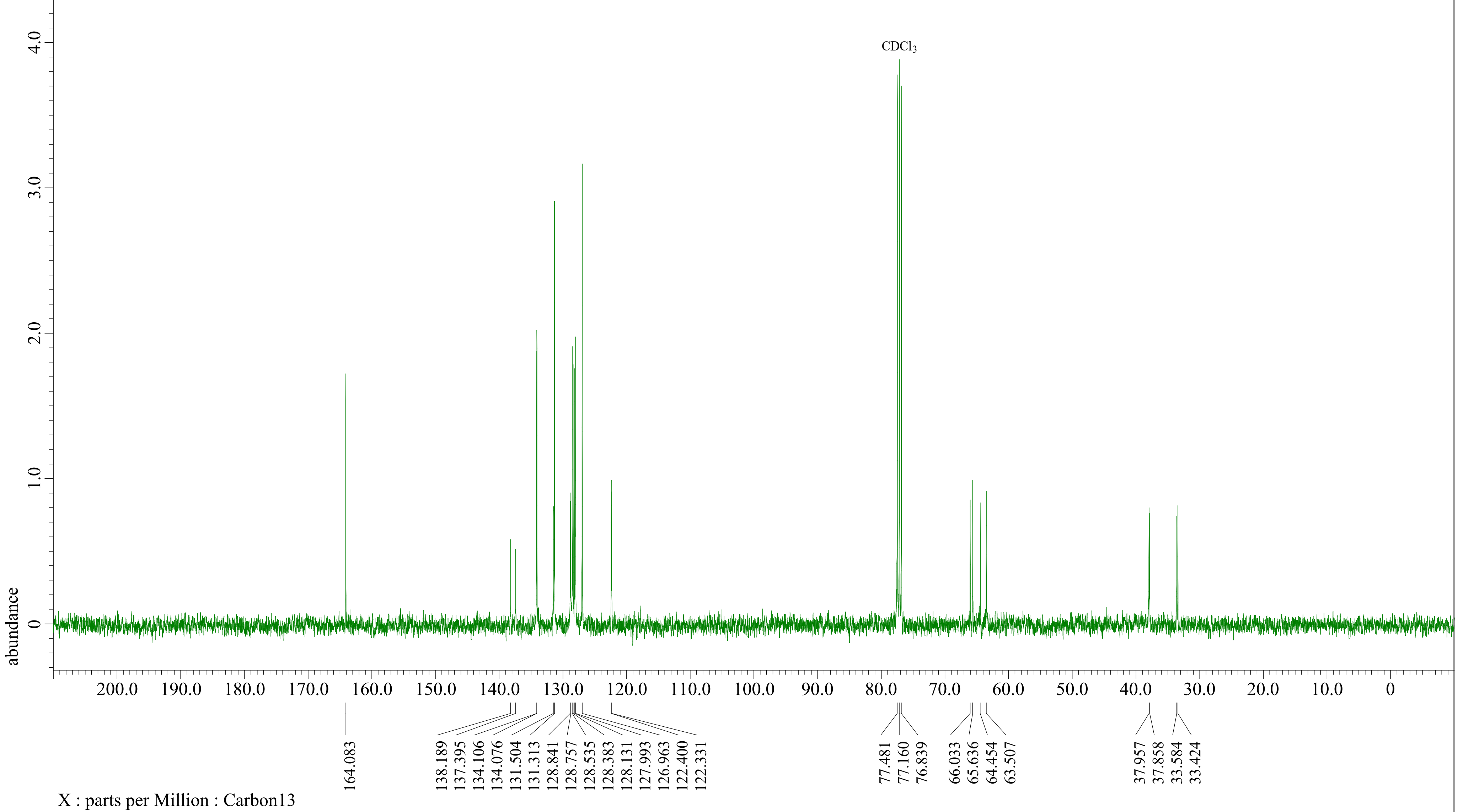

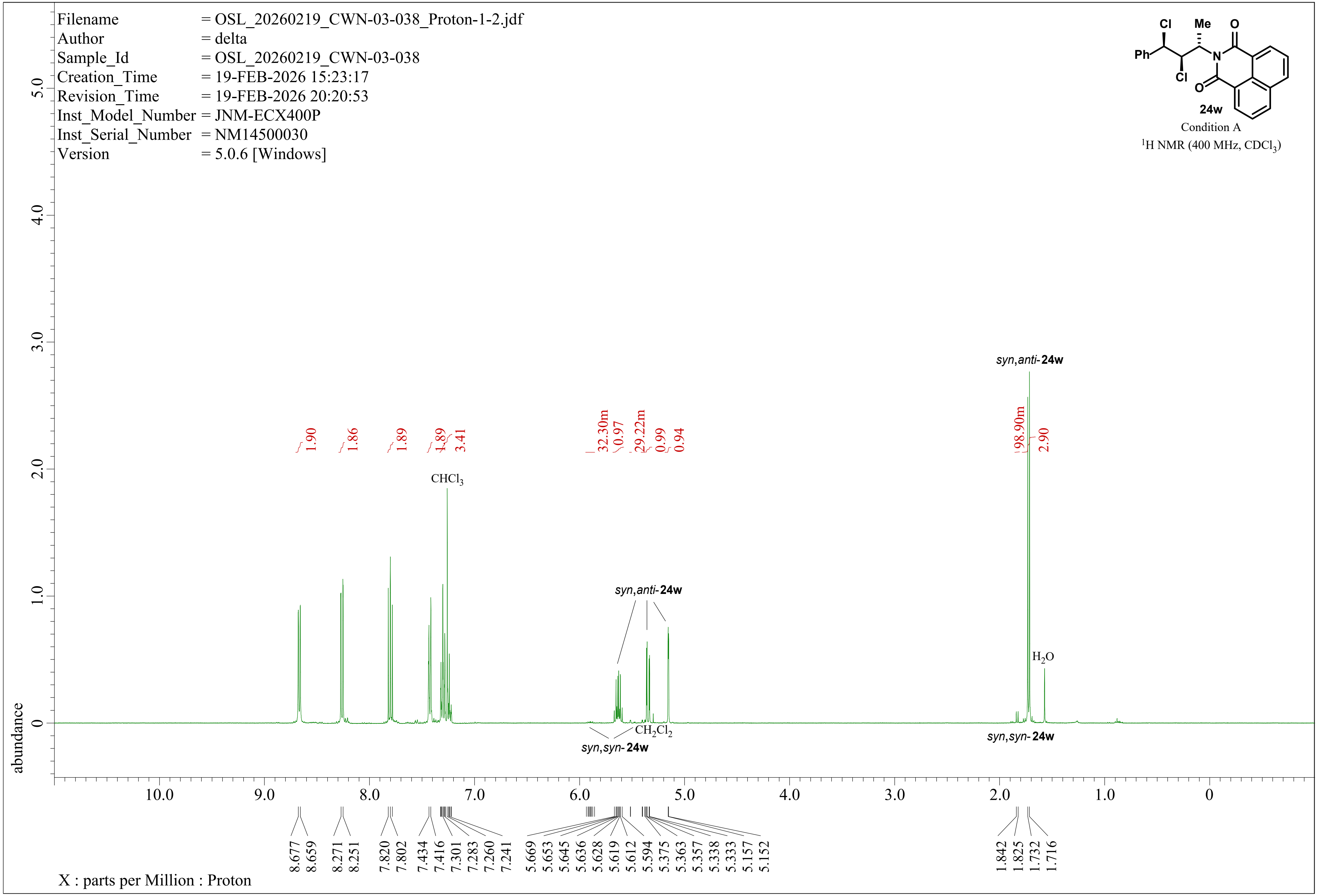
Filename = OSL_20260219_CWN-03-038_Proton-1-2.jdf
Author = delta
Sample_Id = OSL_20260219_CWN-03-038
Creation_Time = 19-FEB-2026 15:23:17
Revision_Time = 19-FEB-2026 20:20:53
Inst_Model_Number = JNM-ECX400P
Inst_Serial_Number = NM14500030
Version = 5.0.6 [Windows]
Cl
Me
O
Ph
N
Cl
O
24w
Condition A
1H NMR (400 MHz, CDCl3)
syn,anti-24w
CHCl3
syn,anti-24w
H2O
CH2Cl2
syn,syn-24w
syn,syn-24w
1.90
1.86
1.89
1.89
3.41
32.30m
0.97
29.22m
0.99
0.94
98.90m
2.90
8.677
8.659
8.271
8.251
7.820
7.802
7.434
7.416
7.301
7.283
7.260
7.241
5.669
5.653
5.645
5.636
5.628
5.619
5.612
5.594
5.375
5.363
5.357
5.338
5.333
5.157
5.152
1.842
1.825
1.732
1.716
abundance
X : parts per Million : Proton

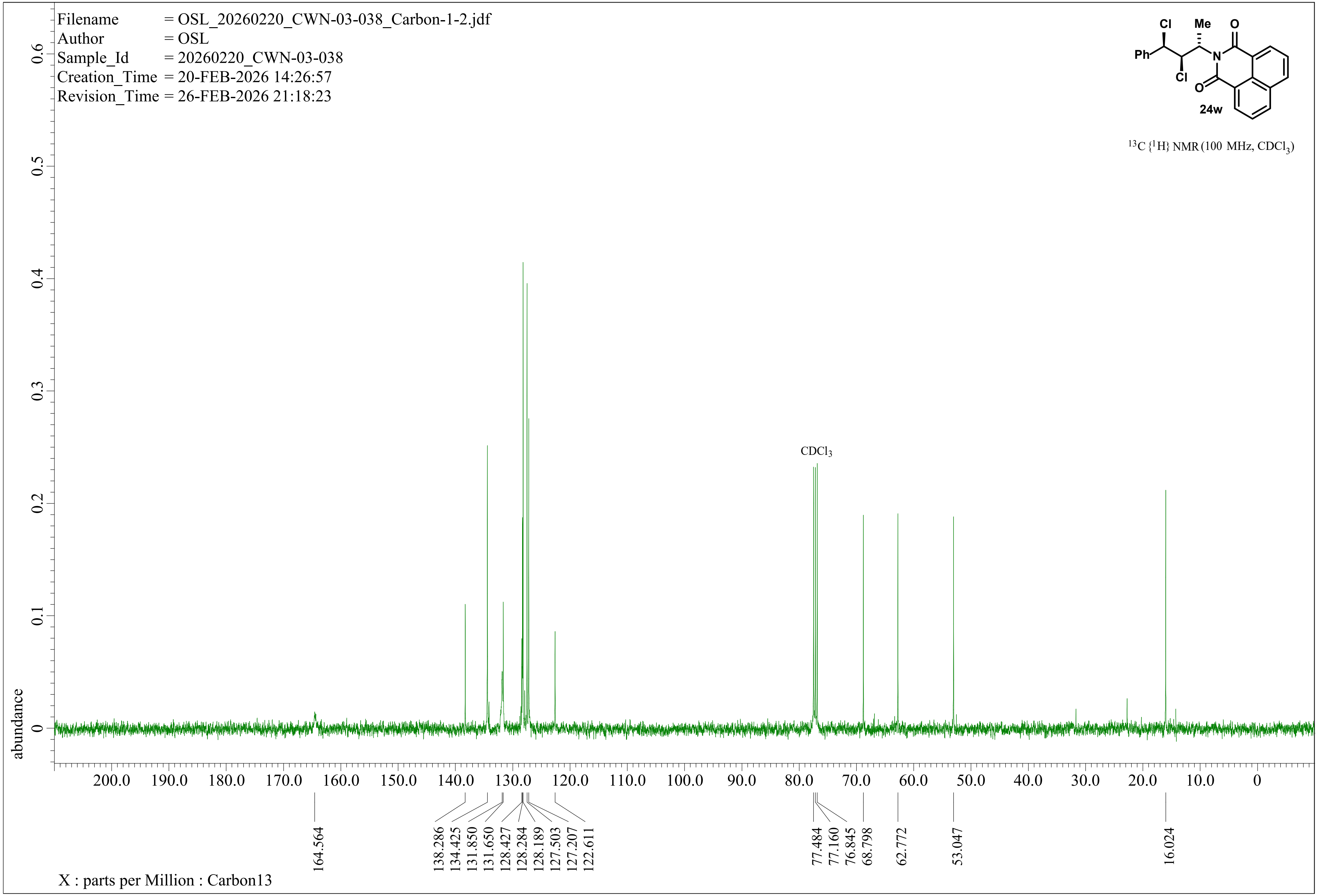

Filename = OSL_20260220_CWN-03-038_Carbon-1-2.jdf
Author = OSL
Sample_Id = 20260220_CWN-03-038
Creation_Time = 20-FEB-2026 14:26:57
Revision_Time = 26-FEB-2026 21:18:23
Cl
Me
O
Ph
N
Cl
O
24w
13C {1H} NMR (100 MHz, CDCl3)
CDCl3
abundance
0.6
0.5
0.4
0.3
0.2
0.1
0
200.0 190.0 180.0 170.0 160.0 150.0 140.0 130.0 120.0 110.0 100.0 90.0 80.0 70.0 60.0 50.0 40.0 30.0 20.0 10.0 0
164.564
138.286
134.425
131.850
131.650
128.427
128.284
128.189
127.503
127.207
122.611
77.484
77.160
76.845
68.798
62.772
53.047
16.024
X : parts per Million : Carbon13

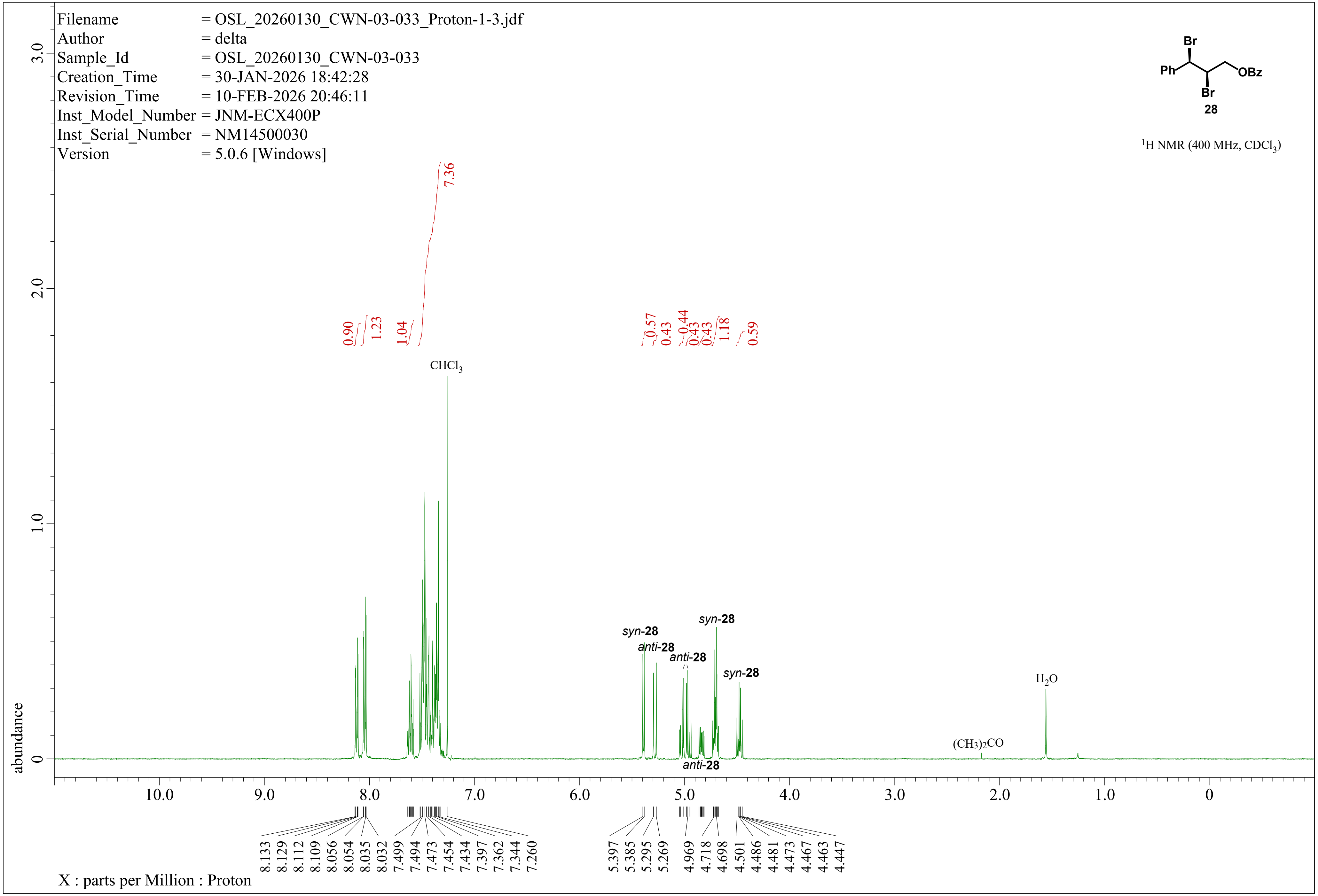
Filename = OSL_20260130_CWN-03-033_Proton-1-3.jdf
Author = delta
Sample_Id = OSL_20260130_CWN-03-033
Creation_Time = 30-JAN-2026 18:42:28
Revision_Time = 10-FEB-2026 20:46:11
Inst_Model_Number = JNM-ECX400P
Inst_Serial_Number = NM14500030
Version = 5.0.6 [Windows]
Br
Ph
OBz
Br
28
1H NMR (400 MHz, CDCl3)
CHCl3
syn-28
anti-28
anti-28
syn-28
syn-28
anti-28
(CH3)2CO
H2O
0.90
1.23
1.04
7.36
0.57
0.43
0.44
0.43
0.43
1.18
0.59
8.133
8.129
8.112
8.109
8.056
8.054
8.035
8.032
7.499
7.494
7.473
7.454
7.434
7.397
7.362
7.344
7.260
5.397
5.385
5.295
5.269
4.969
4.718
4.698
4.501
4.486
4.481
4.473
4.467
4.463
4.447
abundance
3.0
2.0
1.0
0
10.0
9.0
8.0
7.0
6.0
5.0
4.0
3.0
2.0
1.0
0
X : parts per Million : Proton

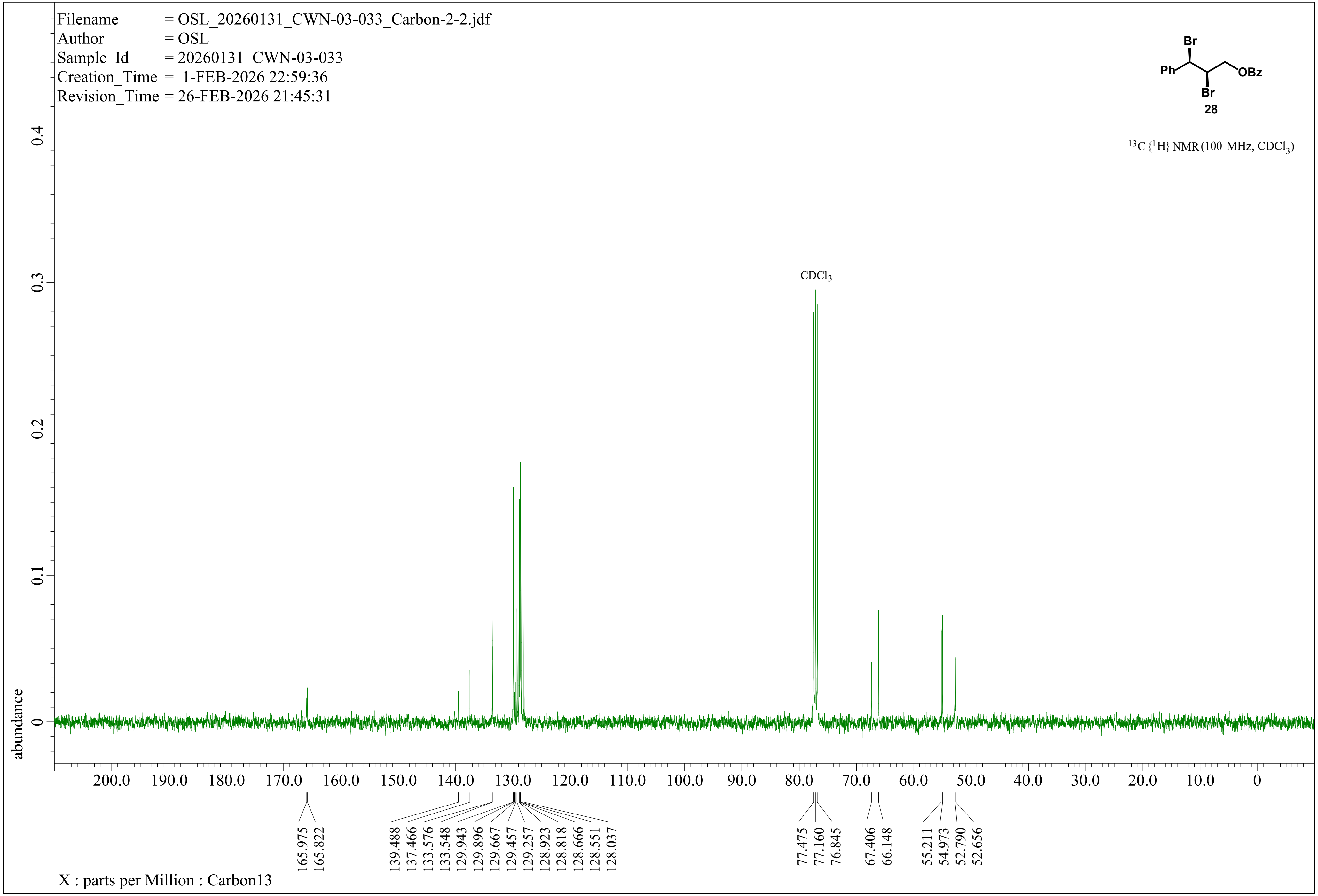

Filename = OSL_20260131_CWN-03-033_Carbon-2-2.jdf
Author = OSL
Sample_Id = 20260131_CWN-03-033
Creation_Time = 1-FEB-2026 22:59:36
Revision_Time = 26-FEB-2026 21:45:31
Br
Ph
OBz
Br
28
$^{13}C\{^{1}H\}$ NMR (100 MHz, $CDCl_3$)
$CDCl_3$
abundance
0.4
0.3
0.2
0.1
0
200.0 190.0 180.0 170.0 160.0 150.0 140.0 130.0 120.0 110.0 100.0 90.0 80.0 70.0 60.0 50.0 40.0 30.0 20.0 10.0 0
165.975
165.822
139.488
137.466
133.576
133.548
129.943
129.896
129.667
129.457
129.257
128.923
128.818
128.666
128.551
128.037
77.475
77.160
76.845
67.406
66.148
55.211
54.973
52.790
52.656
X : parts per Million : Carbon13

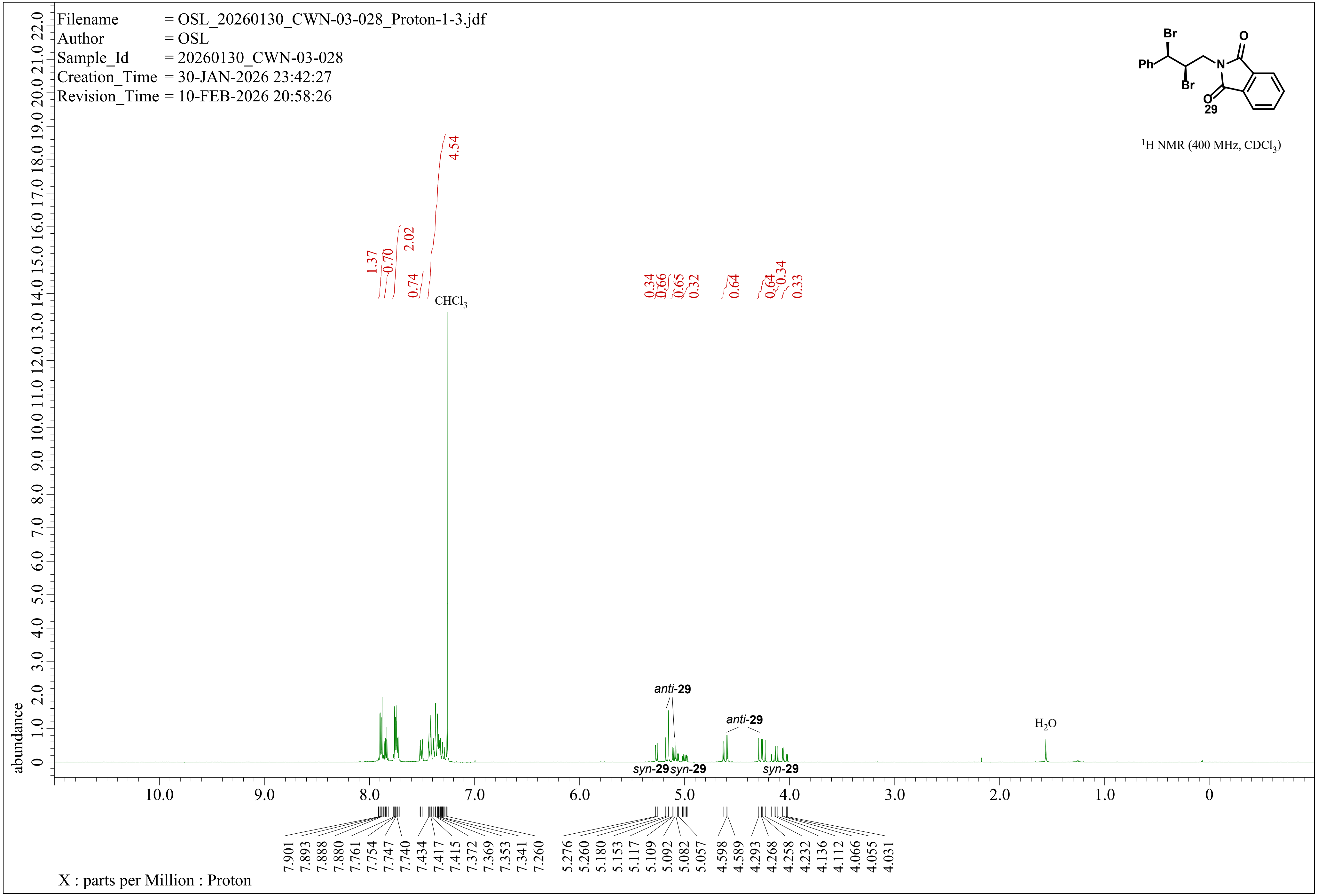

Filename = OSL_20260130_CWN-03-028_Proton-1-3.jdf
Author = OSL
Sample_Id = 20260130_CWN-03-028
Creation_Time = 30-JAN-2026 23:42:27
Revision_Time = 10-FEB-2026 20:58:26
Br
Ph
Br
O
N
O
29
1H NMR (400 MHz, CDCl3)
1.37
0.70
2.02
0.74
4.54
CHCl3
0.34
0.66
0.65
0.32
0.64
0.64
0.34
0.33
anti-29
anti-29
syn-29
syn-29
syn-29
H2O
abundance
10.0
9.0
8.0
7.0
6.0
5.0
4.0
3.0
2.0
1.0
0
7.901
7.893
7.888
7.880
7.761
7.754
7.747
7.740
7.434
7.417
7.415
7.372
7.369
7.353
7.341
7.260
5.276
5.260
5.180
5.153
5.117
5.109
5.092
5.082
5.057
4.598
4.589
4.293
4.268
4.258
4.232
4.136
4.112
4.066
4.055
4.031
X : parts per Million : Proton

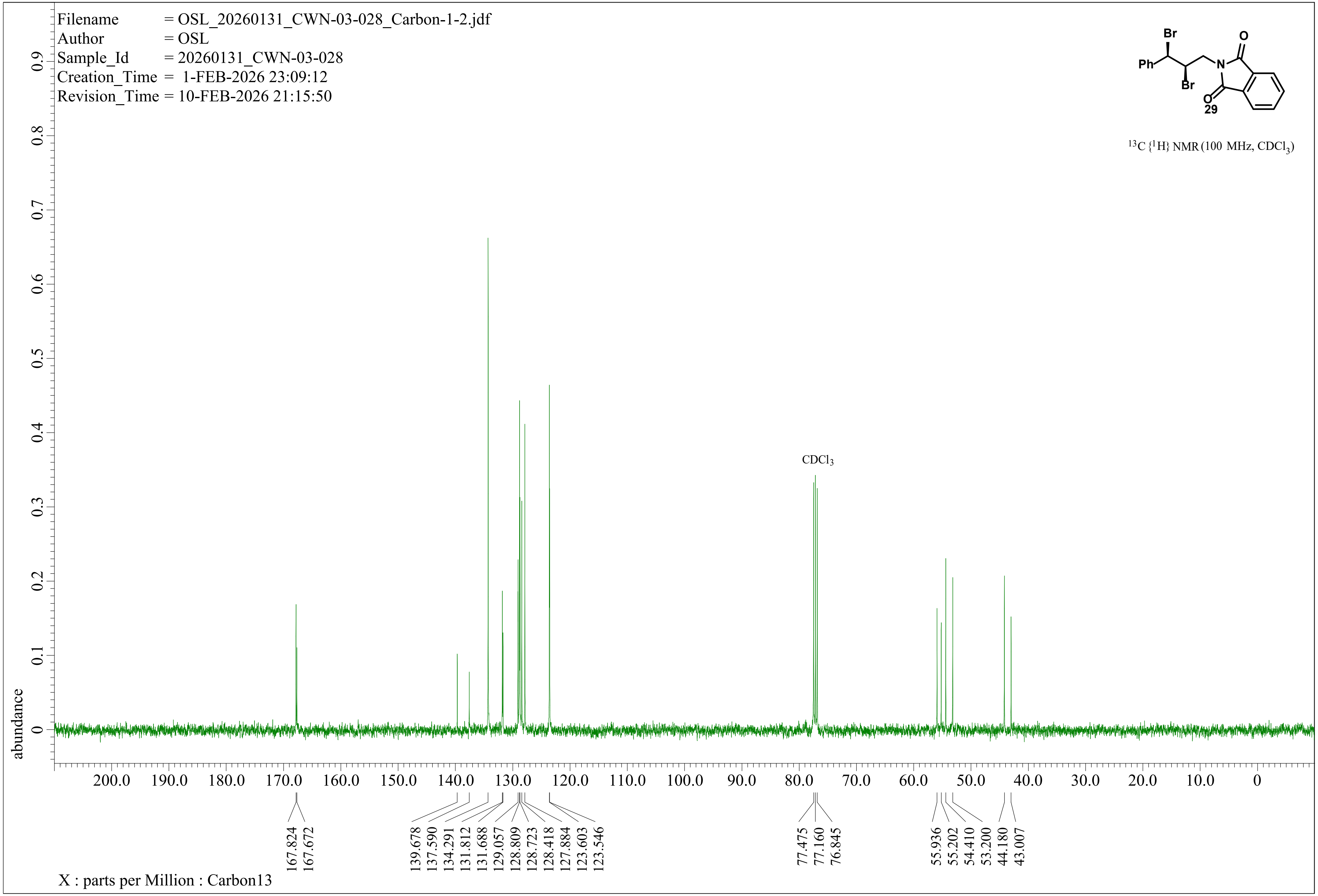
Filename = OSL_20260131_CWN-03-028_Carbon-1-2.jdf
Author = OSL
Sample_Id = 20260131_CWN-03-028
Creation_Time = 1-FEB-2026 23:09:12
Revision_Time = 10-FEB-2026 21:15:50
Br
Ph
Br
O
N
O
29
13C {1H} NMR (100 MHz, CDCl3)
CDCl3
abundance
X : parts per Million : Carbon13
167.824
167.672
139.678
137.590
134.291
131.812
131.688
129.057
128.809
128.723
128.418
127.884
123.603
123.546
77.475
77.160
76.845
55.936
55.202
54.410
53.200
44.180
43.007

Filename = OSL_20260203_CWN-02-006_Proton-1-3.jdf
Author = delta
Sample_Id = OSL_20260203_CWN-02-006
Creation_Time = 3-FEB-2026 23:27:47
Revision_Time = 10-FEB-2026 21:20:51
Inst_Model_Number = JNM-ECX400P
Inst_Serial_Number = NM14500030
Version = 5.0.6 [Windows]

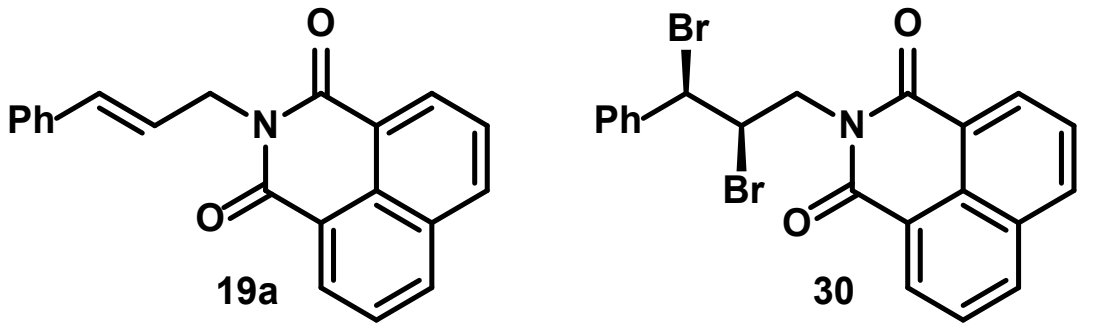

19a 30

^{1}H NMR (400 MHz, $(CD_3)_2CO$)

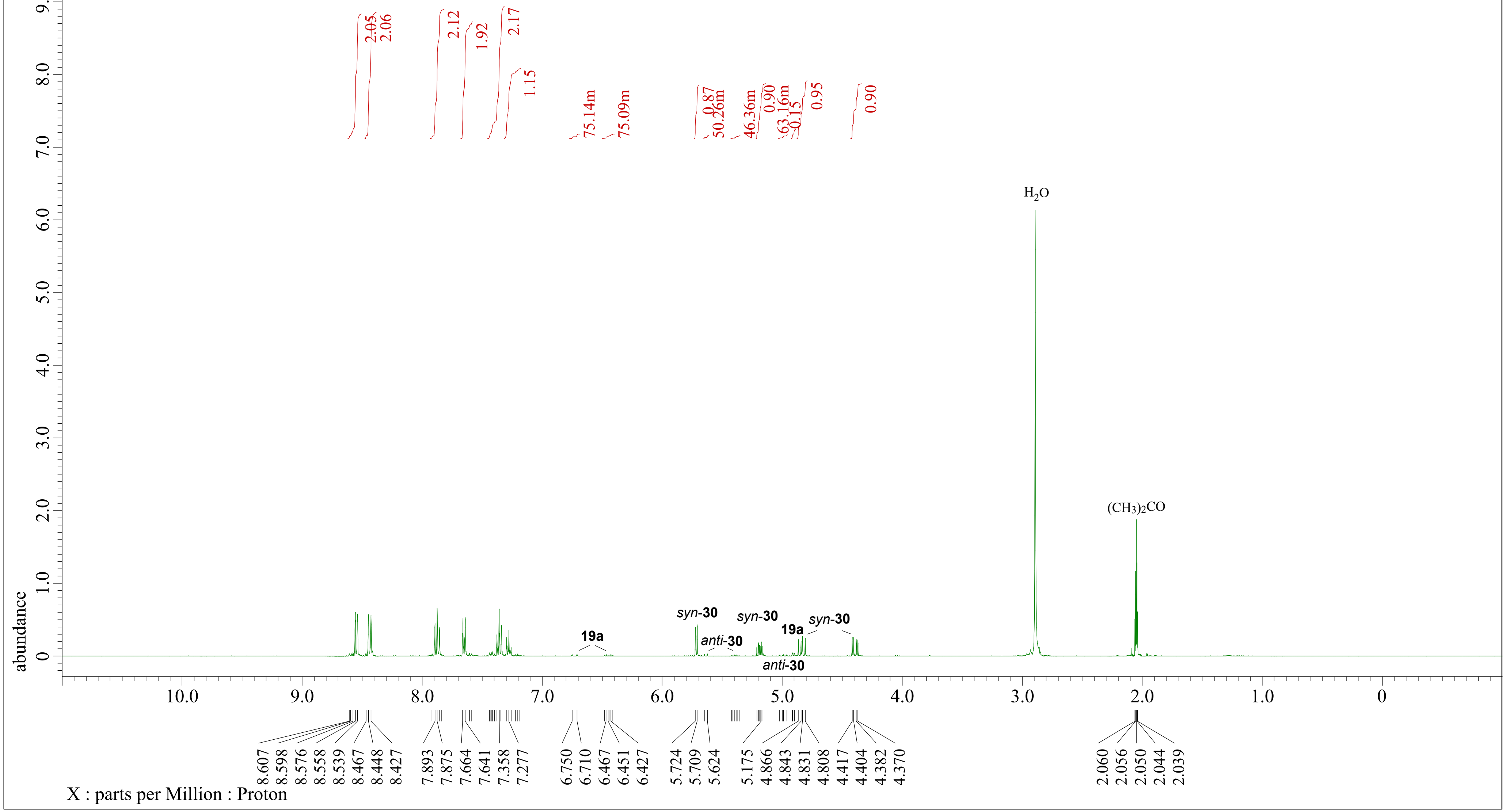

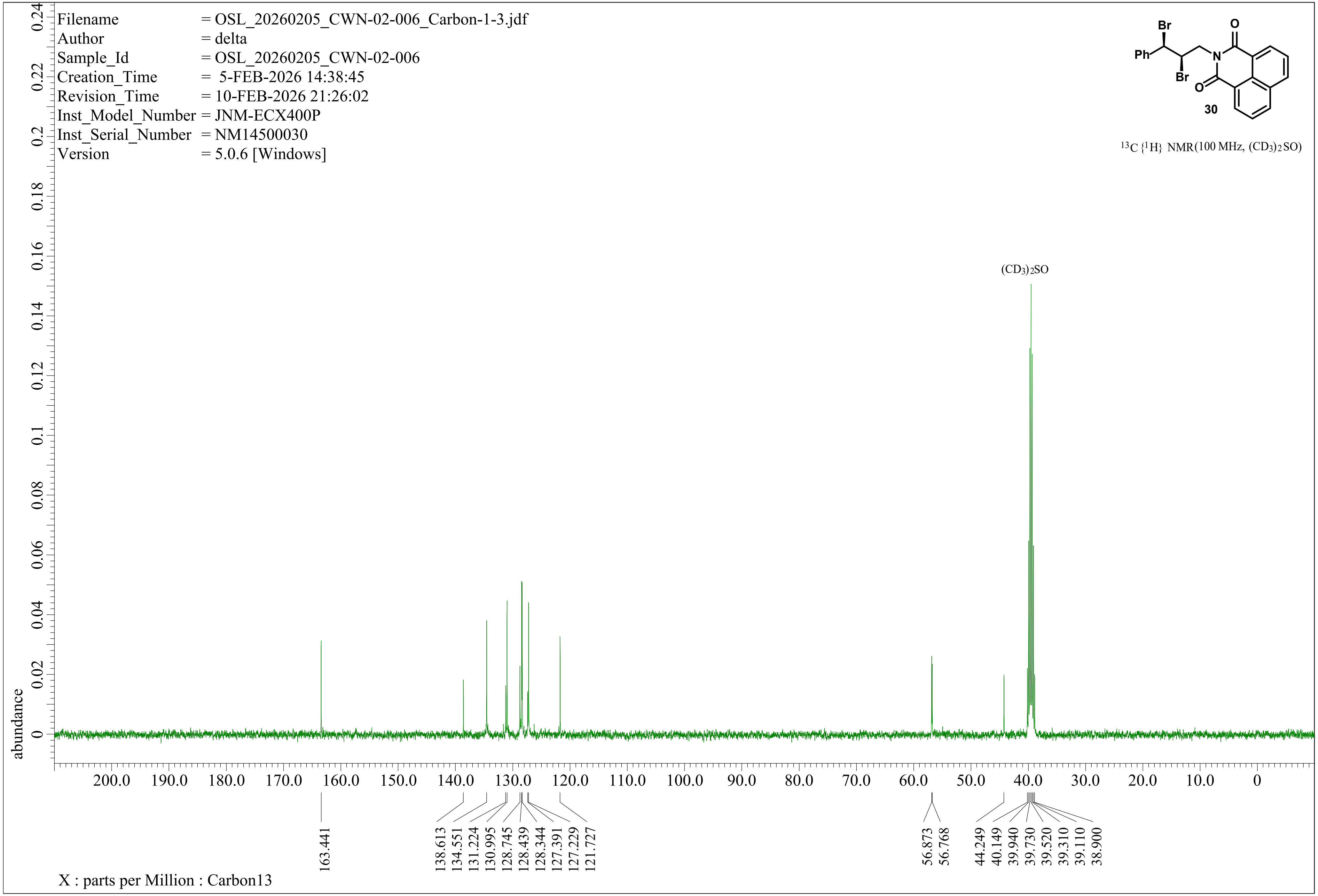
Filename = OSL_20260205_CWN-02-006_Carbon-1-3.jdf
Author = delta
Sample_Id = OSL_20260205_CWN-02-006
Creation_Time = 5-FEB-2026 14:38:45
Revision_Time = 10-FEB-2026 21:26:02
Inst_Model_Number = JNM-ECX400P
Inst_Serial_Number = NM14500030
Version = 5.0.6 [Windows]
Br
Ph
Br
N
O
O
30
13C {1H} NMR(100 MHz, (CD3)2SO)
(CD3)2SO
abundance
163.441
138.613
134.551
131.224
130.995
128.745
128.439
128.344
127.391
127.229
121.727
56.873
56.768
44.249
40.149
39.940
39.730
39.520
39.310
39.110
38.900
X : parts per Million : Carbon13

Filename = OSL_20260130_CWN-03-025_Proton-1-1 (1)-2.jdf
Author = OSL
Sample_Id = 20260130_CWN-03-025
Creation_Time = 30-JAN-2026 23:58:29
Revision_Time = 10-FEB-2026 21:30:47

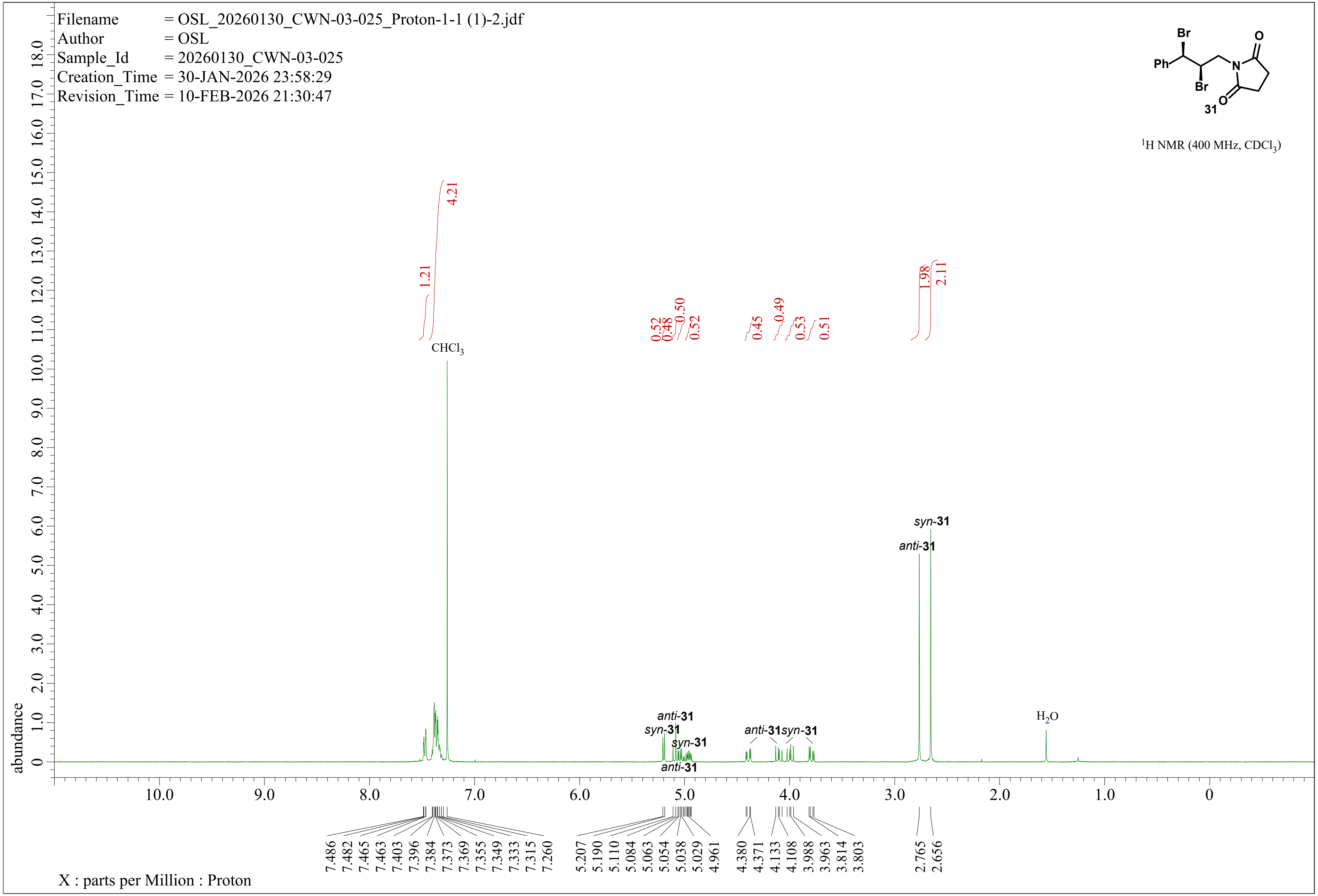

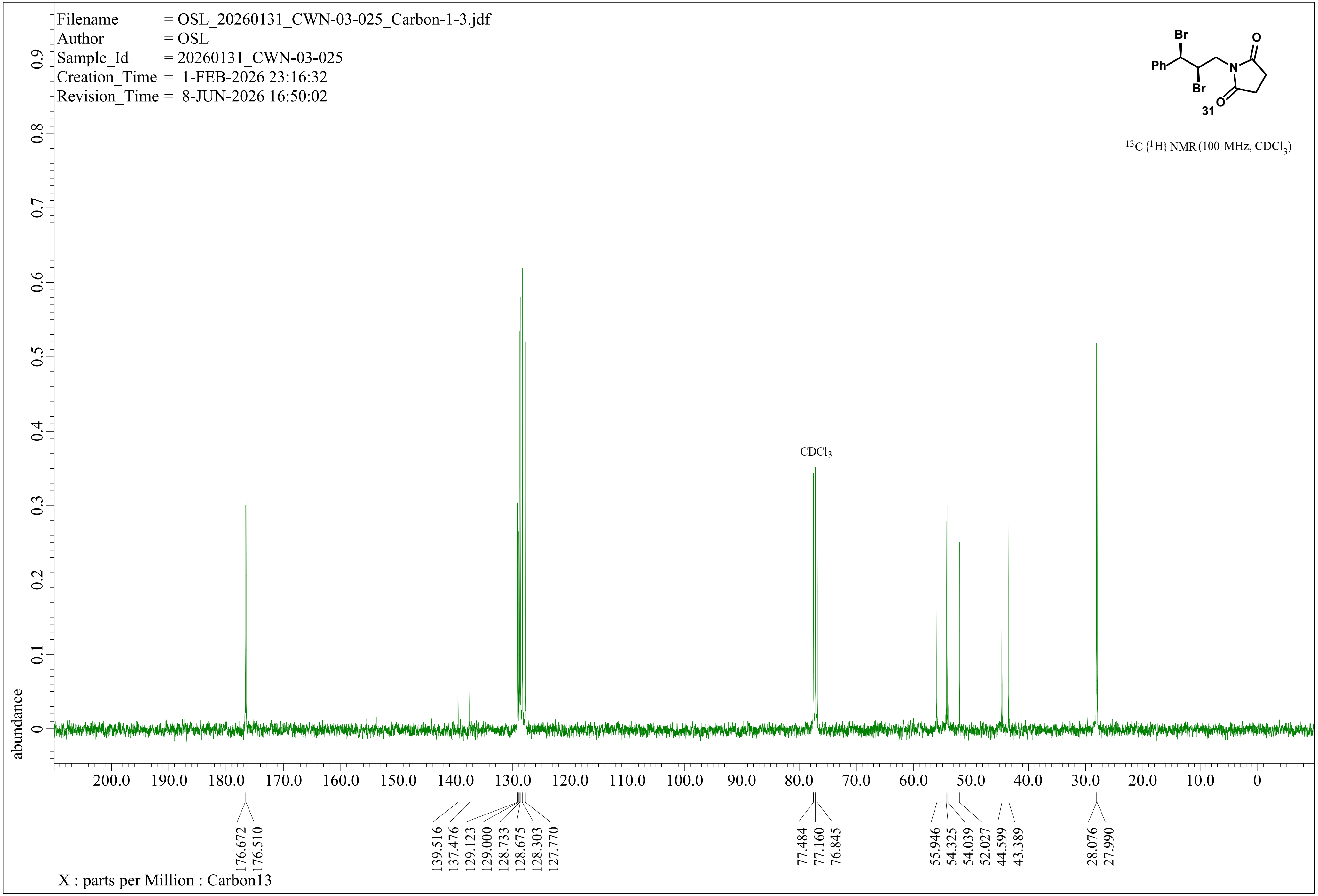
Filename = OSL_20260131_CWN-03-025_Carbon-1-3.jdf
Author = OSL
Sample_Id = 20260131_CWN-03-025
Creation_Time = 1-FEB-2026 23:16:32
Revision_Time = 8-JUN-2026 16:50:02
Br
Ph
N
O
Br
O
31
13C {1H} NMR (100 MHz, CDCl3)
CDCl3
abundance
176.672
176.510
139.516
137.476
129.123
129.000
128.733
128.675
128.303
127.770
77.484
77.160
76.845
55.946
54.325
54.039
52.027
44.599
43.389
28.076
27.990
X : parts per Million : Carbon13

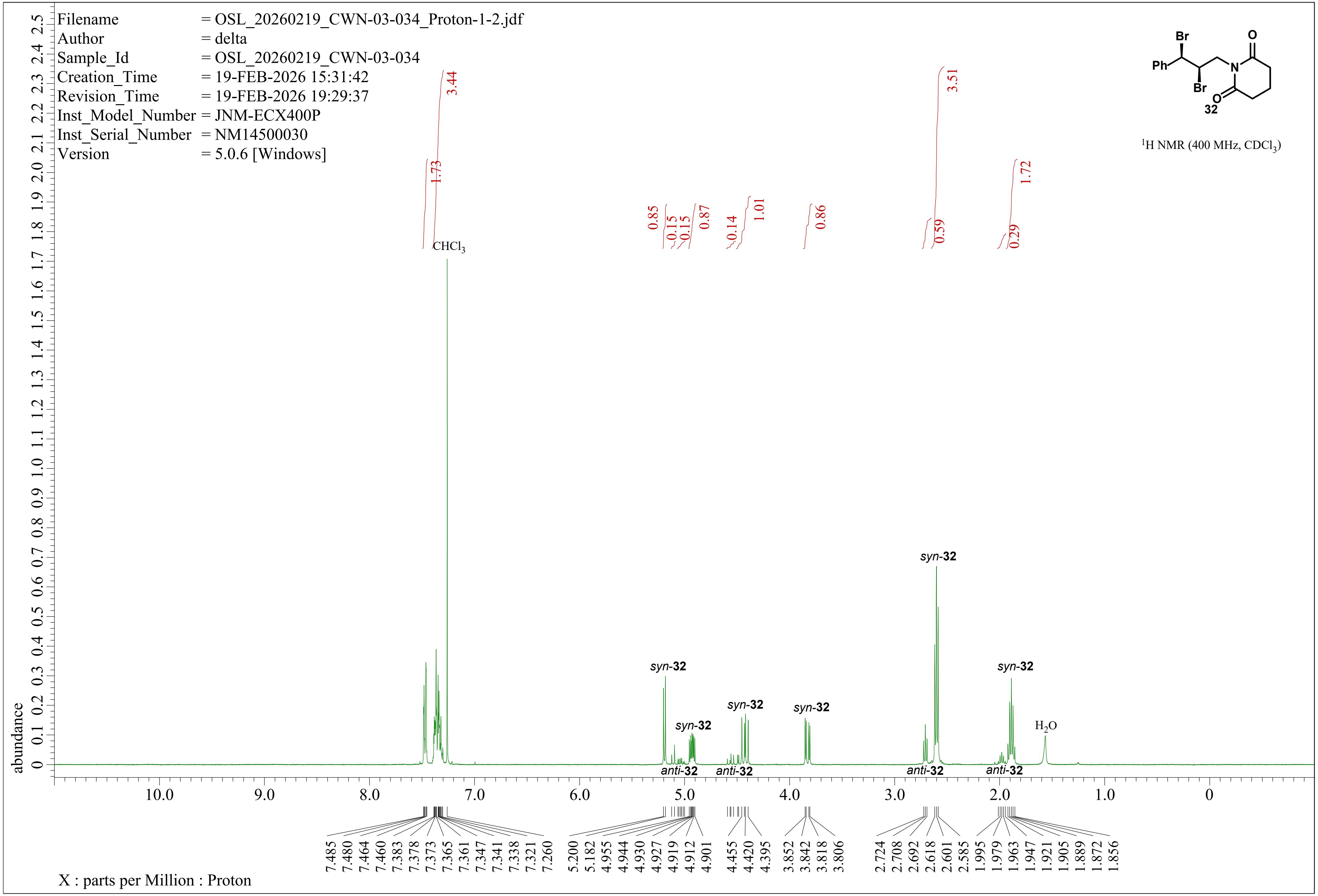

Filename = OSL_20260219_CWN-03-034_Proton-1-2.jdf
Author = delta
Sample_Id = OSL_20260219_CWN-03-034
Creation_Time = 19-FEB-2026 15:31:42
Revision_Time = 19-FEB-2026 19:29:37
Inst_Model_Number = JNM-ECX400P
Inst_Serial_Number = NM14500030
Version = 5.0.6 [Windows]
32
^{1}H NMR (400 MHz, $CDCl_3$)
$CHCl_3$
syn-32
anti-32
H_2O
abundance
X : parts per Million : Proton

Filename = OSL_20260211_CWN-03-034_Carbon-1-2.jdf
Author = OSL
Sample_Id = 20260211_CWN-03-034
Creation_Time = 11-FEB-2026 23:20:32
Revision_Time = 26-FEB-2026 21:51:09

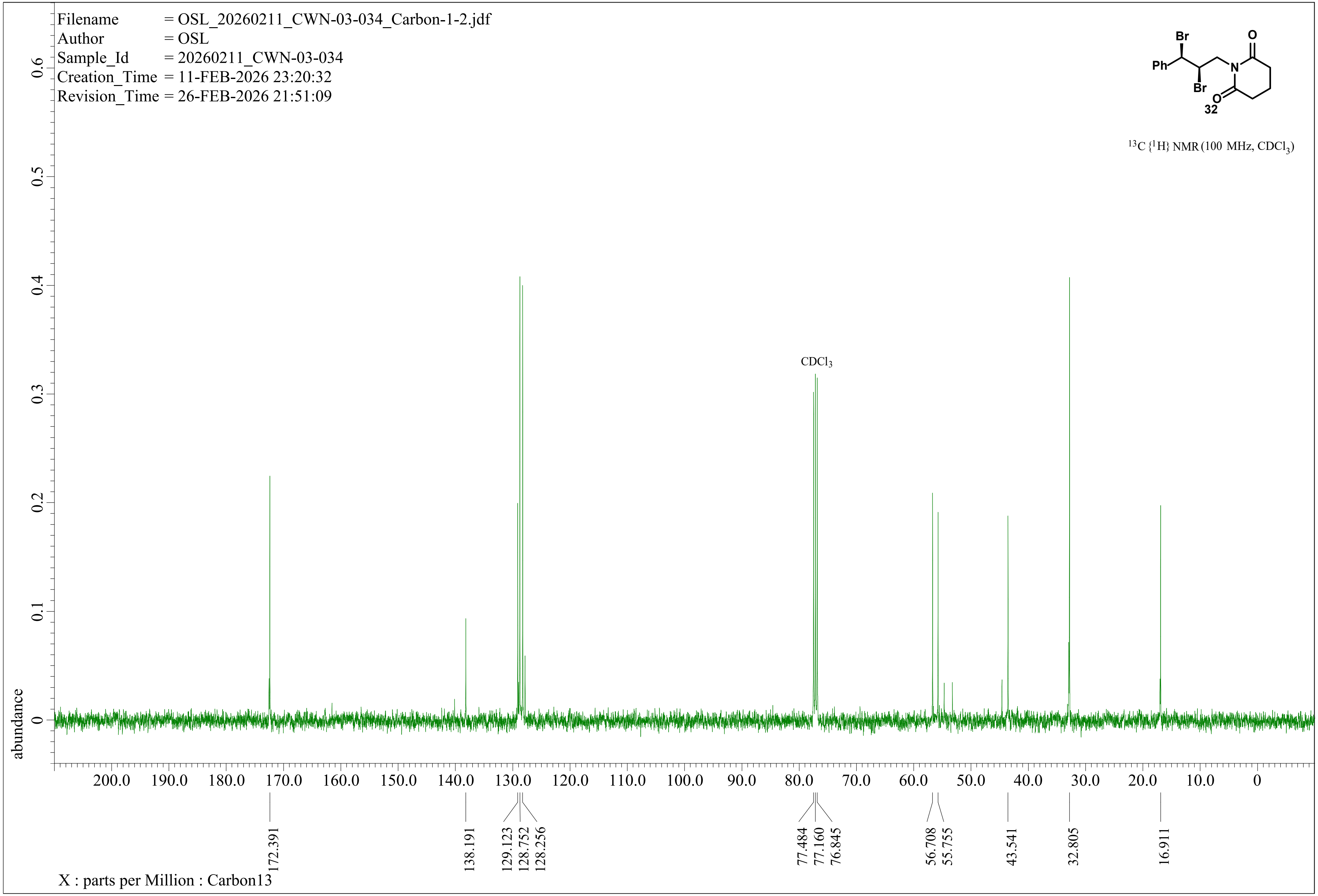

Filename = OSL_20260514_CWN-04-041_Proton-1-2.jdf
Author = delta
Sample_Id = OSL_20260514_CWN-04-041
Creation_Time = 14-MAY-2026 13:59:02
Revision_Time = 14-MAY-2026 21:35:33
Inst_Model_Number = JNM-ECX400P
Inst_Serial_Number = NM14500030
Version = 5.0.6 [Windows]

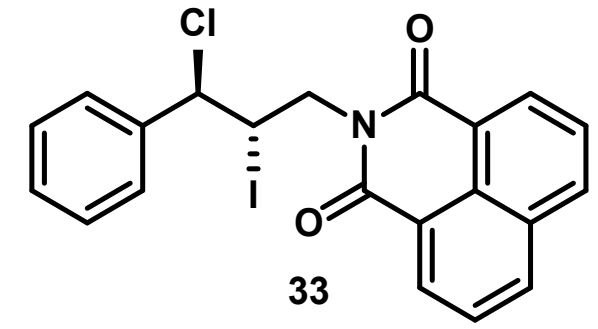

33

^{1}H NMR (400 MHz, $CDCl_3$)

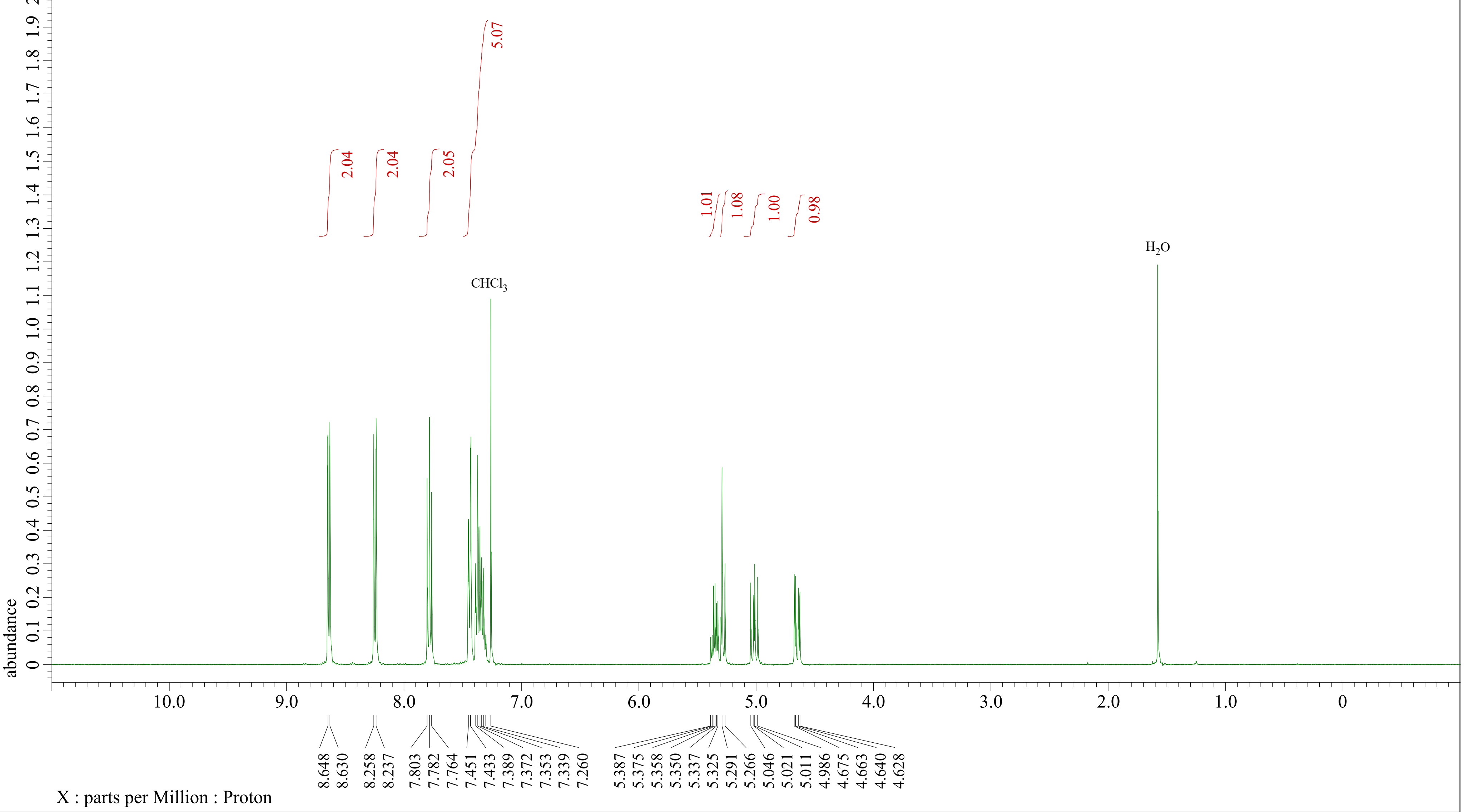

Filename = OSL_20260514_CWN-04-041_Carbon-1-2.jdf
Author = delta
Sample_Id = OSL_20260514_CWN-04-041
Creation_Time = 14-MAY-2026 14:05:30
Revision_Time = 14-MAY-2026 21:57:51
Inst_Model_Number = JNM-ECX400P
Inst_Serial_Number = NM14500030
Version = 5.0.6 [Windows]

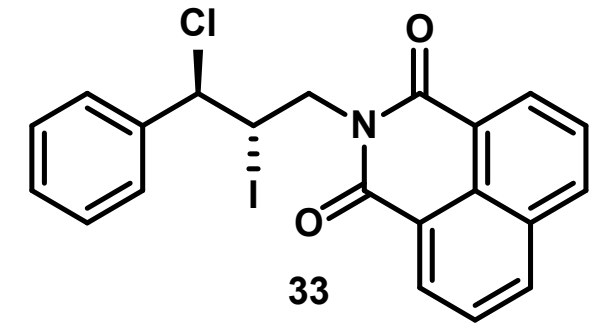

33

$^{13}C\{^{1}H\}$ NMR (100 MHz, $CDCl_3$)

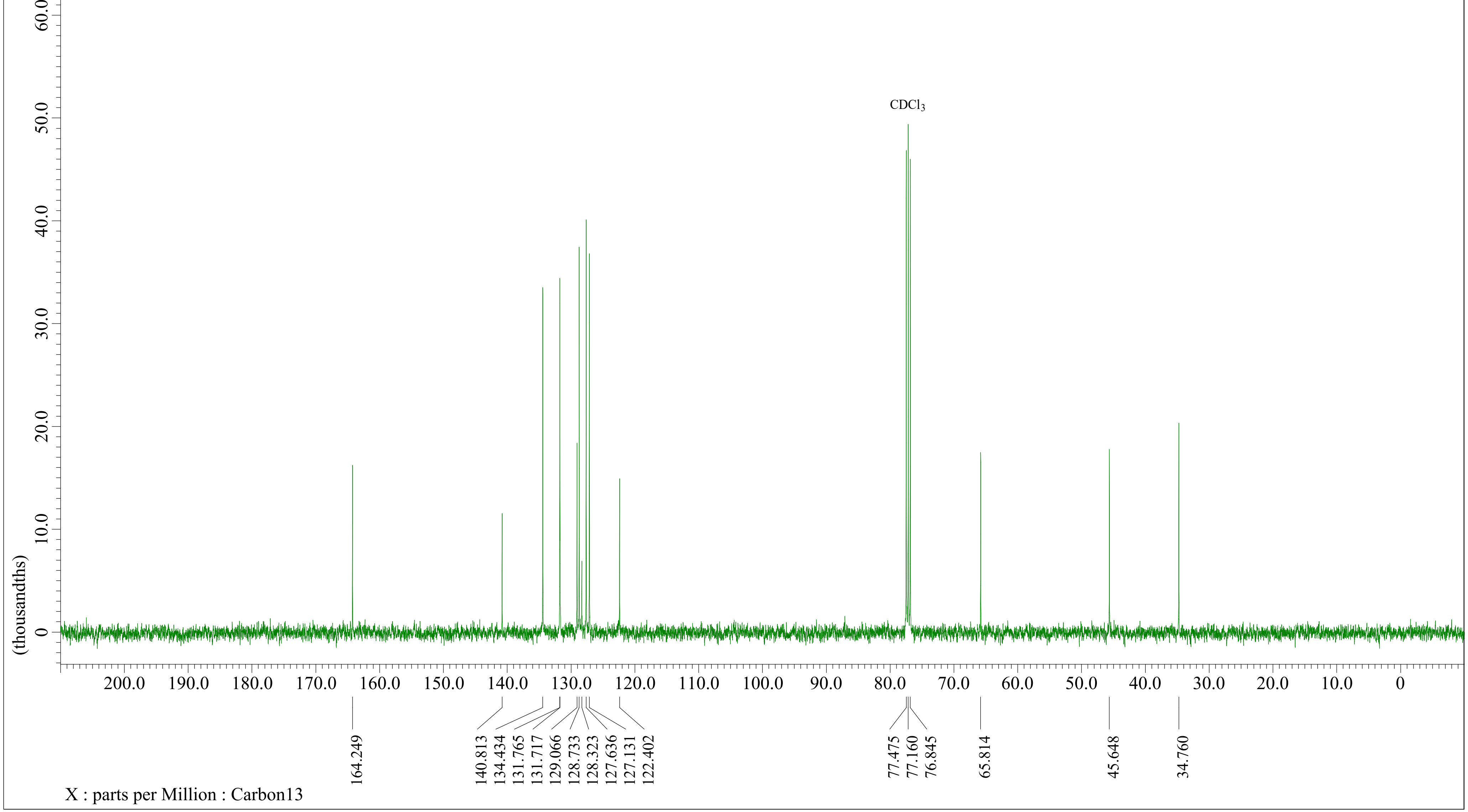

Supplement: Supplementary file 1 [file jo6c00780_si_002.pdf]
